# Recombinant Adeno-Associated Virus Vectors for Gene Therapy of the Central Nervous System: Delivery Routes and Clinical Aspects

**DOI:** 10.3390/biomedicines12071523

**Published:** 2024-07-09

**Authors:** Żaneta Słyk, Natalia Stachowiak, Maciej Małecki

**Affiliations:** 1 Department of Applied Pharmacy, Faculty of Pharmacy, Medical University of Warsaw, 02-091 Warsaw, Poland; 2 Laboratory of Gene Therapy, Faculty of Pharmacy, Medical University of Warsaw, 02-091 Warsaw, Poland

**Keywords:** gene therapy, central nervous system, clinical trials, PK/PD

## Abstract

The Central Nervous System (CNS) is vulnerable to a range of diseases, including neurodegenerative and oncological conditions, which present significant treatment challenges. The blood–brain barrier (BBB) restricts molecule penetration, complicating the achievement of therapeutic concentrations in the CNS following systemic administration. Gene therapy using recombinant adeno-associated virus (rAAV) vectors emerges as a promising strategy for treating CNS diseases, demonstrated by the registration of six gene therapy products in the past six years and 87 ongoing clinical trials. This review explores the implementation of rAAV vectors in CNS disease treatment, emphasizing AAV biology and vector engineering. Various administration methods—such as intravenous, intrathecal, and intraparenchymal routes—and experimental approaches like intranasal and intramuscular administration are evaluated, discussing their advantages and limitations in different CNS contexts. Additionally, the review underscores the importance of optimizing therapeutic efficacy through the pharmacokinetics (PK) and pharmacodynamics (PD) of rAAV vectors. A comprehensive analysis of clinical trials reveals successes and challenges, including barriers to commercialization. This review provides insights into therapeutic strategies using rAAV vectors in neurological diseases and identifies areas requiring further research, particularly in optimizing rAAV PK/PD.

## 1. Introduction

The central nervous system (CNS), particularly the brain, is at risk of developing several diseases of various etiologic origins. Limited access of pharmacological agents to the cerebral parenchyma, or the absence of drugs dedicated to specific causes, categorizes certain brain disorders as prognostically fatal and significantly diminishes the patient’s quality of life [1]. The unique biological characteristics of neural tissue, coupled with its limited capacity for self-regeneration, further exacerbate the difficulties of treatment. In addition, disorders of brain physiology are not restricted by age, as they can affect individuals across all age groups, including the youngest [2]. Among these disorders, neurodegenerative and neoplastic diseases pose the greatest difficulties in terms of treatment [3,4].

The CNS is protected by the blood–brain barrier (BBB), characterized by tight adhesion of endothelial cells. This barrier controls brain homeostasis and exhibits metabolic and transport functions. However, it also serves as a barrier to the transport of substances, including drugs, prompting numerous efforts to develop methods to modulate or bypass the BBB [5].

Gene therapy has been explored for various CNS diseases, including neurodegenerative and neurodevelopmental disorders, gliomas, and epileptic conditions [6,7,8]. Engineered gene therapy products offer diverse mechanisms of action that can be tailored to the specific pathophysiology of diseases, enabling the silencing, modulation, or even replacement of dysfunctional genes through gene editing.

Gene therapy techniques are primarily based on in vivo or ex vivo techniques [9]. The in vivo technique involves using a suitable viral or nonviral carrier to introduce the therapeutic gene directly into the patient’s body. While intravenous delivery is the most convenient and common route for in vivo gene therapy, its effectiveness for CNS diseases is limited by the ability of delivery systems to cross the BBB. Recombinant adeno-associated vectors (rAAVs) have emerged as the vectors of choice for CNS therapy [10]. These vectors have been extensively utilized due to their safety profile, stable gene expression, and some degree of neuronal tropism. In addition, certain rAAV serotypes, such as rAAV9 and rAAVPHP.B, have been identified for their ability to cross the BBB following intravenous administration [11]. Notably, a clinical report in 2020 on gene therapy for SMA type I demonstrated the safety and efficacy of intravenous application of rAAV9 vectors carrying the therapeutic gene (Zolgensma) [12].

Adeno-associated viruses (AAVs) from the *Parvoviridae* family are small viruses without a lipid envelope, containing single-stranded DNA as their genetic material. With a virion diameter of about 25 nm, AAV vectors are considered among the smallest animal viruses. The small size of AAV capsids restricts the size of transgene cassettes that can be packaged to about 4.7 kb, which is often cited as a major drawback of rAAV carriers. This limitation theoretically excludes the use of AAVs as gene vectors in therapies for diseases caused by mutations in large genes such as dystrophin (Duchenne dystrophy) or factor VII (hemophilia type A). However, a strategy involving viral DNA dimerization has been developed to overcome this limitation, known as the “split vector” method, which takes advantage of AAV vectors developing an episomal form [13].

AAVs possess several favorable properties that make them potential and attractive transgene carriers for human gene therapy. Unlike many other viruses considered for human use, wild-type AAV is nonpathogenic and exhibits low immunogenicity. Recombinant AAV vectors lack nucleotide sequences encoding viral proteins, unlike adenoviral carriers, and do not induce an inflammatory response; thus, they do not interfere with the efficiency of therapeutic gene expression. Furthermore, recombinant AAV vectors do not integrate into the host genome, instead adopting an episomal form, thus eliminating the risk of insertional mutagenesis associated with retroviruses [14]. Another advantageous characteristic of rAAV carriers is their ability to infect both non-dividing and dividing cells, making them suitable for use in brain gene therapy. AAV vectors can be produced in high concentrations and are stable during storage. With more than a dozen serotypes characterized by tissue tropism, the simple capsid structure of AAV allows for modification to target specific cells [15,16].

The efficacy of gene therapy using rAAV vectors depends not only on the appropriately selected therapeutic gene but also on the technique used to introduce the vectors into the patient’s body [17]. For CNS therapy, systemic or BBB-passing administrations, such as administration into the cerebrospinal fluid or directly into the brain parenchyma, are utilized. The tropism or direction of transport of rAAV vectors is dependent on the chosen administration technique.

## 2. Biology of AAV

The use of viral vectors for gene therapy is a valuable strategy [18]. Commonly used vectors include modified lentiviruses, adenoviruses, retroviruses, or adeno-associated viruses (rAAV) [19]. These vectors are chosen for their high efficiency in delivering genes to specific tissues or organs, long-lasting expression of introduced genes, and overall safety and tolerability. rAAV in particular meets these criteria.

**Figure 1:**
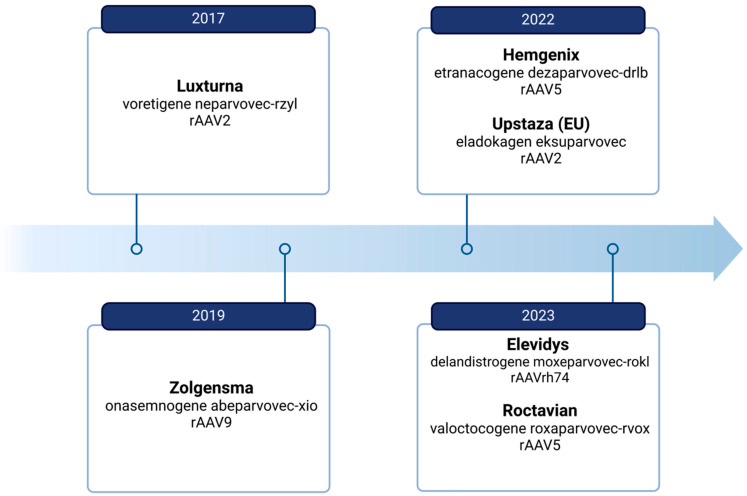
Currently registered gene preparations using rAAV. Created with BioRender.com.

The success of rAAV in gene therapy is evident from the large number of clinical trials conducted. As of March 2023, there had been 366 clinical trials involving rAAVs, accounting for 9.4% of trials in the gene therapy field. Successful trials have led to the development of gene drugs using AAVs as transgene carriers. As of March 2024, there are several formulations on the market containing rAAVs as shown in Figure 1.

AAVs have been recognized in the scientific world since 1965 when Bob Atkinson discovered small, antigenically distinct “impurities” while studying Adenovirus (Ad). It was noted that replication of these particles only occurs in the presence of Ad [20]. Subsequent studies confirmed this relationship and showed that coinfection with other viruses, such as Herpes simplex virus (HSV) and Cytomegalovirus (CMV), is necessary for the synthesis of AAV antigens [21,22]. This characteristic is also reflected in the taxonomic classification of AAV, which is included in the family *Parvoviridae*, genus *Dependovirus* [23].

The presence of antibodies against AAV in humans has been reported to be around 40–70% [24]. AAV can infect both dividing and nondividing cells [25]. These small viruses lack an envelope and have a size of about 26 nm, with a single-stranded genetic material consisting of 4.7 kb [26,27,28]. The AAV genome comprises sections including the promoter, polyA, replication-responsive genes (*Rep*), capsid structural genes (*Cap*), and assembly activation protein (*AAP*) genes [29,30,31]. At both ends of the genome, there are terminal repeats consisting of 125 nucleotides, known as inverse terminal repeats (*ITRs*) [29,32].

Recent discoveries by the group of Odgen et al. have revealed the existence of an additional gene with an open reading frame (ORF) shift of +1 in the *VP1* region. The expression of this gene leads to the production of a membrane protein called MAAP (Membrane-associated accessory protein), which functions as a viral exit protein [33,34]. The sequences encoding the nonstructural proteins responsible for replication are located in a common ORF with p5 and p19 promoters. The resulting Rep 78, 68, 52, and 40 proteins vary in size but contain a common amino acid stretch with a helicase/ATPase domain and a nuclear localization signal [35]. These proteins also contain a DNA-binding domain, an endonuclease, and a zinc finger domain, playing an essential role in packaging the viral genetic material into the capsid and thus facilitating efficient transfection of host cells [36].

The viral capsid is composed of approximately 60 proteins, with an estimated ratio of 1:1:10, formed by VP1, VP2, and VP3 units, respectively [37,38]. Alternative translation start sites and alternative splicing of the mRNA resulting from *Cap* gene expression allow for the formation of numerous capsid variants, leading to differences in the viral envelope [39]. The sequence encoding VP3 is shared by the other VPs and represents the largest pool of VPs that build the capsid, determining its icosahedral symmetry [40]. The other VPs, VP1 and VP2, which form the virus envelope, contribute to its unique structure and efficiency in transfection [41,42]. Understanding the biology and functions of individual capsid elements is crucial in developing appropriately modified viral envelopes to make gene therapy more effective and safer. When designing efficient constructs, attention is paid to the proper proportions of individual capsid protein subunits. For instance, in a baculovirus-based system, it has been observed that the efficacy of rAAV5 depends on the initiation of VP1 translation. An excess of VP3, as well as a low VP1/VP2 ratio, results in decreased vector efficacy [43]. In designing viral vectors, modifications are made to individual subunits. It has been demonstrated that the VP1/VP2 motif, similar to PLA2, is important for the limiting step in effective viral transduction, which is the escape from endosomes to the nucleus. In designing new capsid variants, Han et al. introduced changes to AAV8 and AAVS3, involving the attachment of PLA2-like motifs located in VP1, as well as modifications to the N-glycosylation sites in VP3, which are important for binding to cellular receptors. These changes resulted in higher transduction efficiency with reduced sensitivity to antibodies [44]. Modifications within VP3 result in different receptor affinities. It has been shown, using AAV2, that insertions within I-587 allow for changes in the capsid affecting its binding to cellular receptors. In studies by Girod et al., genetic modification was performed by introducing a ligand peptide into the viral capsid, thereby changing the affinity of AAV2 from its primary cellular receptor, HSPG, to an integrin receptor [42]. Work on ORF capsid mutagenesis has identified critical sites, including those involved in rAAV binding to receptors. It has been demonstrated that substitution of the serpin receptor ligand in the N-termini of VP1 and VP2 can change the tropism of the studied AAV2. The resulting vectors showed greater infectivity (insertion in VP2) and higher activity (insertion in VP1) compared to the wild type in IB3 cells [45]. Another example of VP2 modification is the incorporation of cell-specific ligands, such as DARPins (designed ankyrin repeat proteins) on the capsid surface. These are specific structures with antibody-like affinity, directing the vector to a specific receptor. In studies by Munch et al., functionality was given to specific cell types, such as Her2/neu [46]. It has been shown that proteins on the AAV capsid surface undergo phosphorylation, leading to ubiquitination and eventually viral degradation by proteasomes [47]. For AAV2, the introduction of mutated tyrosine and serine residues on its surface prevents phosphorylation, resulting in improved transduction. Another example is the substitution of threonine residues with valine, which also enhances transduction efficiency [48]. In studies by Kanaan et al., researchers made minor changes to the tyrosine residues of the AAV2 capsid, improving the vector’s transduction in the striatum and hippocampus. Additionally, modifications to the heparan sulfate receptors increased vector distribution [49]. An intriguing illustration of an AAV vector that exhibits modified distribution patterns is rAAV-2 retro. The majority of AAVs exhibit limited efficacy in facilitating retrograde transduction. Researchers, aiming to enhance the efficiency of rAAV vector transduction, have focused on retrograde transport from axons to projection neurons. This method is characterized by high cellular specificity and increased safety and broad distribution within the CNS [50]. AAV2-retro includes the insertion of LADQDYTKTA + V708I + N382D in the segment responsible for binding the heparin coreceptor in AAV2 [51]. The new AAV2-retro variant was compared in primates, specifically macaques, by introducing the vector into the caudate nucleus and putamen; it was demonstrated to have a significantly broader transduction potential than the original AAV2 variant, which was limited to the injection sites of the vector [52]. AAV2-retro is used, for instance, in the transduction of lower motor neurons after intramuscular administration (See Section 3.3). Encouraged by the work on AAV2-retro, researchers Lin et al. decided to construct a vector also characterized by retrograde transport. They introduced a 10-mer segment derived from AAV2-retro into the AAV9 capsid. Its efficacy was evaluated as comparable to AAV2-retro after intracranial and intravenous injections, while maintaining the ability to cross the BBB [53]. The taxonomic division of AAVs takes into account differences in the proteins of the icosahedral capsid [54], leading to the distinction of 13 major AAV serotypes [55,56]. The various tropisms of these serotypes result from differences in the structure of the structural proteins, allowing them to bind to specific cell surface receptors, such as glycans or proteoglycans. For example, the presence of n-linked sialic acid is necessary for the binding of AAV serotypes 1, 4, 5, and 6, while O-linked sialic acid is required for AAV5 binding [57,58]. Other receptors include heparan sulfate proteoglycans, which are necessary for the attachment of AAV serotypes 2, 3, 6, and 13 [59,60,61,62], as well as galactose, which is suitable for AAV9 attachment [63].

Primary receptors mediate access to specific protein coreceptors, such as AAVR (AAV 1, 2, 3, 5, 6, 8, 9) [64], GPR108 (all AAV serotypes except AAV5) [65], TM9f2 (AAV1, 2, 5, 6, 7, 8, 9) [65], LamR (AAV2, 3, 8, 9) [66], αVβ5 integrin and α5β1 (AAV2) [67,68], FGFR1 (AAV2, 3) [69,70], CD9 (AAV2) [71], HGFR (AAV2 and 3) [72], PDGFR (AAV5) [73], and EGFR (AAV6) [74]. A particularly important receptor is AAVR, also known as the KIAA0319L transmembrane protein. AAVR binds AAV via the Ig-like polycystic kidney disease (PKD) repeat domain (PKD2), facilitating rapid endocytosis from the cell membrane and movement into the Golgi apparatus [64,75]. It has been demonstrated that AAVR is involved in the transduction of most AAV serotypes. Another critical entry factor is a conserved protein identified as GPR108, which belongs to the G protein-coupled receptor superfamily. Knockout studies of the *GPR108* gene have shown that serotype 5 transduction is independent of this receptor [76]. GPR108 is primarily located in the Golgi apparatus [65].

The internalization of the viral vector can occur through various pathways. The clathrin-dependent or caveolin-dependent endocytosis pathway was the first to be described. Escape from endosomes, as demonstrated for AAV2, occurs under an acidic environment [23,77]. Another pathway for AAV virus internalization is the CLIC/GEEC pathway.

It has been shown that the entry of AAV2 into the cell requires the rearrangement of membrane cholesterol and the actin cytoskeleton, resulting in an endosome rich in GEEK protein, which is then transported to the Golgi apparatus [78]. Additionally, the virus can enter the cell interior via macropinocytosis. AAV2, after binding to HSPG and integrin αVβ5, activates the Rac1 and PI3 kinase cascade, facilitating its migration to the cell nucleus along the cytoskeletal network [79]. Upon entry into the cell, AAVs move toward the nucleus with the involvement of early, late, and recycling endosomes [80]. One study by Ding et al. showed that AAV2 is transported in a dose-dependent manner via recycling endosomes (Rab11) or late endosomes (Rab7) [81]. Transport into the nucleus may involve the Golgi apparatus [82]. Madigan et al. showed that disrupting the calcium gradient, through knockout of the calcium ATPase pump-building protein located in the Golgi apparatus, reduces the AAV transduction of various serotypes. This highlights the role of calcium in the intracellular transport of viruses and the conformational changes in capsids required for the efficient transduction of host cells [83].

In the next step, AAV must leave the endosome or Golgi apparatus and enter the cell cytoplasm. This process is facilitated by phospholipase A2 [42,84], which creates pores on the surface of membranes through its lipolytic action. Studies of the GPR108 protein indicate its role in AAV escape from the endosome [76]. AAVs accumulated in the perinuclear space enter the cell nucleus through nuclear pores, facilitated by the small GTPase Ran and cell karyopherins [85]. Various papers suggest that AAVs enter the nucleus before capsid removal [23,86], binding to nuclear importers such as IMPα [87]. Research by Grieger et al. suggests that basic regions (BRs) present on the capsid contain elements of nuclear localization signals (NLS). Among the four core regions, BR3 and BR4 are significant for infectivity and viral assembly [88].

Successful transcription requires the conversion of single-stranded viral DNA to its double-stranded form. This step is rate-limiting and can occasionally lead to ineffective infection [89]. In the absence of helper viruses, the processes from entry into the nucleus to the synthesis of the second strand are limited. Host factors such as the PHD5 finger domain protein and U2 snRNP-related proteins are involved in this process, and their silencing leads to an increase in AAV genome expression [90]. AAVs with *ITR* and *Rep* segments can integrate into the human genome at a site known as AAVS1 on chromosome 19 [91,92,93].

## 3. Routes of rAAV Administration in Nervous System Therapy

There are several routes of administration for therapeutic rAAV products targeting the CNS. The choice of rAAV vector delivery method depends on the type and location of the disease, patient age, adverse effects associated with the route of administration (ROA), adverse effects related to rAAV, and the serotype-specific properties of the vectors. Intravenous administration of rAAV vectors represents a convenient method for delivering therapeutics, especially in young patients suffering from spinal-cord-localized diseases such as SMA. Serotypes 9 and rh.10 are preferred vectors due to their ability to penetrate the BBB. In contrast, intramuscular administration is chosen for treating neuromuscular diseases, primarily those affecting the peripheral nervous system such as ALS. Serotypes predisposed to this type of administration include vectors characterized by their ability for retrograde transport, such as AAV2-retro. Clinical studies evaluating the efficacy of intraparenchymal administration of rAAV vectors primarily focus on neurodegenerative diseases with localized etiology, such as Parkinson’s disease (PD). The most commonly chosen serotype for this route of administration is rAAV2, which exhibits limited diffusion from the injection site, thereby minimizing the risk of off-target transduction. Intracerobrospinal fluid administration is a method that allows for the evasion of neutralizing antibodies targeted against rAAV vectors. This route of administration reduces the risk of adverse events associated with off-target transduction. Depending on the site of vector administration (intraparenchymal, intracerebroventricular, or intrathecal), it is associated with varying risks of route of administration-related complications The most commonly utilized serotypes for this route of administration are the neurotropic serotypes rAAV9 and rAAVrh.10.

### 3.1. Systemic Administration (Intravenous Administration)

The presence of the BBB presents a significant challenge to clinicians and researchers, as it limits the penetration of therapeutic agents into the brain, thereby limiting the effectiveness of systemic CNS therapy. Developing therapeutic agents that can overcome the BBB or increase its permeability is crucial for optimizing CNS therapy. rAAVs have shown promise as gene carriers for treating CNS diseases. The identification of a rAAV9 vector capable of overcoming the BBB, along with the development of protein engineering techniques (such as directed evolution), has led to the construction of several rAAV vectors. These vectors, when administered intravenously, can overcome the BBB and transduce CNS cells (See Table 1). Here are several strategies to enhance the effectiveness of CNS gene therapy using intravenously administered rAAV gene therapy products.

#### 3.1.1. Natural BBB-Crossing AAV

Intravenous administration is considered the most attractive route for drug delivery due to its high patient acceptance, low cost, and low risk of complications. However, systemic administration of viral vectors for CNS gene therapy is challenging because most vectors cannot penetrate the BBB. The discovery of a specific serotype among rAAV vectors that can effectively penetrate the BBB after intravenous administration has sparked numerous studies on CNS gene therapy using rAAV.

The neurotropic serotype rAAV9 has been identified as capable of transducing cells within the CNS, with studies considering the impact of the BBB on transduction [11]. Evaluations have involved intravenous administration of AAV9-*Gfp* vectors to neonatal and adult mice with fully formed BBBs, yielding different results that suggest the BBB’s role in limiting substances delivered to the CNS. In neonates, where the BBB is not fully developed, AAV9 preferentially transduced neurons, particularly motoneurons. In contrast, in mature individuals, the transduction profile was directed toward astrocytes, which mainly constitute the structural component of the BBB.

The mechanism by which rAAV vectors exit the BBB is not yet fully understood. Coadministration of rAAV vectors with an osmotically active compound, which increases BBB permeability, did not enhance barrier penetration. This suggests that transporters on the BBB facilitate active transport rather than passive diffusion [94]. Transporters like GLUT1 and MCT1, present on the BBB, are being investigated as potential binding sites for AAV9 [95]. The receptor for laminin (LamR) [66] has also been identified as important for efficient cell transduction by the rAAV9 vector. Laminins and their receptors are predominantly found in the CNS, with receptors localized in astrocytes, pericytes, neurons, endothelial cells, and progenitor cells [96].

The expression of laminin receptors in the CNS may indeed explain the efficiency of transduction achieved with the rAAV9 vector. However, results from intravenous administration of rAAV9 vectors at age-dependent times in primates did not align with those obtained in rodents. In primates, the transduction profile was found to persist over time and was notably more efficient in glial cells. The authors detected transgene expression, including in brain glial cells and spinal cord motor neurons, suggesting a potential application for diseases like spinal muscular atrophy (SMA) [97].

In 2019, the FDA approved the first gene therapy for children under 2 years old with SMA. This therapy, called Zolgensma, uses an intravenously administered gene preparation with the rAAV9 vector as a carrier for a fully functional *SMN1* gene. This approach efficiently delivers a correct copy of the gene to spinal cord motor neurons. A single dose of Zolgensma has been shown to improve muscle function and survival in SMA patients [98]. 

To date, rAAV9 is the primary unmodified viral vector considered for CNS targeting after intravenous drug application. It effectively transduces spinal cord motor neurons, leading to a significant reduction in clinical symptoms, improvement in quality of life, and prolongation of life in SMA patients. Among the vectors noted for their use in clinical trials due to their ability to penetrate or bypass the endothelium are AAVrh.10, in addition to AAV9 [99]. Studies by Zhang et al. and Yang et al. evaluated CNS transduction following the intravenous application of several serotypes of rAAV vectors in newborn and adult mice. The authors indicate that rAAVrh.10, rAAVrh.39, and rAAVrh.43 serotypes, when administered intravenously, exhibit CNS transduction capacity and cell tropism comparable to rAAV9 [100,101]. Additionally, Tanguy et al. highlighted the higher efficiency of the AAVrh.10 vector in CNS transduction after systemic administration [102]. Their study in a newborn mouse model showed that AAVrh.10 achieved similar or higher transduction than AAV9 in all brain areas tested. Statistically significant, higher transduction efficiency using AAVrh.10 compared to AAV9 was observed in the medulla oblongata and cerebellum. AAVrh.10 was more efficient in transducing the dorsal spinal cord and lower motor neurons. Interestingly, the differences between the serotypes appeared mainly at low doses, and increasing the dose did not improve the distribution of AAVrh.10 in the spinal cord, unlike AAV9.

Yang et al. conducted a study to evaluate the AAVrh.10 vector using a nonhuman primate [101]. They found that intravenous administration of rAAVrh.10-*eGfp* in adult marmosets led to the transduction of motor neurons throughout the spinal cord, oculomotor nucleus, and neurons in the dorsal root ganglia.

Although published data from clinical trials involving AAVrh.10 mainly involve routes of administration other than intravenous [99], a phase I clinical trial based on intravenous delivery of AAVrh.10-*GLB1* is currently underway (NCT04693598). However, the results of this trial have not yet been published.

#### 3.1.2. BBB-Crossing AAV9 Variants

Six approved gene therapy products are based on naturally occurring AAV serotypes: Glybera (AAV1), Luxturna (AAV2), Zolgensma (AAV9), Upstaza (AAV2), Hemgenix (AAV5), and Roctavian (AAV5) [103,104,105,106,107]. Published data indicate the significant potential of directed molecular evolution or Rational Design Strategies in developing synthetic rAAV capsid variants [108,109]. In 2016, Sourav R Choudhury et al. developed a new vector, AAV-AS [110], by inserting a polyalanine peptide at the N-terminus of the VP2 protein of the rAAV9 vector. Systemic administration of this new variant to adult mice demonstrated higher CNS transduction efficiency compared to the starting vector, with AAV-AS being 6-fold and 15-fold more effective in the spinal cord and brain, respectively.

Despite identifying several factors that may be involved in the transportation of rAAV9 across the BBB, the precise mechanism by which AAV9 crosses this barrier remains undetermined. The authors conducted a study on CHO cells and found that the binding mode of AAV-AS and AAV9 is identical, suggesting that the polyalanine peptide does not affect the interaction with receptors. However, the paper also notes that the impact of polyalanine residues on the interaction of the AAV-AS capsid with coreceptors on the luminal surface of brain microvascular endothelial cells or other cells along the BBB and cerebrospinal fluid barrier is currently unknown, and further studies are required.

In 2016, another AAV9 variant with increased affinity for the CNS was developed. AAV-PHP.B was created using the CREATE method [111], enriching the VP1 protein of the AAV9 capsid with a seven amino acid sequence (7-mer) in a randomized sequence. Studies showed that AAV-PHP.B transduces the entire CNS region of C57BL/6 strain mice, including neurons and glial cells, after intravenous administration. The transduction efficiency of AAV-PHP.B is approximately 40-fold to 92-fold higher than that of AAV9, depending on the area analyzed. This higher transduction efficiency was also confirmed in human neurons and glial cells in vitro. However, in a large animal model (NHPs), the evaluation of CNS transduction following intravascular administration of the AAV-PHP.B vector appeared to be less efficient. Matsuzaki et al. indicated that the only noticeable difference between AAV-PHP.B and AAV9 is the markedly higher transduction of peripheral dorsal root ganglion neurons, while the CNS transduction efficiency of AAV-PHP.B is comparable to AAV9 vectors [112]. Juliette Hordeaux et al. indicates that the neurotropism of the AAV-PHP.B vector is restricted to the C57BL/7 mouse strain on which it was evaluated [113]. Attempts to transduce the CNS using a different mouse strain, such as Balb/c, proved ineffective. This phenomenon was clarified in 2019 when the receptor for AAV-PHP.B, Ly6a, a GPI motif-anchored protein, was discovered to be expressed at the BBB in C57BL/6 mice but not in the Balb/c strain. Ly6a is responsible for transporting the modified vector across the BBB [114].

Subsequently, the CREATE method used to create AAV-PHP.B was used a year later to create a more efficient variant, AAV-PHP.eB [115]. While both vectors comparably transduce glial cells, the AAV-PHP.eB variant shows a 55% and 69% higher efficiency against cortical and striatal neurons, respectively. The modified variant also requires the presence of the Ly6a receptor protein. AAV-PHP.B and AAV-PHP.eB have been characterized as the most efficient CNS transducing vectors after intravenous administration, based on studies in mouse models.

Another variant of the rAAV9 vector with targeted CNS transduction and reduced affinity for peripheral tissues and organs is AAV9.HR [116]. This variant’s vector capsid differs from the parent vector by only two amino acid residues.

In 2019, the AAV-F vector, also a variant of AAV9, was developed [117]. The transduction efficiency of astrocytes and cortical neurons using AAV-F is 65-fold and 171-fold higher, respectively than that obtained with the initial AAV9. Transduction was found to be independent of mouse gender and strain (C57BL/6 and BALB/c), making AAV-F a very useful capsid for mouse CNS transduction. It is worth noting that the transduction efficiency was compared with that obtained with AAV9-PHP.B, which was found to be slightly higher.

In 2021, further AAV9 variants created by the rational design method appeared [118]. The authors developed AAV.CPP.16 and AAV.CPP.21, which expresses cell-penetrating peptides on the capsid surface. Systemic administration of these new variants resulted in a 6-fold to 249-fold increase in CNS cell transduction efficiency in four strains of mice and a 5-fold increase in cynomolgus macaques compared to the parental AAV9 vector. AAV.CPP.16 retains tropism in juvenile and adult macaques and has been shown to have the potential to deliver antitumor cargo in a mouse model of glioblastoma [118].

#### 3.1.3. BBB Crossing Other than AAV9 Variants

Serotypes rAAVrh.8, rAAVrh.10, rAAVrh.39, and rAAVrh.43 are prominent among the vectors that efficiently transduce CNS after intravenous administration [100,101]. Among these, rAAVrh.8 stands out for its ability to globally transduce glial and neuronal cells in clinically relevant CNS regions, including the cerebral cortex, caudate nucleus, hippocampus, corpus callosum, and black matter [101]. The rAAVrh.10 vector has shown promise in traversing the BBB. Researchers have utilized this property to create a variant known as rAAV1/rh.10 [119], which efficiently transduces the CNS while reducing liver transduction with systemic administration. This modification has increased the safety of systemic application and reduced the risk of hepatotoxicity [119].

In 2016, researchers developed AAV2-BR1, a vector with high specificity and efficient transduction for the endothelial blood vessels of the brain and spinal cord [120]. This vector demonstrated transgene expression levels in the brain that were significantly higher (1000-fold) than in the liver, 100-fold higher than in the heart. Compared to wild-type rAAV2, the same dose of the AAV-BR1 vector induced 650-fold higher transgene expression in the brain [120].

Another notable vector is rAAV-B1, developed around the same time, which, when administered systemically to adult mice and cats, resulted in gene transfer to the CNS, transducing multiple subpopulations of neuronal cells. This chimeric vector, containing the VP3 capsid gene primarily derived from AAV8 or AAVrh.43, is considered more efficient than AAV9 in delivering genes to the mouse brain and spinal cord. Additionally, it shows reduced sensitivity to neutralization by antibodies present in human sera [121].

In 2019, Cia-HinLau et al. developed a novel variant of rAAV1 by introducing the amino acid sequence PHP.B to enhance its ability to cross the BBB (rAAV1-PHP.B). Intravenous administration of a single dose of AAV1-PHP.B expressing CRISPR resulted in targeted transgene activation in the brains of mice [122]. The utilization of AAV vectors alongside CRISPR tools is justified by their complementary attributes and capacities in gene therapy and genome editing. AAV vectors are renowned for their proficiency in delivering genes to target cells, encompassing both somatic tissues and the nervous system. Integration with CRISPR/Cas9 enables precise genome editing, affording specific modifications to DNA sequences with heightened precision and mitigated risk of off-target effects. This amalgamation not only introduces novel therapeutic avenues for addressing genetic diseases, cancers, and previously intractable conditions but also fosters exploration into gene function and biological mechanisms. Such endeavors contribute significantly to the ongoing evolution of biotechnology and molecular medicine.

Additionally, several potential shuttle peptides (molecular vectors) capable of transporting cargo to the brain without compromising BBB integrity have been identified [123]. These peptides significantly enhanced brain transduction after systemic administration of AAV8, with the best-performing peptide being THR. The enhancement of AAV8 transduction in the brain by THR was found to be dose-dependent, with neurons being the main target. THR directly binds to the AAV8 virion, enhancing its ability to cross the BBB. Further experiments showed that THR binding to the AAV virus did not affect the biology of AAV8 infection [123,124].

**Table 1:** rAAV serotypes, target specificity, and their advantages in CNS treatment following systemic administration.

AAV Origin	AAV Serotype	Receptors	Transduced Target	Animal Model	Advantages/Disadvantages vs. Parenteral Vector or AAV9	References
Natural	AAV9	LamR, MCT1, GLUT1, galactose	Brain glial cells, DRG neurons, spinal motor neurons	NHPs		[97]
	AAVrh.8	LamR	Glial and neuron cells of CNS	Mice (C57BL/6)	Reduced peripheral tissue tropism vs. AAV9	[101]
	AAVrh.10	Unknown	Spinal cord motor neurons	NHP (Marmoset monkeys)	Distribution remained unchanged with increasing dose vs. AAV9	[101]
	rAAVrh.39	Unknown	Glial and neuron cells of CNS	Mice (C57BL/6)	Reduced peripheral tissue tropism vs. AAV9	[100,101]
	rAAVrh.43	Unknown	Glial and neuron cells of CNS	Mice (C57BL/6)	Reduced peripheral tissue tropism vs. AAV9	[100,101]
AAV9 variant	AAV-AS	Unknown	Neurons, glial, and endothelial cells of CNS	Mice (C57BL/6)	6- and 15-fold more efficient (spinal cord and brain, respectively)	[110]
	AAV-PHP.B	Ly6a	Neurons and glial cells of CNS	Mice (C57BL/6)	40-fold to 92-fold higher transduction (only in mice)	[111]
	AAV-PHP.eB	Ly6a	Neurons and glial cells of CNS	Mice (C57BL/6)	Higher efficiency than AAV-PHP.B (only in mice)	[115]
	AAV9.HR	Unknown	Brain glial cells, DRG neurons, spinal motor neurons	Mice (C57BL/6)	Lower transduction	[116]
	AAV-F		Neurons and glial cells of CNS	MIce (C57BL/6 and BALB/c)	65- to 171-fold higher transduction	[117]
	AAV.CPP.16		Neurons and glial cells of CNS	Mice (C57BL/6J, BALB/cJ, FVB/NJ 129S1/SvlmJ), NHPs (cynomolgus macaques)	6- to 249-fold transduction increase (mice) 5-fold transduction increase (NHPs)	[118]
AAV2 variant	AAV2-BR1	Unknown	Endothelial blood vessels of CNS	Mice (C57BL/6)	650-fold higher brain expression, liver de-targeting	[120]
			Brain neurons	Rats (Crl:SD)	No EC transduction in cerebral vessels	[125]
DNA shuffling (AAV1, 2, 4, 5, 6, 8, 9, rh.8, rh.10, rh.39, rh.43)	rAAV-B1	Unknown	Neurons, glial and endothelial cells of CNS	Mice (C57BL/6J)	Sensitivity to antibodies reduced, 5.8- to 14.5-fold higher transduction	[121]
AAV1 variant	rAAV1-PHP.B	Unknown	Brain tissue (cell type not indicated)	C57BL/6J	More efficient transduction	[122]

### 3.2. Intranasal Delivery

Traditionally, the intranasal route has been used to deliver targeted drugs for treating respiratory diseases. This route has also been used in clinical trials involving cystic fibrosis patients for delivering rAAV vectors [126]. Interestingly, this noninvasive method of administration is also being investigated for treating CNS disorders. The motivation behind this approach is the direct connection between the nasal cavity and the brain, which allows molecules to bypass the BBB. Absorption of molecules occurs in the olfactory and respiratory epithelia. The routes of transfer of compounds through the olfactory area to the olfactory bulb via sustentacular cells or exposed olfactory sensory neurons. Alternatively, compounds can reach the brain through the nasal respiratory epithelium via the trigeminal nerve [127].

Therapeutic targets for intranasal AAV vector application include depression. Liu et al. used AAV vectors to deliver the gene encoding NAP, an eight-amino-acid peptide derived from the neuroprotective protein ADNP, which has shown neuroprotective effects in various neurological disorders [128]. After intranasal administration of *NT4*-*NAP*/AAV to isolated C57BL/6 mice, a significant decrease in immobility time in the forced swim test (FST) was observed. This suggests a promising therapeutic strategy for depressive disorders. Around the same time, another study was published on intranasal therapy for depression using brain-derived neurotrophic factor (*BDNF*) [129]. A fusion gene, *BDNF-HA2TAT*/AAV, was constructed for intranasal delivery of *BDNF* to the CNS through the nose–brain pathway. Intranasal application of *BDNF-HA2TAT*/AAV to mice subjected to chronic mild stress reduced depression, as indicated by the FST results. This treatment was associated with increased BDNF levels in the hippocampus. A follow-up study in rats with poststroke depression showed that intranasal administration of *BDNF-HA2TAT*/AAV increased *BDNF* mRNA and protein levels in the prefrontal cortex, leading to improvements in neurological function after intranasal gene therapy [130].

Intranasal gene transfer has also been used to treat Mucopolysaccharidosis type 1 (MPSI). Adult IDUA-deficient mice were intranasally infused with AAV9-*IDUA* vectors [131]. IDUA enzyme activity in the olfactory bulb of these mice was 50 times higher than that of wild-type mice. Intranasal treatment with AAV9-*IDUA* also reduced glycosaminoglycan deposits in the brain. Immunofluorescence analysis showed no evidence of vector spread to areas of the brain other than the olfactory bulb. The reduction in storage materials is believed to be due to the diffusion of enzymes from the olfactory bulb and nasal epithelium to deeper brain areas.

At 8 months of age, IDUA-deficient mice treated with gene therapy were indistinguishable from normal control animals, while untreated IDUA-deficient mice exhibited significant deficits in learning and navigation. A study conducted in 2021 compared the efficacy of gene therapy using rAAV9 vectors administered via different routes in a mouse model of mucopolysaccharidosis type I [132]. The vectors were administered by direct injection into the cerebral ventricles (ICV), intrathecal infusion (IT), or intranasal application (IN). Animals treated via ICV and IT administration showed IDUA enzyme levels that were 3- to 1000-fold higher than wild-type animals in all brain regions examined. Intranasally treated animals exhibited IDUA levels that were 100-fold higher in the olfactory bulb, with levels in other parts of the brain similar to wild-type levels. Glycosaminoglycan levels normalized in ICV-treated and IT-treated mice, while in IN-treated mice, they were normalized in the olfactory bulb or reduced in other brain regions. Treated animals, including those treated IN, were indistinguishable from heterozygous animals, while untreated IDUA-deficient animals showed significant deficits in learning and spatial navigation [132].

In another study using neurogenotherapy with AAV, there was an attempt to intranasally apply serotype 2 AAV. The authors developed AAV/*NT4 TAT CBD3* to evaluate the effect of calcium ion-dependent modulation of NMDA receptors on the therapeutic effect of Alzheimer’s disease (AD) [133]. The study utilized the APP/PS1 mouse model of AD, which exhibits strong pathophysiologies, including Aβ1–42 deposition, impaired tau protein levels (an indicator of AD progression), and reduced cognitive function. Based on biochemical, cellular, and behavioral studies, the researchers observed reduced levels of Aβ1–42 and phosphorylated tau protein, a decreased percentage of apoptotic cells in the hippocampus, and reduced cognitive decline compared to untreated animals.

Physical methods, such as ultrasound, are also being explored in studies of particle delivery to the brain to overcome the BBB. The Focused Ultrasound-Mediated Intranasal Brain Drug Delivery Technique (FUSIN) is a method that uses the intranasal route to deliver drugs directly from the nose to the brain, bypassing the BBB [134]. In 2022, Ye et al. utilized this method for the intranasal introduction of rAAV5-*eGfp* into the brains of Cr.NIH (Swiss) strain mice [135].

The authors compared several routes of administration as follows: intranasal (IN), intranasal with ultrasound targeted at the cortex or brainstem (FUSIN), intravenous followed by FUS treatment targeted at the cortex (FUS-BBBD), and intraparenchymal injection into the cerebral cortex (DI). FUSIN resulted in more than a 2000-fold higher efficiency of AAV5-*eGfp* delivery to the cortex compared to IN delivery. FUSIN also achieved transduction levels comparable to DI in the brain areas studied, indicating similar efficacy with a much less invasive application. Additionally, the comparison between FUSIN and FUS-BBBD showed that the intranasal route was more effective in delivering rAAV5-*eGfp* than the intravenous route using a localized ultrasound technique.

### 3.3. Intramuscular Injection—Spinal Cord Delivery

Recombinant adeno-associated viruses have shown promise in treating motor neuron diseases in animal models [136,137,138,139,140,141]. Intramuscular administration is often used to deliver the therapeutic transgene. While direct injection into the CNS is necessary for CNS disorders, less invasive techniques such as delivery into muscle and peripheral nerves are being explored for diseases involving the spinal cord, like amyotrophic lateral sclerosis and spinal muscular atrophy. This strategy is supported by the axonal transport of viral carriers into the cell bodies of motoneurons [142], possibly facilitated by vector binding to dynein-dependent cytoplasmic microtubules [143,144].

Intramuscular administration is appealing for gene therapy due to its simplicity and low invasiveness. Viral vectors can be taken up by peripheral nerve endings and retrogradely transported along axons to motor and sensory neurons, potentially reaching cells in the spinal cord and brain. Nicholas M. Boulis et al. compared the effects of rAAV and adenoviral vectors injected into sciatic nerves in rats. After 21 days, the rAAV-treated group showed significantly higher transgene expression in the spinal cord compared to the adenovirus group. In addition, the green fluorescent protein (GFP) reporter gene expression was detected 21 days after unilateral sciatic nerve injection in dorsal root ganglion neurons and the spinal cord [136]. Transduction of motoneurons and the spinal cord was also achieved after the intramuscular application of rAAV vectors. A single intramuscular injection of AAV-*GDNF* resulted in significant *GDNF* expression that persisted for at least 10 months in transduced gastrocnemius muscle, concentrated mainly at neuromuscular junction sites (NMJs). Moreover, transgenic *GDNF* was detected in motoneurons projecting axons into the muscle, indicating retrograde axonal transport [140].

Li-Jun Wang et al. conducted a similar study, demonstrating that bilateral intramuscular injection of the AAV-*GDNF* vector delayed disease onset by ~13% and prolonged the survival of ALS transgenic mice by ~14%. However, all mice eventually developed skeletal muscle weakness and atrophy after the onset of motor symptoms. Disease duration, assessed as the number of days from onset to end-stage, did not differ between ALS mice treated with the AAV-*GDNF* vector and controls [141].

Research on the intramuscular application of rAAV vectors has explored different serotypes, primarily 1, 2, 6, 8, and 9. Edmund R. Hollis 2nd et al. compared the efficiency of intramuscular administration of rAAV1 to rAAV6 serotypes. They found that rAAV1 was the most efficient vector in transducing motoneurons after both intramuscular and intranasal applications. Serotype 1 showed the highest level of retrograde transport, as evidenced by the transduction of motoneurons projecting axons to the muscles receiving the vector. For single-stranded AAV1 injected into the sciatic nerve, ten times the number of viral particles was required for detectable transgene expression compared to scAAV1 [139].

In primates, spinal cord transduction after intramuscular application of rAAV6 was evaluated [138]. Cells expressing *eGfp* were observed in an area of approximately 1 cm of the spinal cord 4 weeks after intramuscular injection. A significant proportion of motor neurons were eGFP-positive, with some spinal cord sections showing over 50% transduction.

For nervous system transduction through transmuscular application, serotypes 8 and 9 have been prominent. Zheng et al. evaluated the nervous system transduction efficiency of AAV8 in adult mice by intramuscular injection [145]. AAV8 demonstrated axonal transport ability, with efficient gene transfer to the white matter of the spinal cord, dorsal root ganglion neurons, and peripheral nerves, along with a small number of transduced spinal cord gray matter cells. In the case of dorsal root ganglion neurons, transduction was more expressed on the side of vector administration. In contrast, Benkhelifa-Ziyyat et al. showed that a single injection of scAAV9 into the gastrocnemius muscle of adult mice resulted in extensive transduction of motoneurons along the entire spinal cord, without restriction to MNs connected to the injected muscle. Spinal cord astrocytes and peripheral organs were also transduced, indicating vector distribution from the injected muscle to both the CNS and periphery. Intramuscular injection of scAAV9 vectors carrying the *SMN* gene in mice with spinal muscular atrophy led to high levels of transgene expression in both the CNS and periphery, increasing survival length from 12 days to 163 days [137]. In 2016, serotypes 6, 8, and 9 were compared in terms of transgene delivery efficiency and promoter effects on transgene expression [146].

The serotypes carried a transgene encoding green fluorescence protein (eGfp) under the control of a cytomegalovirus (*cmv*) or human synapsin (*hSYN*) promoter. Vectors were applied by injection into the biceps muscle of the hind limb of adult C57BL/6J mice. The viral genome was mainly detected in the biceps muscle (no statistically significant differences between serotypes) and the adjacent sciatic nerve on the same side of the body (AAV6 > AAV9). Lower levels of *eGfp* mRNA were detected in the dorsal root ganglia (AAV8 > AAV6, AAV9) and lumbar spinal cord (no statistically significant differences between serotypes) on the same side of the body. Sparse eGfp fluorescence was observed in the lumbar spinal cord on both sides of the body, especially with rAAV6 and rAAV9. There were no differences shown between the promoters used. The differences in transgene delivery efficiency after intramuscular administration of rAAV vectors into the spinal cord are likely due to differences in axonal transport, which depends on the serotype of the rAAV vector [147].

The natural serotypes of rAAV vectors delivered intramuscularly mainly transduce the motoneuron projecting to the muscle where the vector is applied. rAAV2-retro was effective in transducing lower motor neurons in the ventral horn of the spinal cord and motor nuclei of the brainstem with a single injection into a single muscle in neonatal mice [148]. Intramuscular injection of rAAV2-retro into lower motor neurons resulted in high transduction efficiency (57.04% ± 4.01%). Through a spinal cord injury model test, it was confirmed that rAAV2-retro injected through the muscle diffuses through the cerebrospinal fluid pathway to achieve extensive transduction of lower motor neurons. Zhilong Ch. et al. used a modified rAAV2-retro vector to assess the transduction of mouse motoneurons and compared it with rAAV1, rAAV2, rAAV5, rAAV6, rAAV7, rAAV8, and rAAV9 serotypes. rAAV2-retro showed the highest efficiency of retrograde transduction of lower motor neurons in the spinal cord compared to the other rAAV serotypes tested. It enables extensive transduction of lower motor neurons in the spinal cord and brainstem after a single injection in a single muscle (forelimb), reaching 57.04% ± 4.01%, and is not limited to lower motor neurons associated with the injected muscle. Efficient transduction of lower motor neurons is achieved by diffusion into the cerebrospinal fluid [148].

### 3.4. Intraparenchymal Injection

Injection of preparations into the brain parenchyma represents a technique for bypassing the BBB. Initially, was considered a convenient way to administer gene vectors to locally restricted disease-affected areas, such as Parkinson’s disease (PD) [149]. Administration of interstitial injection into the brain parenchyma has also been evaluated in disease models affecting a widespread area, such as brain storage diseases [149,150]. Interstitial injection of rAAV vectors is an effective means of administration into the CNS, as rAAV vectors are susceptible to axonal transport. The direction and extent of transport are serotype-dependent [147]. The use of serotype 2 is associated with anterograde transport [151], while serotype 6 is retrogradely transported along axons. Long-distance and retrograde axonal transport of some AAV serotypes (AAV1, -6, -8, and -9) have been reported, which may promote the spread of viral particles in anatomically connected areas of the brain [142,152,153]. AAV9 and AAVrh.10 are leading candidates for parenchymal infusion, not only because of vector distribution but also because they can be transported along axons with high efficiency [154,155,156]. The dose of viral vectors is lower concerning titers administered intravenously or into the cerebrospinal fluid [157]. Another positive aspect of IP administration is that it reduces the occurrence of a response from the patient’s immune system, which can significantly affect the safety and efficacy of gene therapy. The introduction of gene preparations into an immunologically privileged site (brain and cerebrospinal fluid) significantly reduces the risk of anti-AAV antibodies affecting the applied treatment [158,159]. In recent years, there have been several preclinical and clinical studies demonstrating the potential of interstitial rAAV injection for the treatment of CNS disorders. Most of the ongoing clinical studies on the use of rAAV in CNS gene therapy have been based on the interstitial administration of vectors. Animal models have included mice [160], rats [161], cats [162], dogs [163], and primates [157]. Arnaud Cressant et al. conducted a study in a mouse model of mucopolysaccharidosis type IIIB [164]. Interstitial administration of rAAV2 or rAAV5 vectors encoding *NaGLU* resulted in high enzyme activity, exceeding physiological values. Improved behavior was observed in the animals. Enzyme activity was observed outside the vector injection areas for both serotypes. NaGLU activity was higher, and distribution was wider after using AAV5-*NaGLU* vectors than from AAV2-*NaGLU* vectors [164]. Haiyan Fu et al. confirmed the effective and long-lasting activity of recombinant human *NaGLU* in all brain structures/areas of mice injected with the rAAV2 vector [165]. The interstitial administration of rAAV5-*NaGLU* was also evaluated in a canine model of MPS III. Animals received eight AAV serotype 5 vector deposits that induced α-N-acetylglucosaminidase production. Information on reproducibility, tolerability, appropriate vector type, and dose was provided [163]. The results supported clinical trial designs to evaluate interstitial gene therapy in Sanfilippo syndrome. An uncontrolled phase I/II clinical trial is investigating interstitial administration of rAAV5-*hNaGLU* in four patients with MPS IIIB (NCT03300453) [152]. Evaluation 30 months after vector injection indicated safety and good tolerance of the treatment used. The level of α-N-acetylglucosaminidase assessed in the cerebrospinal fluid is 15–20% of that in healthy children. Neurocognitive functions improved in all patients, with the youngest patient showing functions comparable to healthy children [166]. Direct access to the brain parenchyma offers the possibility of delivering the appropriate dose and titer of vectors that systemic administration requires to be increased. The method of direct interstitial injection has been under development for several years [167,168,169].

One technique that ensures adequate drug distribution and prevents backflow through the cannula is convection-assisted delivery (CED). CED is a direct method of delivering drugs to the brain through microcoils [170]. This method allows for control over the rate and volume of infusion, which, along with the number of infusions inserted, determines the volume of distribution [171]. Another technique used to aid in the delivery of rAAV viral vectors to the brain parenchyma is magnetic resonance imaging (MRI)-based neuronavigation [172]. MRI enables the control of cannula position and the ongoing diffusion of the vector.

### 3.5. Intracerebrospinal Fluid Administration

An alternative route to interstitial administration of rAAV vectors is administration into the cerebrospinal fluid (CSF). This route of administration is gaining traction in neurodegenerative disease research involving broader brain regions [173]. The introduction of the preparation into the CSF can be achieved by administration into the cerebral ventricles (ICV) [174], into the cisterna magna (ICM) [175], or the spinal canal (IT) [159]. Studies on the administration of viral vectors into the cerebrospinal fluid are justified, among other reasons, by the greater distribution of the vector compared to intraparenchymal administration. Studies on AAV application to the cerebrospinal fluid confirming the effectiveness of gene transfer to cells in different areas of the brain and throughout the spinal cord were conducted in different animal species such as rodents, pigs, dogs, and non-human primates (NHPs) [176,177,178,179,180]. One of the papers mentioned above evaluates the effect of the administration of an rAAV vector encoding human interferon-β (AAV-*hIFN*-β) on glioma growth. Peritumor parenchymal transduction with AAV-*hIFN*-β was extremely effective in eliminating GBM brain tumors [179]. The infiltrative nature of glioma cells can lead to tumor cells remaining outside the local therapeutic zone created by the parenchymal delivery of AAV vectors. A study in a mouse model of GBM by ICV infusion of AAV-*IFN*-β completely prevents tumor growth [181]. The data presented by the authors suggest that ICV injection of rAAV vectors encoding antitumor proteins is a promising approach deserving further consideration for the treatment of GBM. Distribution of AAV vectors into the cerebral cortex or spinal cord can be achieved by intracerebral ventricular (ICV) or lumbar (IT) injection, respectively. The lumbar intrathecal route allows only moderate transgene expression in the upper spinal cord and brain [180].

Studies in primates indicate that intra cisterna magna (ICM) injection results in the more efficient delivery of the transgene to the brain as well as the spinal cord [159,182,183]. However, intra cisterna magna delivery can be difficult to use in humans. The use of intracisternal delivery, especially when injecting free-hand (the use of head-mounted devices on infants is inadvisable), can lead to the risk of not delivering into the cerebrospinal fluid, but more importantly, can cause spinal cord injury and often fatal complications [184]. Methods have been developed as an alternative to direct delivery of gene therapy to the CM. A technique adapting an intravascular microcoil, which can be safely delivered intrathecally under fluoroscopy, has been used to treat two patients with Tay-Sachs disease (aged 30 months and 7 months) with AAV gene therapy. This delivery technique is a safe and minimally invasive alternative to direct infusion into the CM to achieve wide distribution of AAV gene transfer into the CNS [185]. Two clinical trials using cisterna magna infusions based on the rAAV9 vector have recently been initiated (RGX-111 gene therapy, rAAV9—*IDUA*, NCT03580083, RGX-121 gene therapy, rAAV9—*IDS*, NCT03566043). In clinical trials, the use of administration of rAAV vectors into the cerebrospinal fluid is down to rAAV9 and rAAVrh.10 serotypes. Sorrentino et al. evaluated the transduction profile obtained after intra cisterna magna injection of eight different serotypes of rAAV vectors (1, 2, 5, 7, 9, rh.10, rh.39, and rh.43) in pigs [186]. The rAAV9 was characterized as the vector most efficiently transducing the entire CNS, including transduction of neurons and glial cells of various layers of the cerebral cortex, basal nuclei, midbrain and brainstem areas, and motor neurons of the spinal cord. For other serotypes, a pattern of cell transduction was indicated, e.g., rAAVrh.10 manifested tropism to Purkinje cells. Bey K. et al. showed that delivery of AAV9 to the lumbar spinal cord leads to transduction of spinal cord motor neurons and neurons and glial cells of several brain areas. In contrast, the use of AAVrh.10 is associated with a low yield of transduced motor neurons [187]. Rosenberg et al. showed that the CNS transduction efficiency of an AAVrh.10 vector administered into the cerebrospinal fluid is site-dependent. The delivery of AAVrh.10 through the ICM leads to better distribution compared to ICV and IP [188]. In conclusion, the biodistribution of rAAV vectors after introduction into the CSF is dependent on the serotype and tropism of the vector. The site of the application and the chosen animal model are also important, affecting the pattern and efficiency of transduction.

## 4. A Review of CNS Gene Therapy Clinical Trials

A total of 643 studies were extracted from the ClinicalTrials.gov database. “AAV,” “adeno-associated”, “AAV1”, “AAV2”, “AAV5”, “AAV6”, “AAV8”, “AAV9”, and “AAVrh” were used as keywords. No time cutoff was applied. Studies unrelated to CNS therapy were excluded, totaling 490 trials. Duplicate search results were removed, totaling 55. During selection, observational, historical, and follow-up studies were excluded, totaling 10 studies. Additionally, one study on Rett syndrome was excluded due to its nature as an in vitro analysis using biological material from patients. Ultimately, 87 CNS gene therapy trials using rAAV vectors as gene carriers were identified. Information was collected on the route of administration of the gene product, the serotype of the rAAV vector used, the transgene carried, the vector dose, and disease categories/disease indications. In cases where clinical trial data availability was limited, data were obtained from published research papers, including previous preclinical studies using the same vector, presentations, and results published at scientific conferences, press releases, and websites of the responsible parties. Missing data have been marked as “N/A” Detailed data can be found in Table 2.

Eighty-seven clinical trials on the administration of rAAV for the treatment of CNS diseases were analyzed. In clinical studies involving rAAV vectors, a wide range of diseases has been considered, many of which are genetic, neurodegenerative, or storage disorders. Among the genetic diseases mentioned are Adrenomyeloneuropathy (AMN), Canavan Disease (CD), Giant Axonal Neuropathy (GAN), IGHMBP2 Related Diseases (IGHMBP2), Late Infantile Neuronal Ceroid Lipofuscinosis (LINCL), Menkes Syndrome (MS), and Spastic Paraplegia type 50 (SP50). These disorders are characterized by mutations in genes leading to various neurological and motor function disorders. Another group comprises neurodegenerative diseases, which are characterized by progressive degeneration of nerve cells, resulting in a gradual deterioration of cognitive and motor functions. Clinical studies focus on Alzheimer’s disease (AD), Frontotemporal Dementia (FTD), Huntington disease (HD), Parkinson’s disease (PD), Amyotrophic Lateral Sclerosis (ALS), and Multiple System Atrophy (MSA). Among the storage diseases mentioned are Gaucher Disease Type 2 (GD2), GM1 Gangliosidosis (GM1), GM2 Gangliosidosis (GM2), and Krabbe disease (KD). These disorders involve the accumulation of abnormal substances in nerve cells, leading to their damage and dysfunction. Additionally, other neurological disorders such as Spinal Muscular Atrophy (SMA), Rett Syndrome (RS), and Temporal Lobe Epilepsy (TLE) have been considered in the studies. Below, selected disorders, which represent the most significant portion of clinical trials, will be discussed. 

**Figure 2:**
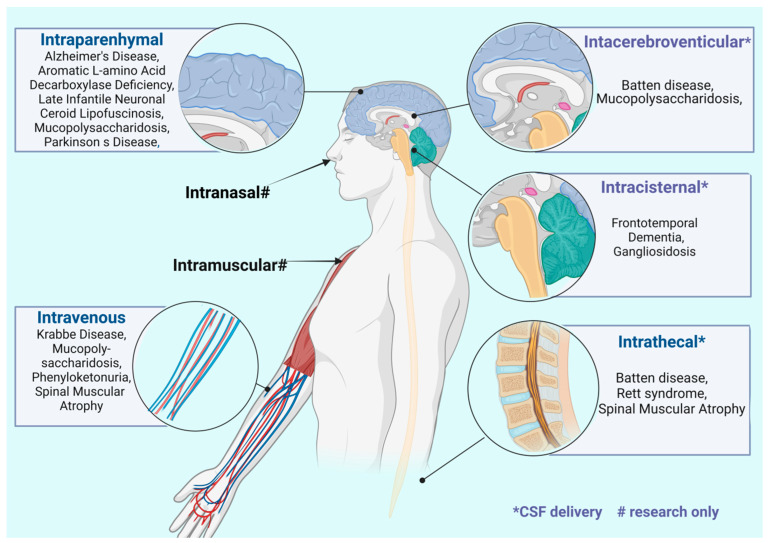
Routes of rAAV administration in CNS diseases along with the most common diseases found in clinical trials. Created with BioRender.com.

The largest number of clinical experiments focused on the treatment of PD (18.4%), spinal muscular atrophy (14.9%), and mucopolysaccharidoses (9.2%). Other studies using rAAV included Alzheimer’s disease (5.7%) and Aromatic L-amino acid decarboxylase deficiency (4.6%) (see Table 2). The data collected indicate that the main routes of administration used in clinical trials included direct interstitial administration (44.8%), intravenous administration (23.0%), and intrathecal administration (25.3%) (Figure 2). The most commonly used interstitial administration allows precise application to specific structures, such as the striatum, which is used in the treatment of PD, accounting for almost 38.5% of cases treated by this route.

PD is a neurodegenerative disease, and currently, available therapies focus on alleviating symptoms rather than eliminating causes. A variety of approaches are emerging in gene therapy, such as administering growth factors or inserting genes that encode correct versions of proteins. Overall, 6 of the 16 clinical trials for PD focus on introducing transgenes encoding growth factors, such as the neuritic trophic factor gene (*NTN*) or glial-cell-derived neurotrophic factor (*GDNF*) [189]. This strategy aims to regenerate, protect, and strengthen dopaminergic neurons in the nigrostriatal pathway.

In contrast, the remaining 10 therapies involve the insertion of transgenes containing a correct copy of genes encoding key enzymes associated with the development of PD. Attempts are being made to introduce a gene encoding aromatic L-amino acid decarboxylase (*AADC*) to restore normal dopamine biosynthesis, a glutamic acid decarboxylase (*GAD*) gene to regulate GABA production, and a *GBA* gene encoding glucocerebrosidase, which plays a role in sphingolipid metabolism. Patients with a mutation within *GBA* have been shown to have a higher likelihood quotient of rapid disease progression depending on the variant, ranging from 2.2 to 30 [190].

**Figure 3:**
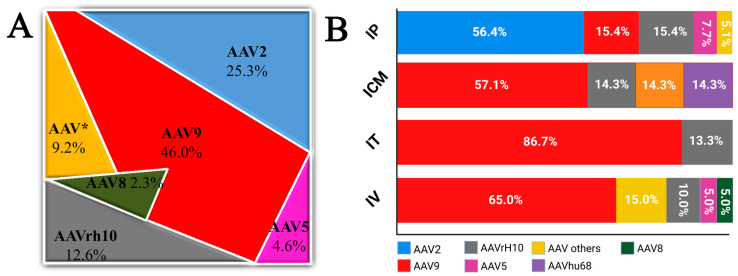
Participation of rAAV serotypes in CNS disease clinical trials. (**A**) Percentage distribution of rAAV vector serotypes used in clinical trials. (**B**) Percentage distribution of rAAV vector serotypes depending on ROA. IP, intraparenchymal; ICM, intra cisterna magna; IT, intrathecal; IV, intravenous administration, *, Serotypes not listed. Created with BioRender.com.

One of the strategies mentioned above for marginalizing motor dysfunction in PD is based on the insertion of additional copies of the normal gene encoding L-amino acid aromatic decarboxylase (*AADC*), which aims to restore normal biosynthesis of the neurotransmitter dopamine. In 2022, the gene therapy medicinal product eladocagene exuparvovec (Upstaza) received marketing authorization effective in the European Union. Upstaza delivers 2.8 × 10^11^ genome copies of rAAV2, containing the cDNA of the human gene encoding the enzyme DOPA-decarboxylase (DDC) under the control of the immediate-early cytomegalovirus gene promoter. Serotype 2 of rAAV vectors has been used in more than 56% of clinical trials based on interstitial administration (Figure 3). 

The proportion of other serotypes does not exceed 20%. Serotype rh.10 and serotype 9 were used in more than 15% of clinical trials. Other serotypes, such as AAV5, accounted for single clinical trials of intraparenchymal administration. Serotype 2 (AAV2) has been predominantly used for CNS disease studies due to its efficient neuronal transduction, longevity of transgene expression, and clinical safety [191]. AAV2’s affinity for HSPG, widely expressed in the brain parenchyma, limits the spread of the vector beyond the site of administration [192]. In contrast, AAV serotypes, including AAV9, which do not bind to HSPG, show increased spread after direct administration to the brain. rAAV2 appears to be an effective vector for local CNS gene therapy.

Parenchymal delivery requires specialized stereotactic neurosurgery procedures under controlled aseptic conditions. However, the direct introduction of drugs into the brain parenchyma offers significant advantages. This approach does not require consideration of the BBB, which limits the delivery of particles to the parenchyma and allows for precise delivery of the transgene to the target structure, potentially modifying or reducing the therapeutic dose. The doses used in the clinical trials analyzed span four orders of magnitude, ranging from 5.0 × 10^9^ to 6.0 × 10^13^ gc in Parkinson’s disease and Huntington’s disease, respectively. Only available data and those expressed in gc/dose were considered, while data accounting for individual variability (e.g., gc/mass brain) were omitted. As previously mentioned (Section 3), the introduction of gene preparations into immunologically privileged sites, such as the brain or cerebrospinal fluid, significantly reduces the risk of anti-AAV antibodies affecting the treatment.

Intravenous administration of CNS-targeted gene therapy formulations is the second most common method used in clinical trials. It is characterized by a low degree of invasiveness, and high patient acceptance, and does not require specialized medical equipment, which is important from the perspective of clinical practice. However, there are drawbacks, including the need for the rAAV serotype to effectively penetrate the BBB and deliver the transgene to the targeted CNS area, as well as the risk of peripheral tissue transduction generating side effects such as liver toxicity. rAAV9 is distinguished by its ability to effectively pass through the BBB, allowing it to penetrate the CNS. Therefore, rAAV9 is the most commonly selected serotype for the design of intravenous therapy for neurological diseases (65% of clinical trials), such as spinal muscular atrophy. Studies involving rAAV9 delivering a correct copy of the *SMN1* gene account for 50% of the clinical trials analyzed. The effectiveness of the vector in delivering the transgene to the CNS is evidenced by the formulation registered in May 2019 with the trade name Zolgensma, an onasemnogen abeparvovide delivering 1.1 × 10^14^ gc/kg patient weight by intravenous infusion [107]. The proportion of SMA-related therapies in clinical trials is about 14.9%. In all trials, the carrier is serotype 9 of rAAV vectors. A total of 3 of the 13 SMA-related trials use intrathecal administration. The doses of the intravenous formulations analyzed are in the range of 7.0 × 10^11^ to 3.3 × 10^14^ gc (only available data and those expressed in gc/dose were considered, data accounting for individual variability, e.g., gc/kg, was omitted). The dose range is three orders of magnitude and is relatively high compared to interstitial administration. It has been shown that intravenous administration of a higher dose is associated with increased efficiency of effective CNS transduction compared to lower doses, where the area of transduction is limited to blood vessels and perivascular regions [193]. The relationship between dose level and route of administration is evident in clinical trials targeting therapy of mucopolysaccharidoses, among others. Mucopolysaccharidoses (MPS) are a group of rare metabolic diseases caused by deficits in enzymes involved in the degradation of glycosaminoglycans (GAGs). There are several different types of MPS, each caused by a defect in a different enzyme. Depending on the type of disease in the clinical trials analyzed, the genes encoding IDUA (iduronidase) conditioning the degradation of heparan sulfate (HS) and heparan dermatan (DS) was administered, SGSH (sulfamidase) conditioning the degradation of heparan sulfate (HS), NAGLU (N-α-acetylglucosaminidase) conditioning the degradation of heparan sulfate (HS) in the treatment of MPSI, MPSIIIA, MPSIIIB, respectively. Clinical trials for this disease account for 9.2% of the studies presented in Table 2. The predominant carrier in the highlighted clinical trials was rAAV9. Clinical trials involving the treatment of mucopolysaccharidoses using direct administration into the brain parenchyma involved lower doses, on the order of 1.0 × 10^10^–1.0 × 10^11^ gc compared to intravenous administration (1.0 × 10^12^–1.0 × 10^14^ gc).

The third most common method in terms of clinical trials for intervention in CNS diseases is intrathecal administration. The main targets for therapy are diseases such as SMA, Batten disease, and Rett syndrome. Most of the trials that have been undertaken have used the rAAV9 serotype, with two studies looking at the rAAVrh.10 serotype.

## 5. AAV Gene Therapy in the Nervous System: Clinical Trials and Barriers to Commercialization

**Figure 4:**
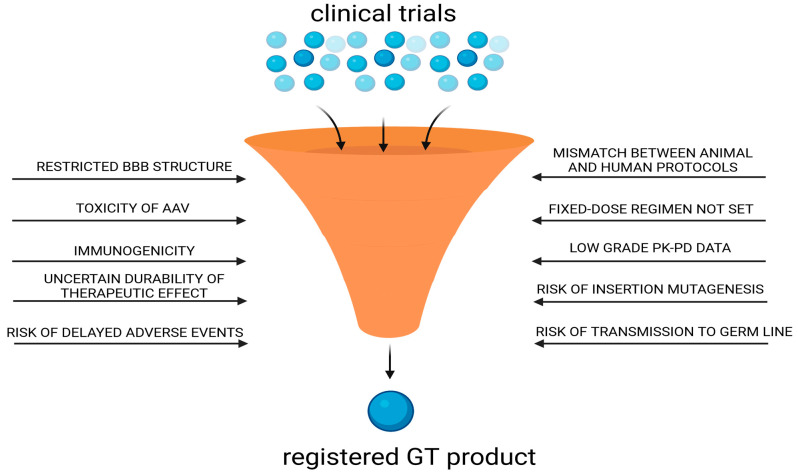
Missing aspects of AAV-mediated gene therapy to the nervous system in clinical trials. Created with BioRender.com.

As mentioned earlier, two gene therapy products based on rAAV vectors are registered for the treatment of Central Nervous System monogenic diseases—Zolgensma (onasemnogene abeparvovec) for treating SMA and Upstaza (eladocagene exuparvovec) for treating AADC [106,107]. The reason why only two rAAV-based gene therapy products for the CNS are registered is discussed below (see Figure 4). Diseases ideally suited for gene therapy are those that are monogenic and easily accessible for treatment. The CNS, particularly the brain, is prone to the development of numerous diseases with various etiological factors. Among them, neurodegenerative diseases and gliomas pose the greatest therapeutic challenges. The etiology of most CNS diseases is characterized by a combination of intricate genetic susceptibility and environmental factors [194,195].

Challenges in treating CNS diseases are exacerbated by limited access to brain tissue due to the physiological BBB [5]. The introduction of gene therapy products into the CNS can occur through local administration (intraparenchymal and intracerebrospinal fluid delivery) or systemic administration. Both routes of administration have disadvantages that limit the effectiveness and safety of therapy.

The most common route of rAAV administration in CNS gene therapy is intraparenchymal delivery, which bypasses the BBB. Parenchymal delivery requires specialized stereotactic neurosurgery procedures under controlled aseptic conditions, but allows for precise transgene delivery to target structures and potentially modifying therapeutic doses. Crafting a clinical protocol for delivering substances into the brain parenchyma is immensely significant and requires careful consideration of multiple factors (such as the region, presence of vital anatomical structures, number of injections, their volume, and infusion rate, potential side effect associated with ROA) (See Table 3). Tailoring these parameters from large animal brains to the human brain involves specific anatomical adaptations, including adjustments for brain size, cerebrospinal fluid volume, ventricular size. Additionally, pathological changes induced by disease can also impact alterations in vector biodistribution [196]. As mentioned earlier clinical trial doses range from 5.0 × 10^9^ to 6.0 × 10^13^ gc across Parkinson’s disease and Huntington’s disease (Table 2). Introducing of gene therapy product into the brain or cerebrospinal fluid, significantly reduces the risk of anti-AAV antibodies affecting the treatment [158,159]. Factors influencing the effectiveness of administration include barriers to transport after direct injection into the brain, such as diffusion through the extracellular matrix and availability of primary virus receptors at the site of injection [63,197]. Even in the case of local, targeted intraparenchymal rAAV delivery, it is important to consider the possibility of off-target adverse effects. The phenomena of vector diffusion and serotype-dependent axonal transport can influence the expected transduction efficiency at the site of administration as well as the average viral transduction volume (see Section 3) [53,197,198]. To mitigate the blood–brain barrier restrictions, neurotropic rAAV vector serotypes and intravenous administration are employed. As mentioned in Section 4, the second most common method used in clinical trials for administering CNS-targeted gene therapy formulations is intravenous administration. This method is characterized by a low degree of invasiveness, high patient acceptance, and does not require specialized medical instrumentation, which is important from the perspective of clinical practice. However, there are drawbacks associated with this method, such as the need for the rAAV serotype to effectively penetrate the BBB and deliver the transgene to the targeted CNS area, as well as the risk of peripheral tissue transduction generating side effects such as liver toxicity (see Table 3). rAAV9 and rAAVrh.10 are distinguished by their ability to effectively pass through the BBB, allowing them to penetrate the CNS (see Section 3 and Table 2). The intravenous dose of rAAV ranges from 7.0 × 10^11^ to 3.3 × 10^14^ gc/kg for CNS gene therapy clinical trials This dose range spans three orders of magnitude and is relatively high compared to interstitial administration. Higher doses administered intravenously are associated with greater efficiency in effectively transducing the CNS compared to lower doses, which result in limited transduction to blood vessels and perivascular regions [180]. Because the main target of intravenous delivery is the liver, the limitation of intravenous delivery is an immune response and hepatotoxicity after systemic exposure [199,200]. Also, other non-target organs, including muscle and heart, will undergo off-target transduction and potentially experience toxicities [201]. Immunogenicity and toxicity of rAAV represent substantial hurdles in the clinical advancement of rAAV gene therapies [201]. Weiran Shen et al. in a study analyzing 255 trials, indicate that there was a total of 11 patient deaths across eight trials, and 18 out of 30 clinical holds were due to toxicity findings in clinical studies. Additionally, 30.6% (*n* = 78) of trials had treatment-emergent serious adverse events (TESAEs), with hepatotoxicity and thrombotic microangiopathy (systemic delivery) and neurotoxicity (CNS delivery) being the most prominent [202]. Another aspect worth considering in the case of intravenous administration of rAAV is the decreasing permeability of the BBB with the patient’s age. For example, the efficacy of Zolgensma was evaluated in children up to 2 years of age or not exceeding a weight of 21 kg [107]. In adults, the neuronal transduction in the spinal cord and brain ranges from mild to nonexistent [157,203]. The transduction efficiency was significantly lower compared to intracerebroventricular or intrathecal administration, predominantly affecting glial cells, though the reasons for this remain unclear [94,101].

Intrathecal administration allows vectors to bypass the blood–brain barrier and requires a lower dose compared to intravenous administration (see Table 2). IT administration also carries a lower risk of triggering an immune response in patients to the gene therapy product. However, the primary concern associated with delivery to the CSF or via intrathecal injection is dorsal root ganglia (DRG) toxicity. This side effect has been repeatedly described in animal models [177], and DRG toxicity has also been observed in humans [204]. The toxicity induced by AAV in DRGs of non-human primates appears to be primarily driven by the AAV modality itself rather than by any specific therapeutic target. Moreover, neuronal degeneration/necrosis and nerve fiber degeneration have been observed in the trigeminal and autonomic ganglia of AAV-treated rats and cynomolgus monkeys, indicating widespread effects across various ganglia in the body [205]. Intrathecal administration in the lumbar region primarily targets the spinal cord and dorsal root ganglia [178]. To reach the brain target via the CSF stream, a larger dose volume is required [159]. When considering the technical aspects of administration, IT is easier for clinicians compared to ICV and ICM—it does not necessitate general anesthesia or an operating room [206]. However, it carries a higher risk of complications compared to intravenous administration (refer to Table 3).

Another key obstacle to the successful registration of a gene therapy product is establishing the appropriate vector dosing. The doses of vector delivered vary based on the route of administration. Range of doses used in nervous system gene therapy is 10^9^ to 10^13^ gc, 10^11^ to 10^14^ gc and 10^13^ to 10^15^ gc for intraparenchymal, intravenous and intrathecal administration, respectively (Table 2). There is a noticeable significant difference, even up to 4 orders of magnitude within one route of administration. To determine dose levels for clinical trials in humans, biodistribution data are typically generated during preclinical development and are required prior to conducting a first-in-human trial [207,208]. Animal models provide initial estimates of the safety margin and dosing parameters for human drug candidates. The transition from preclinical to clinical stages is complicated by anatomical and biological differences between species, as well as the absence of appropriate pharmacokinetic (PK) and pharmacodynamic (PD) models (see Section 6). These interspecies variations impede the translation and extrapolation of gene therapy product responses from animals to humans. Contributing factors include species specificity, immunogenicity, and differences in the regulation of transcription and translation of the transgene, all of which affect transduction efficiency [17,209,210].

Another factor that may potentially limit the registration of gene therapy medicinal products based on rAAV vectors is the occurrence of adverse events. Understanding the long-term outcomes and potential side effects of rAAV-based gene therapy is crucial for evaluating its overall efficacy and safety. While the short-term successes of rAAV vectors in gene therapy are well-documented, there is limited information on the durability of therapeutic effects and the occurrence of delayed adverse reactions. This section aims to provide a comprehensive overview of the available data on these important aspects, which pose challenges in treating CNS diseases.

Delayed adverse events refer to undesirable effects of medications, medical procedures, or therapies that do not immediately manifest after administration but appear after a period following the initiation of treatment or procedure [211]. Among various types of adverse events, thrombotic microangiopathy (TMA) is particularly noteworthy. For instance, Chand et al. documented a case series where three infants developed TMA following infusion with onasemnogene abeparvovec (Zolgensma). TMA manifested approximately a week after infusion, highlighting its delayed onset as an adverse reaction [212]. Recent studies suggest that thrombotic microangiopathy occurring after systemic administration of AAV is dependent on antibodies (through the classical pathway) and is exacerbated by the alternative complement pathway [212]. The underlying disease itself may contribute to such occurrences. Research indicates that children with spinal muscular atrophy (SMA) may exhibit coagulation abnormalities [213]. Factors that could potentially trigger such reactions include also concurrent infection with encapsulated bacteria, recent vaccination and prior administration of other gene therapy products [214]. It remains uncertain whether previous exposure to nusinersen poses any additional risks to onasemnogene abeparvovec [215].

The variability regarding how long the therapeutic benefits of rAAV vectors last after treatment, and the factors that influence this duration, pose challenges in treating CNS diseases. The longevity of the therapeutic response is crucial for the sustained success of long-term treatment, particularly considering that immune responses to rAAV vectors could hinder re-administration of the same therapy. Emerging evidence suggests that the effects of rAAV gene therapy can endure for extended periods. The reported durability of response for the first two approved human rAAV gene therapies, Glybera and Luxturna, extends to 6 and 7.5 years, respectively [216,217]. Behavioral recovery was observed in a monkey model of Parkinson’s disease 15 years after the administration of rAAV-based gene therapy to the putamen [191]. Similarly, stable therapeutic expression of factor IX was documented in individuals with severe hemophilia B for up to 8 years following systemic administration of an rAAV8 vector [218,219]. The durability of gene therapy may be influenced by two distinct decline mechanisms: rapid decline, possibly due to immune responses, and gradual decline resulting from vector dilution. While sustained transgene expression was achieved in most trials targeting the Central Nervous System (90.0%) and muscle (73.3%), only 43.6% of trials aimed at ocular conditions demonstrated enduring clinical efficacy [202]. The longevity of effectiveness varies significantly due to factors such as disease indication, dosage, serotypes, patient-specific factors, and the distinct turnover rates observed across different cell populations. Due to the host immune response to the initial therapy, redosing through systemic delivery is currently not feasible [19,199,202,220]. Consequently, rAAV gene therapies are presently constrained to target long-lived cells. Considering that episomes are unlikely to be inherited by daughter cells and are prone to loss during mitosis, the durability of transgene expression in the target tissue will be dictated by the rate of cell turnover. Myofibers undergo slow turnover, and muscle-directed rAAV therapy for α-1-antitrypsin deficiency in humans has shown stable transgene expression for up to 5 years after vector administration [221]. Neurons also exhibit minimal turnover rates, which is consistent with the Novartis presentation of new data showcasing the transformative and enduring benefits of SMA gene therapy, Zolgensma. Even after 7.5 years, the treated children continued to maintain their motor milestones [222]. The potential for vector genome integration also poses an uncertainty that could potentially impact the effectiveness of registering rAAV-based products. To address concerns regarding the efficacy and duration of transgene expression in liver-based adeno-associated virus gene therapies, researchers examined the potential for vector integration [223]. Studies have shown that in primate hepatocytes, AAV-mediated transgene expression occurs in two distinct phases: an initial phase characterized by high expression from episomal genomes, which is transient, followed by a subsequent phase with reduced yet stable expression, likely attributable to integrated vectors [223]. Additionally, an intriguing aspect observed in hepatotoxicity associated with AAV gene therapy is its delayed onset, typically occurring 4–8 weeks after dosing. This delay suggests the persistence of AAV capsid antigens in certain tissues, potentially triggering the activation of capsid-specific CD8 T cells [224].

Further concerns have been raised regarding the potential for AAV vectors to induce mutations upon insertion, which could potentially lead to cellular transformation [225]. Although most rAAV DNA typically remains episomal, a fraction integrates into genomic DNA at a low rate. Studies in neonatal mice have shown that rAAV insertion can induce tumor formation [226,227]. Currently, the risk of rAAV-mediated oncogenesis in humans is largely theoretical, as no confirmed cases of genotoxic events have been reported. Despite ongoing concerns about their potential genotoxic and carcinogenic risks associated with insertional mutagenesis, regulatory bodies like the FDA and EMA classify AAV vectors as non-integrating based on guidance documents. For instance, the FDA’s finalized long-term follow-up guidance from January 2020 asserts that AAV vectors are unlikely to integrate, thus minimizing the likelihood of adverse events associated with mutagenesis [228,229].

Prolonged presence of vector DNA in reproductive organs may raise concerns regarding the potential transmission of vector DNA to offspring, adding to the list of potential factors limiting the registration of rAAV-based products [230]. An essential aspect of preclinical research in gene therapy involves evaluating how vector DNA and transgene expression (in mRNA or protein form) are distributed among various tissues. This assessment typically includes analyzing blood samples, vital organs, and reproductive organs to monitor the presence and persistence of vector DNA and transgene expression. Additionally, preclinical studies often investigate whether the vector is excreted in biofluids like semen, urine, feces, saliva, and tears.

In studies investigating the safety of rAAV administration for gene therapy in hemophilia B, researchers assessed the potential for transmitting vector sequences to the germline after systemic delivery [231]. Using mouse, rat, rabbit, and dog models, they administered rAAV through intramuscular and hepatic artery routes. In mouse and rat experiments, higher doses of the vector correlated with an increased likelihood of detecting vector sequences in gonadal DNA. In a separate rat study involving intramyocardial injection of 4 × 10^11^ particles of AAV-2-*LacZ*, significant transgene expression was observed in hepatocytes, renal cells, and testicular tissue, with sustained expression noted in the cellular components of seminiferous tubules, including Sertoli cells and spermatogonia-like cells, up to 6 months post-administration [232]. However, no vector sequences were found in dog semen. In rabbits, vector presence in gonadal tissue decreased over time, and fluorescent in situ hybridization indicated localization of AAV signals to the testis basement membrane and interstitial space, without intracellular signals. Clinical studies involving humans who received intramuscular AAV injections up to doses of 2 × 10^12^ gc/kg showed no evidence of vector sequences in semen [233]. Overall, the risk of transmitting vector sequences to germ cells following intramuscular or hepatic artery administration of rAAV was found to be extremely low, which is critical for evaluating the safety of rAAV-based gene therapy. Another study involving rabbits assessed the transmission risk of genetic material to future generations using rAAV at doses ranging from 1 × 10^11^ to 1 × 10^13^ gc/kg. In all cases, semen samples from the rabbits contained vector sequences, with clearance rates depending on dose and time but unaffected by the AAV type. Notably, AAV-8 sequences were found in semen from vasectomized rabbits, indicating that both the reproductive and urinary systems play roles in releasing vectors into semen. The authors propose that host-specific factors partly regulate AAV dissemination into semen [234].

The culmination of the studies underscores that the likelihood of transmitting vector sequences to the germline following administration of recombinant adeno-associated virus via intravenous or intramuscular routes is extremely low. This conclusion is based on findings across various animal models, including mice, rats, rabbits, dogs, and human subjects, consistently showing no detectable vector sequences in seminal fluids or other reproductive tissues post rAAV administration.

**Table 3:** Side effects of rAAV gene therapy based on route of administration.

ROA	rAAV-Linked Side Effects	ROA-Linked Side Effects
IP	Off-target adverse effects (vector diffusion and axonal transport) [147]	Increased brain tissue extracellular fluid with potential buildup and tissue displacement over time; higher agent concentration can enhance molecular distribution, and anatomical precision is necessary; backflow along the injection device; CNS injury or hemorrhagic complications; diminished transduction extent; general anesthesia required [235,236]
IV	High immunogenicity risk; off-target transduction [201]	High-dose vectors required; transfer across the BBB required [11]
IT	DRG toxicity [177]	Extradural CSF leak; controlling vector quantity within the intradural space is difficult; infection; epidural hematoma; subarachnoid bleeding; targets limited to primarily spinal cord and dorsal root ganglia (large dose volume to target the brain) [206]
ICV	-	Parenchymal bleeding; Intraventricular bleeding; general anesthesia required; infection; CSF leak; backflow of drug; transduces primary ependymal cells in the choroid plexus [237]
ICM	-	Bulbomedullary junction injury; general anesthesia required; parenchymal bleeding; intraventricular bleeding; infection; CSF leak; backflow of drug [237]

## 6. Role of PK/PD in Gene Therapy Optimization Using rAAV Vectors: A Clinical Trials Perspective

Gene therapy using rAAVs has become an important treatment strategy for many genetic diseases [104,105,107,238,239,240]. However, the effective and safe use of rAAVs in gene therapy requires in-depth knowledge of the pharmacokinetics (PK) and pharmacodynamics (PD) of the vectors. This section will discuss the relevance of PK/PD studies of rAAV vectors in the context of ongoing clinical trials, noting the variety of serotypes, doses, and routes of administration used to treat various diseases (Table 2). Issues regarding the impact of the vector serotype, the route of administration used, the formulation of the gene preparation, and the personal characteristics of the patient on biodistribution processes, among others, which may contribute to the further development of effective and safe therapeutic strategies, will be summarized.

Conventional descriptions of pharmacokinetics (PK) refer to the processes of absorption, distribution, metabolism, and elimination (ADME) [241]. The concept of conventional pharmacokinetics is valid for drugs introduced into the patient’s body in pharmacologically active form. When considering the pharmacokinetics of products based on rAAV carriers compared to traditional pharmacotherapy, the “in vivo synthesis” step of the product exhibiting therapeutic activity should be taken into account. Section 2 highlights the complexity and hierarchical nature of the processes that ultimately make up the expression of a therapeutic transgene (protein). The sequence of events occurring after the introduction of rAAV vectors into the patient’s body includes vector distribution, binding to target receptors, vector internalization, cytoplasmic transport, escape from the endosome or lysosome, vector decapsidation in the vicinity of the cell nucleus, transport across the karyolemma, formation of an episomal form of DNA, transcription of the transgene and, finally, translation into a therapeutic protein. Therefore, the pharmacokinetics or biodistribution (BD) of rAAV as a drug product can be assessed by analyzing the distribution of both rAAV capsids, the transgene carried, and the protein generated (at the transcriptional and translational levels). The evaluation of vector pharmacokinetics should take into account the phases of distribution, persistence, and clearance at the site of administration and target tissues, as well as nontarget tissues such as liver and biological fluids such as blood, cerebrospinal fluid, and lymph [230,242]. According to EMA requirements for tracking potential vector transmission to third parties, the analysis should also include urine, sweat, saliva, and other physiological fluids that enable virus transmission [242].

By defining the concept of pharmacokinetics, which takes into account the intracellular processes leading to therapeutic effects, it is possible to delineate potential factors influencing rAAV biodistribution. These factors include the serotype-dependent tropism of AAV to specific organs/tissues/cells, regulatory elements controlling DNA transcription, the immunogenic potential of rAAV and the associated purity and method of obtaining the product, the formulation and stability of gene products, and the route of administration (ROA) and the dose.

### 6.1. Serotype-Dependent Factors

The first indispensable step in cell transduction by rAAV vectors is the interaction between the vector capsid and cellular receptors, primary receptors, and coreceptors. Known receptors for rAAV vector serotypes are indicated in Section 2. Serotypic variation in binding to receptors and coreceptors may affect systemic pharmacokinetics. Varying expression levels of individual receptors and coreceptors in tissues and organs [88] and perhaps the Target-Mediated Drug Disposition (TMDD) phenomenon will be determining factors. Putting aside the incompleteness of studies on receptor/coreceptor distribution in tissues of different species, a factor that hinders the biodistribution analysis of rAAV vectors is the lack of knowledge of the receptors for some serotypes (i.e., AAV7, AAV10, AAV11, and AAV13) [243]. Without an adequate understanding of the receptor binding mechanism and expression of this receptor in different tissues, predicting the biodistribution and optimal therapeutic dose of rAAV may be difficult.

The concept of Target-Mediated Drug Disposition was first proposed by Levy in 1994 [244]. It was initially based on observations of the unusual nonlinear pharmacokinetics of several small-molecule drugs. Since then, the concept has been extended to various therapeutic molecules, including gene therapy products [245]. The binding of a drug to a large number of receptors in target tissues can lead to receptor saturation, altering the dynamics of drug distribution and elimination in the body [244]. The TMDD phenomenon, therefore, assumes that the pharmacokinetics of a drug is significantly modified by its receptor binding, usually in target tissues, thus making the pharmacokinetics of the drug dependent on drug-target interactions, which can lead to an unusual, nonlinear dose–effect relationship. In the case of rAAV vectors, the effect is the expression of the transgene, which can be indirectly assessed at the stages of intracellular transgene deposition and mRNA levels and directly by assessing the level of therapeutic protein [230]. For gene therapy products, TMDD can be defined as a nonlinear relationship between the MOI of vectors, intracellular gc levels, and the level of the gene being expressed. An example is the observed differences in transgene insertion efficiency using rAAV2 and rAAVDJ serotypes into mouse brain cells under ex vivo conditions. Differences in the assessed level of viral genome copies (gc) lose statistical significance with increasing vector dose [246]. In addition to the saturation of receptors for rAAV, the efficiency of subsequent stages of cell infection, among others, conversion of genetic material to episomal forms, may play an important role. The stability of viral dsDNA is dependent on host cellular factors [247] and may be related to the rate of rAAV viral capsid rejection, which in turn is serotype-dependent [248]. In summary, the nonlinearity between MOI and transgene levels may be due to the dose- and serotype-dependent saturation of cellular factors by the introduced vector DNA [249].

### 6.2. Formulation Factors

The most well-established method of producing rAAV vectors is transient transfection of human embryonic kidney cells, HEK293, using plasmids. Typically, HEK293 cells are simultaneously transfected with two or three vector plasmids containing the therapeutic gene (GOI), genes encoding Rep/Cap, and adenovirus helper proteins [250]. The Rep/*Cap* sequences and adenovirus helper genes can be placed on one or two separate plasmids [251]. Alternatives for transient transfection include baculovirus expression vectors in Spodoptera frugiperda insect cells (rBV/Sf9) and stably transfected packaging cell lines, producer cell lines (PCLs), due to the simple scalability of culture to large volumes [252,253].

According to EMA requirements for gene therapy medicinal products intended for clinical use, the rAAV batch must meet certain qualitative and quantitative standards concerning product purity and safety criteria [242]. The initial assessment of rAAV vectors’ biodistribution is being evaluated in preclinical studies using animal models. Qualitative–quantitative differences in the composition of gene therapy products for preclinical and clinical studies can generate discrepancies in the pharmacokinetics of vectors in animals and humans. Xin Chen et al. conducted a literature review of only preclinical studies on the biodistribution of gene therapy products, where rAAVs were used as carriers when administered to CSF [254]. The analysis showed significant differences in the biodistribution profiles obtained already at the level of preclinical studies. The authors attribute the reasons to a lack of consistency in methodology between studies, such as the use of different animal models, and a lack of reporting of saturating data, such as the number of copies of the vector, the ratio of empty to full capsids, and the quality of the inserted transgene. Table 4 indicates the most common differences in the evaluation of the rAAV batch intended for preclinical and clinical studies.

The production and purification procedures of rAAV directly affect the quality of the obtained gene therapy drug product and imply the PK/PD course. Analyses of the quality of gene products obtained by methods that differ in the scale of production, among others, are available in the literature [255]. Vectors derived from the same gene construct that were produced using the rBV/Sf9 system showed a higher degree of unresolved and truncated genomes compared to those produced in cells of the HEK293 lineage. Variation was also noted in the number of empty capsids—methods using HEK293 and rBV/Sf9 are associated with 9.1% and 41.1% empty capsids, respectively. The presence of empty AAV capsids can affect the purity and homogeneity of the product, generating batch-to-batch variability, which in turn can affect PK/PD. Deirdre M. O’Connor et al. documented lot-to-lot differences in spinal cord gray matter transduction after intrathecal administration of rAAV9 [256]. The authors did not identify a cause responsible for the described phenomenon but noted the need to develop improved manufacturing and quality control methods to ensure consistency in vector power between batches.

The immunogenic potential of the preparation is one of the factors affecting the efficacy of gene therapy with rAAV vectors. Activation of the recipient’s immune system can significantly affect the assumed rAAV serotype or tissue-specific promoter biodistribution [257]. Besides capsid-specific and viral genome factors, it is important to consider manufacturing-related factors such as product-related impurities and process-related impurities [224,257].

Impurities related to the manufacturing process include those originating from cell substrates (such as host cell proteins and DNA), components used in cell culture (such as inducers, antibiotics, and media), or elements introduced during downstream processing (related to purification processes) [242]. Product-related impurities, including precursors and specific degradation products, are molecular variants that form during manufacturing and/or storage. Although they are structurally similar to the desired product, these impurities lack comparable activity, efficacy, and safety properties [242]. Table 4 outlines both product-related and process-related impurities identified in AAV vector manufacturing.

AAV empty capsids possess a capsid shell that closely resembles that of the desired product, yet they lack the presence of a packaged nucleic acid molecule within. As previously mentioned, the ratio of empty to full capsids depends on the rAAV vector production method and can be as high as 42% [255]. A high ratio of empty to full capsids in clinical products can cause unwanted immunologic reactions and reduce transduction efficiency. Empty capsids can exacerbate adaptive immune responses to the viral capsid antigen and compete for vector binding sites, further decreasing transduction efficiency [220,258]. The guidelines regarding the ratio of empty to full capsids have been included in the regulatory product specifications for virus-based gene therapies [242].

Capable of replicating with the assistance of a helper virus, rcAAV consists of an AAV capsid particle housing AAV *Rep* and *Cap* genes flanked by *ITRs*. rcAAV present in the gene therapy product constitutes product-related impurities [259]. One of the distinguishing features of rAAV vectors in human gene therapy is their non-pathogenicity. Additionally, both wtAAV and rcAAV require the presence of helper viruses, such as adenovirus (Ad) or herpes simplex virus (HSV), for their replication cycle [21]. Assuming a patient previously subjected to gene therapy with a contaminated rAAV product is exposed to these helper viruses, symptoms are more likely to be associated with exposure to pathogenic Ad or HSV. However, it is conceivable that the presence of rcAAV could amplify the immune response against vector-altered cells. Furthermore, in accordance with EMA requirements, it is essential to monitor the potential transmission of vectors to third parties, particularly focusing on replication-competent vectors or oncolytic viruses [242].

Aggregates and other size variants of rAAV pose a potential factor in increasing the immunogenicity of gene therapy products following clinical administration. Like other biological entities, rAAV has the capacity to produce various size variants and aggregated structures [260]. Product-based gene therapy applications in humans should be evaluated for vector aggregates (Table 4).

A prevalent impurity found in various viral preparations is residual nucleic acid, such as cell-substrate DNA from the producer cell line or helper DNA sequences, which may co-purify with the vector [261,262]. The World Health Organization (WHO) has established a threshold for DNA contamination in recombinant protein products as a precaution against oncogene transfer. This DNA limit, set at <10 ng/dose, is widely endorsed within the biotechnology sector [263]. The choice of cell origin for rAAV generation, whether human or non-human, introduces two theoretical risks: genotoxicity and immunotoxicity. Using human cell lines for AAV manufacturing may lead to residual human genomic DNA within the AAV vector product, posing a higher risk of genotoxicity [264]. In contrast, employing insect cells may decrease this genotoxic risk but elevate the risk of immunotoxicity [265].

Host cell proteins, considered to be process-related impurities, present primary theoretical risks associated with immunogenicity concerns. However, these risks are expected to be lower in gene therapy applications compared to therapeutic proteins because gene therapy typically involves a single administration [266].

In conclusion, while the potential for rAAV-based gene transfer to address human disease is significant, our understanding of the quality attributes associated with the vector product and their implications for safety, efficacy, and the immune response in humans remains limited. Formulation factors, such as the method of production and purification of the gene therapy medicinal product, significantly influence the quality attributes of the final product, which directly impact the PK/PD of rAAV.

The pharmaceutical form of drug formulation is also worth analyzing. The most common forms used in clinical trials are infusion and injection. Jenny A Greig et al. evaluated the effect of the infusion rate of intravenous AAV8 vectors in cynomolgus macaques on transgene expression, vector clearance, and the potential to activate the innate immune system [267]. Mean vector levels in the blood, measured 1 min after the end of infusion, were inversely proportional to infusion time. The half-lives of rAAVs administered by intravenous injection and during a 10 min infusion were comparable (~0.6 h). Increasing the infusion time to 90 min generated an almost 10-fold increase in the half-life of rAAV in the blood (~5 h). The authors documented the effect of the form of the gene-drug and the duration of the procedure on the pharmacokinetic parameter, which is the half-life of the drug. It seems likely that administration of the same dose of rAAV to the CNS using different infusion rates can generate a similar relationship.

### 6.3. Route of Application (ROD)

Gene therapy products are administered topically or systemically (Table 2). Administration of the drug directly into the target tissue is aimed at narrowing the target sites for rAAV vectors, i.e., limiting biodistribution. In clinical practice, local administration of selected brain regions has been successfully used [240]. This form of administration allows for high vector titers at the site of action while minimizing exposure of nontargeted tissues to the gene product, reducing the risk of side effects. In addition, local application can reduce the potential negative impact of the immune response on vector biodistribution and therapy efficacy [158,159]. The course of rAAV pharmacokinetics will be different for different routes of drug administration. David R Compton et al. evaluated IP and intra CSF administration using Eladocagene Exuparvovec (Upstaza) [268]. The results showed that all routes of administration induced comparable levels of the transgene in cerebrospinal fluid (CSF). IP administration showed the highest levels of transgene mRNA expression in the putamen, corpus callosum, and caudate nucleus, while expression levels in the spinal cord and dorsal root ganglia (DRG) were undetectable. The ICV/IT routes generated the highest levels of the transgene in the spinal cord and DRG, and low levels in the putamen, corpus callosum, and caudate nucleus. Unlike ICV/IT, the IP route did not result in transgene expression in the blood, suggesting less likelihood of biodistribution to off-target sites. Evaluation of anti-AAV antibody levels identified the IP route as the least immunogenic one.

Systemic drug administration is associated with high patient acceptance, low cost (e.g., compared to stereotactic IP administration), and low risk of developing complications. IV administration for CNS therapy requires consideration of factors limiting the biodistribution of the drug at the site of action. The presence of the BBB is an important factor limiting access to the brain parenchyma, requiring the use of a carrier that effectively penetrates the BBB. Studies of the pharmacokinetics of various rAAV serotypes conducted in a mouse model using tail vein administration revealed significant differences in the profiles analyzed [269]. It is noticeable that the differences in pharmacokinetics probably correlate with the receptor-dependent mechanism of action of rAAV and with clearance from the blood. Serum rAAV9 titers are 1–2 orders of magnitude higher 24 h after administration relative to other vectors [270]. Serum-dependent differences in the clearance of rAAV vectors are also reported in studies using other laboratory animals [271].

### 6.4. Dose

Information on biodistribution is collected during preclinical studies. Based on these, the adequate dose approved for clinical trials is estimated. As previously mentioned, the gene therapy product for use in humans should be analogous to that used in preclinical studies. The serotype, formulation, and manufacturing process with consideration of factors determining the quality, purity, and safety of the product are important. Also, the route of vector administration tested in clinical trials should be analogous to that analyzed in the animal model. A review of clinical trials using rAAV in CNS gene therapy provides information on the vector dose used. It is worth noting that it is characterized by a wide range—5.0 × 10^9^ to 3.0 × 10^15^ gc (Table 2). Leaving aside the need to determine the toxic dose, it is worth remembering the previously described phenomenon of TMDD which has a direct impact on the PK/PD of rAAV-based drug products. Analyzing the PK/PD of AAVs assuming that the vector is not considered as a unity (capsid and transgene separately are subject to intracellular regulation) this saturable cell transduction step by rAAV affects the process of pharmacokinetics.

### 6.5. Patient-Dependent Factors

Typically, preclinical studies evaluate the biodistribution of a vector using a wild-type animal model. The essence of clinical studies, on the other hand, is to evaluate the efficiency, indirectly the distribution of gene preparations in patients with a specific disease. Some studies are evaluating the impact of diseases on transduction efficiency. Paula García-Olloqui et al. found that myocardial infarction significantly increases the transcriptional activity of rAAV8 genomes [272]. Yong Hong Chen et al. demonstrated the effect of a lysosomal-storage-disease-specific increase in CNS sialic acid expression on CNS transduction efficiency in a mouse model of MPSIII [273].

When designing a preclinical study, it is good practice to include individuals of both sexes in the animal model used. Matthew Piechnik et al. investigated sex-dependent differences in the immune response to AAV gene therapy in mice with mucopolysaccharidosis IVA (MPS IVA) [274]. Cardiac histology revealed a lack of normalization of vacuolization in females, in contrast to complete recovery in male mice. Casey A Maguire et al. showed that intravenous injection of AAV9 induces higher transgene expression in the brain of females compared to male mice [275]. This observation was consistent across two strains of mice (nude and C57BL/6) and correlated with a higher number of AAV genomes in the brains of female mice.

In contrast, Albert M Maguire et al. evaluated visual function in 12 patients (aged 8–44 years) with Leber syndrome who received a single subretinal injection of the rAAV2-*hRPE65* virus [276]. The patients showed a 2-logarithmic increase in pupillary response to light. The results of the study highlighted an 8-year-old patient whose level of light sensitivity was comparable to that of healthy peers. The greatest improvement in eye-motor coordination was seen in the youngest patients. The authors pointed out the dependence of PD rAAV on patient age.

Possible modifications of the rAAV vector in the time between administration into the patient’s body and the achievement of the molecular target should also be considered. Compared to pharmacokinetic considerations of small-molecule drugs, the possibility that viral particles interact with blood components seems most intuitive. Wang et al. showed that human serum albumin (HSA) directly interacts with the AAV capsid and increases transduction efficiency [277]. Mechanism studies indicate that HSA increases AAV binding to target cells and that the interaction of HSA with AAV does not affect the AAV infection pathway. Acute phase protein CRP, Serum albumin, LDL, and transferrin are also indicated as factors that modify the transduction potential of rAAV vectors [278,279].

### 6.6. Drug–Drug Interaction

In the realm of AAV studies, significant attention is directed towards strategies aimed at augmenting AAV vector transduction efficiency. Some of these approaches involve modifying cellular physiology using pharmacological agents. Numerous pharmacological agents have been employed to enhance AAV transduction across different infection stages, thereby implying potential interactions between pharmacotherapeutic and gene therapy agents. Traditionally (referring to classical pharmacological products), interactions are distinguished into pharmacological and pharmacokinetic phases. Pharmacodynamic interactions arise when drugs compete at the target site or exhibit similar/opposing therapeutic or adverse effects. Pharmacokinetic interactions primarily influence drug concentration within the body and can occur during any phase of the LADME process (liberation, absorption, distribution, metabolism, excretion). However, the conventional PK/PD framework may not directly apply to gene therapy products.

Complementary analytical experiments have demonstrated that human serum albumin (HSA) interacts directly with the rAAV capsid, thereby promoting rAAV transduction. Mechanistic studies indicate that HSA facilitates rAAV binding to target cells without interrupting the rAAV infection pathway. Moreover, this interaction with albumin does not affect the efficacy of neutralizing antibodies [277]. In the context of vector targeting hepatocytes, Xiaolei Pei et al. have shown competition for binding sites on the AAV8 virion surface among HSA, LDL, and transferrin [279]. Human serum albumin, the most abundant protein in serum, is a monomeric, multi-domain macromolecule. HSA exhibits remarkable ligand-binding capacity, serving as a reservoir and carrier for numerous endogenous and exogenous compounds, including drugs [280]. There is a wide range of pharmacological drugs that exhibit interactions associated with their degree of binding to plasma proteins, including albumin. These drugs often have acidic properties, such as warfarin [281]. It seems likely that different effects of gene therapy may occur in patients concurrently using drugs that exhibit strong affinity for proteins, simultaneously displacing other particles from these interactions.

The literature reveals many indications of how pharmacological agents impact the efficacy of gene therapy protocols conducted in vivo. In the CUPID trial, patients treated with high doses of AAV1/*SERCA2a* exhibited the most pronounced improvement in clinical heart failure parameters compared to placebo. It is noteworthy that these patients received the lowest concomitant doses of beta blockers [282]. A study in a large animal model (pigs) demonstrated that nitroglycerin enhances the efficiency of cardiomyocyte transduction by viral vectors (Ad-*βgal*, AAV-*βgal*) [283]. The simultaneous intravenous administration of nitroglycerin with coronary administration of AAV1/*SERCA2a* has been demonstrated to improve gene transfer in pig hearts.

Several studies have documented increased efficiency in cell transduction by rAAV vectors in vitro when pharmacological agents, particularly anticancer drugs, are present. These may not fit the traditional definition of interactions, but presenting them as such seems to be an accepted approach, as evaluating the pharmacokinetics of rAAV involves analyzing both the capsids, the carried transgene, and the resulting protein expression. As early as 1995, it was observed that DNA-damaging agents such as aphidicolin, hydroxyurea, etoposide, and camptothecin increased the transduction of nondividing cells [284]. A likely factor responsible for enhanced cell transduction by rAAV vectors in the presence of drugs, which could trigger similar DNA repair functions, is the mobilization of host DNA polymerases. These enzymes are necessary for converting single-stranded vector genomes into double-stranded molecules. Several years later, Konkal-Matt R Prasad et al. demonstrated the impact of topoisomerase inhibitors on accelerating AAV2-mediated gene expression in the mouse heart [285].

In 2000, Dongsheng Duan et al. applied tripeptide proteasome inhibitors to enhance rAAV gene delivery to differentiated human airway epithelia from their apical surfaces, despite the absence of known AAV2 receptors or coreceptors [286]. In the airway, the primary rate-limiting steps in rAAV transduction from the mucosal surface appear to involve inefficient endosomal processing of the internalized virus. The proteasome system plays a role in regulating endocytosis, hence the application of a proteasome inhibitor stimulated viral trafficking to the nucleus [287,288].

A 2009 combination therapy study analyzed the effects of concomitant chemotherapy and reovirus on B16-F10 cancer cells in vitro and in a mouse model. The combination of cisplatin and reovirus exposure led to an increased population of cells undergoing late apoptosis/necrosis. Cisplatin nearly completely abolished the reovirus-induced overexpression of pro-inflammatory cytokine genes. This combination therapy significantly delayed tumor growth and improved survival in vivo [289].

In 2013, Angela M. Mitchell et al. presented data supporting a model in which As_2_O_3_ (FDA-approved chemotherapeutic agent) increases rAAV transduction both in vitro and in vivo. As_2_O_3_ also maintains perinuclear accumulations of capsids, thereby facilitating productive nuclear trafficking [290].

In 2016, high-throughput screening identified small molecules that enhance cell transduction with rAAV vectors. These molecules were classified into five groups: (1) Topoisomerase II inhibitors, such as Etoposide and Teniposide; (2) DNA strand damage-generating agents, for example, Bleomycin; (3) DNA epigenetic change-inducing compounds, including Nanaomycin A and Vorinostat; (4) Anthracyclines, which exert various effects including inhibition of topoisomerase and proteasome activity; and (5) Non-anthracycline compounds that inhibit the proteasome, such as Bortezomib. The authors emphasize that these findings show that certain categories of medications improve adeno-associated virus gene delivery through mechanisms that are presently unknown [291].

However, the likelihood of a patient concurrently taking cytotoxic drugs and a gene therapy product is minimal. Attention should be given to immunosuppressive drugs, which are widely used in patients undergoing gene therapy. Glucocorticoids, known for their anti-inflammatory and immunosuppressive properties, are frequently used to prevent liver toxicity following the systemic administration of high doses of AAV vectors in gene therapy [218]. Zheng Chai et al. conducted an elegant study showing that dexamethasone transiently enhances transgene expression in the liver after adeno-associated virus transduction [292]. The authors showed that prior administration of dexamethasone as well as post rAAV administration increases the expression of therapeutic protein. The authors propose that the transiently increased transgene expression may be related to the immunosuppressive function of dexamethasone. Dexamethasone administration did not alter AAV genome copy number or transgene expression at the transcriptional level, but did transiently reduce interferon beta (IFN-β) and tumor necrosis factor alpha (TNF-α) expression in mouse livers.

The impact of glucocorticosteroids on the intracellular distribution or stabilization of viral DNA, rather than on quantitative changes in the AAV genome, is worth considering. The steroid-mediated gene delivery strategy involves using glucocorticosteroid receptors (GR) for gene delivery. In the studies, a dexamethasone derivative was used to deliver reporter plasmids. Increased transgene expression was documented in dividing cells, as well as enhanced nuclear accumulation of the transgene in GR-positive cells [293].

In vitro studies also demonstrate an increase in transduction efficiency when dexamethasone is present. The efficiency of transfection of human mesenchymal stem cells (hMSCs) using a nonviral vector (Lipofectamine) is enhanced in the presence of glucocorticoids. Glucocorticoids significantly improve transfection in hMSCs, showing a 3-fold increase in efficiency and a 4–15-fold increase in transgene expression. These effects are attributed to the binding of glucocorticoid receptors (GR), which preserve regular metabolic activity and augment both cellular (5-fold) and nuclear (6–10-fold) DNA uptake in hMSCs compared to transfection without glucocorticoids [294]. Recent findings indicate that glucocorticoids increase transgene expression through the transcriptional activation of endogenous hMSC genes by the cytoplasmic glucocorticoid receptor [295]. Activation of GR modifies the expression of endogenous hMSC genes, mitigating endoplasmic reticulum stress, oxidative stress, apoptosis, and inflammatory responses induced by transfection. This protective mechanism helps maintain hMSC metabolism and protein synthesis, ultimately enhancing transgene expression after nonviral gene delivery to hMSCs. Numerous publications have detailed the complex signal transduction pathways and unique gene activation patterns induced by glucocorticoids and related drugs [296,297,298].

**Figure 5:**
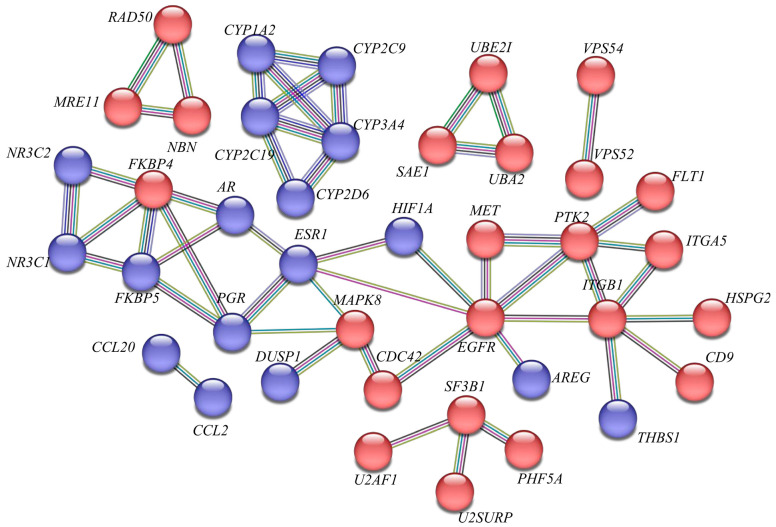
Correlation analysis between rAAV transmission genes (red nodes) and drug-modified genes (blue) using a String database [304]. The hit score constitutes the combined score > 0.9, which is computed by combining the probabilities from the different evidence channels and correcting for the probability of randomly observing an interaction.

Considerable effort is focused on understanding the AAV life cycle and the mechanisms governing transduction and transgene expression. This includes identifying host factors that may limit the efficiency of rAAV transduction [299]. In a genome-wide RNAi screening conducted by Mano et al., a plethora of genes influencing rAAV vector transduction efficiency were unveiled [300]. Among these host restriction factors implicated in rAAV transmission are *SETD8*, *CASP8A2*, *SOX15*, *TROAP*, *PAT*, *PHC3*, *HAF1A*, *SF3B*, *RTBDN*, and *WWC2*. Furthermore, as evidenced in our recent investigation, microRNAs (miRNAs) also exert a significant impact on transduction efficiency [301]. The studies mentioned suggest that the efficiency of rAAV transduction may also be influenced by conventional drugs commonly used in human therapy. Figure 5 depicts a considerable degree of interaction between genes associated with rAAV transmissibility (depicted as red nodes) and genes modified by dexamethasone (depicted as blue nodes, DSigDB [298]). These interactions may have implications for the efficiency of cell transduction by AAV. Modulation of gene expression levels of “host factors” by pharmacotherapeutics such as dexamethasone should be considered as a potential mechanism responsible for modifying the efficiency of cell transduction by rAAV vectors. Simultaneously, this factor indirectly affects the pharmacokinetics/pharmacodynamics of rAAV. It is noteworthy that treatment with dexamethasone (DEX) increases cytosolic levels of HIF-1alpha [302]. According to the STRING Database, there is co-expression between *HIF1A* and *EGFR*, which acts as a co-receptor for AAV6 [303,304]. This raises concerns about potential drug interactions when dexamethasone and a gene therapy product based on rAAV6 are administered concurrently.

### 6.7. Food–Drug Interaction

A drug–food interaction is a phenomenon whereby components of food influence the pharmacokinetics or pharmacodynamics of a drug. Drug–food interactions are a significant aspect of pharmacotherapy, which can substantially impact the efficacy and safety of treatment. Understanding and managing these interactions is crucial for optimizing patient health outcomes. Data regarding the influence of ingested food on the efficacy of intravenously administered viral vector-based gene therapy are relatively limited. Despite the growing interest in gene therapy, detailed investigations concerning the interactions between food and gene therapy are scarce.

Presently, most studies focus on assessing the efficacy, safety, and potential adverse effects of gene therapy, while the impact of food on these aspects may not be directly addressed. Nevertheless, there is some evidence suggesting that certain dietary components, such as fatty acids, antioxidants, or plant compounds, may affect the immune response and metabolic processes, theoretically influencing the effectiveness of gene therapy. The potential impact of ingested food on the effectiveness and efficiency of viral vector-based gene therapy could be multifaceted. Dietary components can potentially influence various aspects of the pharmacokinetics and pharmacodynamics of gene therapy.

Viruses, including rAAV, utilize membrane rafts for attachment to the cell surface, as well as for employing endocytic and non-endocytic mechanisms to enter host cells [78,305]. Membrane rafts are small (10–200 nm), heterogenous, highly dynamic, sterol- and sphingolipid-enriched domains that compartmentalize cellular processes. Small rafts can sometimes be stabilized to form larger platforms through protein–protein and protein–lipid interactions [306]. Overconsumption of long-chain polyunsaturated fatty acids over a period of 7 days resulted in alterations to the primary lipid components of hepatocytes membranes. This included an elevation in total unsaturated fatty acids with long chains, incorporated into acyl-carnitine or diacylglycerol components, alongside a reduction in total short-chain acyl-carnitines, glycerophosphocholines, lysophosphatidylcholines, and sphingolipids associated with the membrane [307]. Dietary fats could influence the circulation time or distribution of the viral vectors in the bloodstream, potentially affecting their delivery to target tissues by altering the composition of membrane rafts used for attachment to the cell surface. Similarly, modification of membrane raft composition can be induced by consuming a diet rich in omega-3 fatty acids [308]. Dietary omega-3 fatty acids have a direct effect on the metabolism of arachidonic acid (a component of raft membranes) by displacing it from cellular membranes [309].

In the aforementioned study regarding the influence of serum proteins, a positive effect of LDL on hepatocyte transduction efficiency was demonstrated [279]. Elevated consumption of industrial trans fatty acids (iTFA) leads to an increase in circulating low-density lipoprotein cholesterol, as is well known [296]. Changes in LDL levels in the blood of patients eligible for treatment with rAAV-based products may potentially impact vector pharmacokinetics/pharmacodynamics.

Another potential factor influencing vector biodistribution is the immune response, which can be modulated indirectly through dietary components. The adverse effects associated with the patient’s immune response to rAAV vectors are documented in the literature [19,21,201,220,310]. Numerous dietary components, such as vitamins, minerals, probiotics, and prebiotics, exhibit immunomodulatory properties. Nutrients play a crucial role in innate immunity and inflammation by regulating Toll-like receptors (TLRs) expression, as well as pro- and anti-inflammatory cytokines, thus modulating immune cell communication and signaling pathways. Nutrients also contribute to adaptive immune responses by influencing B and T lymphocyte differentiation, proliferation, activation, and antibody production. Microbiota signaling affects cytokine expression, antiviral mediator production, and DNA modifications in immune cells, including macrophages, effector T helper cells, and regulatory T cells [311]. Certain dietary components, like fiber and fats, can alter gut microbiota composition and inflammatory marker levels such as C-reactive protein and interleukin-6 [312]. The inaugural investigation concerning the influence of serum proteins on AAV vector transduction efficacy indicated that systemic transduction of mice unequivocally exhibited a substantial enhancement in the transduction efficiency of rAAV vectors 1 and 6 within skeletal muscles by over 10-fold, attributed to the binding with mouse CRP (mCRP). The phenomenon is restricted in a serotype- and species-specific manner. Human CRP does not interact with either rAAV-1 or rAAV-6. Therefore, the robust efficiency of muscle transduction mediated by mCRP with these serotypes in mice cannot be extrapolated to humans [278].

In studies on vitamin influence, carotenoids were found to regulate immune-related gene expression, including Toll-like receptors (TLRs), heat shock proteins (HSP70 and HSP90), thioredoxin-like protein (TRX), and peptidoglycan recognition receptor proteins (PGRPs) [313]. Vitamin D, in turn, modulates T helper cell responses, showing strong anti-inflammatory effects by suppressing Th1-mediated immune responses and reducing the expression of proinflammatory cytokines like interleukin-2, interferon-gamma, interleukin-6, tumor necrosis factor-alpha, and interleukin-17 in Th cells [314].

Trace elements, essential micronutrients present in small quantities within organisms, play vital biological roles as cofactors of numerous enzymes and antioxidant molecules. For example, selenium enhances cellular immunity by promoting T cell receptor-induced T cell activation and Th0 differentiation into Th1 cells in mice [315].

Drug–food interactions are an important aspect of pharmacotherapy that can significantly impact the effectiveness and safety of treatment. Understanding and managing these interactions is crucial for optimizing patient health outcomes. Additionally, it should be noted that they may also be a factor to consider in optimizing gene therapy treatments, although as of now, there are no studies demonstrating a direct impact of diet on the effectiveness and safety of gene therapy.

## 7. Conclusions and Future Directions

With the rapid advancement of rAAV-based gene therapy and the ongoing progress in our comprehension of nervous system biology, numerous promising avenues for future research and clinical applications emerge. Treatment of a disease with gene therapy products involves identifying the causative gene, formulating an appropriate therapeutic sequence, selecting a suitable vector, and devising an efficient method of administering the genetic material (ROA), while also determining the optimal dosage, taking into account the pharmacokinetics/pharmacodynamics (PK/PD) of therapeutic particles. Various routes of administration, including intraparenchymal, intracerebroventricular, intracisternal, intrathecal, and intravenous, are employed in clinical trials aimed at treating CNS diseases with gene preparations based on rAAV vectors. Further efforts in developing minimally invasive, effective, safe, and targeted gene transfer methods are necessary. Intravenous administration deserves special attention due to its low risk of complications, high patient acceptance, and the lack of necessity for specialized medical instrumentation or patient sedation/anesthesia.

Numerous efforts in AAV capsid engineering have focused on discovering new capsids that not only evade the host immune system but also enhance CNS delivery. These efforts have utilized methods such as rational design, directed evolution, and in silico design. AAV is extensively used as a delivery vector for in vivo gene therapy, as evidenced by hundreds of clinical trials and six approved drugs, including two targeting CNS diseases [103,104,106,316]. Its relatively low immunogenicity and toxicity, sustained efficacy, and broad tropism make AAV a promising candidate for treating various conditions, particularly those affecting the CNS. However, the efficiency of delivery to the CNS due to the BBB, remains a significant challenge for the broader clinical application of AAV gene therapy [10]. Therefore, there is a pressing need for AAV engineering to develop next-generation capsids with enhanced properties, such as improved BBB penetration, reduced immunogenicity, and greater packaging efficiency. Examples of efforts on improved BBB penetration include AAV-PHP⋅B and AAV-PHP.eB vectors, which exhibit the highest efficiency in transducing the CNS, especially in C57Bl/6 mice [17,174]. Unfortunately, this is due to the strain-specific expression of the Ly6a receptor in these mice [114]. Other examples of rAAV engineering vectors include AAV-AS, AAV-F, and AAV-B1 [110,117,121]. All of these are more efficient in transducing the CNS than the parental rAAV9 vector, with increases ranging from 6- to even 171-fold higher. An interesting strategy for enhancing efficacy in crossing the blood–brain barrier involved employing a rational design approach to incorporate BBB shuttle peptides (such as Bax-inhibiting peptides) that interact with AAV. Additionally, cell-penetrating peptides (CPPs) were utilized, which are short peptides capable of traversing biological membranes and facilitating the cellular uptake of otherwise membrane-impermeable molecular cargoes [317]. This combined approach resulted in increased BBB transcytosis and subsequent CNS transduction. In the cited study, transduction efficiency increased 5-fold in cynomolgus macaques compared to the AAV9 parent vector.

Despite promising results obtained with BBB-crossing vectors such as rAAV9, its variants and rAAVrh.10, further research on their efficacy and safety is needed. Studies on the mechanisms of crossing the BBB are particularly important, which will enable the development of an efficient and reproducible delivery system for transgenes to the CNS. Considering interspecies differences, the challenge lies in extrapolating proof-of-concept studies conducted using animal models to humans. Studies of rAAV vector biodistribution conducted on small animal models require careful translation of the results to larger organisms. It seems justified to expand research methodologies, certainly to include additional species, but studies on biodistribution should also consider various strains of laboratory animals. Such an approach will help avoid unintended categorization of rAAV vector variants as BBB crossing, which may exhibit limited neurotropism to a specific species or even strain.

Simultaneously, the immunotoxicity associated with intravenous administration of high doses of rAAV must also be considered [201]. The activation of the patient’s immune system by rAAV gene therapy depends on several factors, including the route of administration, dosage, vector serotype, carried transgene, and other components of the vector DNA. Previous exposure to the virus is also crucial, as it results in elevated anti-AAV antibody titers, which can disqualify (typically > 1:50) a patient from receiving rAAV-based treatment. While intravenous administration of AAV vectors is characterized by high clinical utility and has shown remarkable success in treating spinal muscular atrophy, fatalities have occurred due to liver, kidney, heart, or lung failure [201]. Local administration methods, such as intra-CSF and intraparenchymal, are characterized by a lower risk of treatment-emergent serious adverse events, including those associated with overactivation of the patient’s innate and adaptive immune system [159,318]. Nonetheless, immune responses to the vector significantly influence its toxicity. Upon administration of AAV vectors, the innate immune system can detect foreign viral particles through interactions between the AAV capsid and vector genome with pattern recognition receptors like Toll-like receptors. This recognition can subsequently trigger the expression of major histocompatibility complex genes and the secretion of pro-inflammatory cytokines or interferons [319]. The secreted interferons and cytokines, in turn, induce the expression of genes that restrict viral replication and stimulate adaptive and memory immune responses [320]. In addition, an innate immune response involves the activation of the complement system [321]. The adaptive immune system can respond by generating antibodies or cytotoxic T cells that target AAV capsid and transgene proteins [322]. These antibodies can neutralize AAV vectors, preventing them from effectively delivering therapeutic genes to target cells. AAV-specific T cells can identify and destroy AAV-infected cells, involving both CD8+ cytotoxic T cells and CD4+ helper T cells. In some cases, a strong T-cell response may lead to the elimination of AAV-transduced cells [323].

Therefore, strategies to modulate and mitigate immune responses upon vector administration should address both innate and adaptive immune response. Among the strategies applied in clinical settings to address innate immune responses are those aimed at evading the complement system [324]. One strategy involves using the C3 modulator drug APL-9, which inhibits C3 activation and blocks all complement activation pathways. When administered with rAAV, APL-9 effectively controlled complement activity within an hour and maintained this effect for up to 12 h [325]. Another strategy to dampen the innate immune response involves using a monoclonal antibody aimed to block the C5 component of the complement system. The administration of eculizumab to patients with atypical hemolytic uremic syndrome demonstrated an efficacy rate of 80% [326].

In clinical research, various strategies are being explored to mitigate the adaptive immune response. One approach involves the use of immunomodulatory agents, such as corticosteroids or immunosuppressants, to suppress the activity of T cells and reduce inflammatory responses. Excluding patients with pre-existing neutralizing antibodies (NAbs) is employed to minimize immune responses. For example, the trials of the Novartis AAV9-*SMA1* vector (Zolgensma), patients with Anti-AAV9 antibody titers greater than 1:50 were excluded (NCT03306277). Additionally, altering the route of administration of AAV vectors (NCT04133649) and reducing the therapeutic dose are being investigated as means to reduce immune recognition and enhance therapeutic efficacy.

Several intriguing strategies in the preclinical research phase are worth noting, such as the incorporation of TLR9-inhibitory (TLR9i) DNA sequences into the AAV genome, which can reduce innate immune responses in mice and pigs [310]. Another approach involves microRNA-mediated detargeting, which enables tissue and cell-specific transgene expression by suppressing unwanted expression from non-target cells. By diminishing antigen presentation by innate immune cells, this strategy can subsequently impede adaptive immune responses to transgenes delivered by AAVs [327]. The development of neutralizing antibodies (NAbs) poses a challenge to AAV gene therapy, leading to the implementation of various strategies to overcome the humoral immune response. Research has demonstrated encouraging outcomes in reducing NAb titers through B-cell depletion following the administration of rituximab (anti-CD20 antibody) [328]. Nevertheless, systemic immunosuppression may elevate the susceptibility to infection and does not guarantee complete remission of high-titer neutralizing antibodies [329]. The currently registered gene therapy products indicated for the treatment of CNS disorders, such as Zolgensma and Upstaza, involve the administration of a single dose [106,107]. This treatment regimen is based on the low mitotic activity of CNS cells and the low risk of transgene loss [330]. Supporting factors include long-term successes in treating other conditions, such as clinical trials for Leber’s congenital amaurosis, where sustained results have been observed for up to 7.5 years in the full-field light sensitivity threshold test [217]. The potential need for AAV vector re-administration in certain cases, such as children or neonatal patients who will undergo growth and tissue proliferation, as well as patients with degenerative disorders, presents a significant challenge. In light of the above, another noteworthy strategy to mitigate adaptive immune responses is the utilization of an mTOR pathway inhibitor to induce selective immune tolerance to a co-administered biologic drug. Tolerogenic ImmTOR nanoparticles containing rapamycin have been demonstrated to prevent the formation of neutralizing anti-capsid antibodies, thus allowing for vector re-administration [331]. Rapamycin suppresses effector T cell activation and is clinically used in chronic immunosuppressive regimens to prevent renal transplant rejection. Furthermore, adding ImmTOR to AAV gene therapy vectors has recently been shown to effectively and specifically inhibit adaptive antibody and T cell immune responses against the AAV capsid. This allows for successful repeat administration of AAV vectors in mice and nonhuman primates [332].

With the reported presence of antibodies against AAV in humans ranging from 40–70%, there is indeed an urgent need for utilizing AAV with improved properties, such as lower immunogenicity. Capsid engineering provides an alternative method for reduced potential for immune response [333]. The AAV biology within the realm of immunology is subject to investigation. Increasingly, efforts are directed towards mapping antibody epitopes on the capsid [334]. Neutralizing antibodies (NABs) often target conserved residues across different AAV subtypes, particularly those near symmetry axes such as the three-fold axis. Modifying these residues in AAV1 allowed the virion to evade specific antibody subsets, as demonstrated in experiments with pre-immunized mice and non-human primates (NHPs). Furthermore, the combination of multiple mutations led to a cumulative reduction in neutralization [335,336]. Another approach involves chemical modifications of AAV capsids, such as PEGylation and polymer encapsulation [337]. However, these alterations may diminish gene delivery efficiency, decrease production yield, alter the distribution of AAV vectors in the body, and induce the formation of antibodies against the modified capsids [338]. Additionally, it has been demonstrated that AAVs enveloped in exosomes can efficiently deliver genes to target cells, even in the presence of pre-existing immunity, induce tolerance, and modify the targeting abilities of AAVs [339]. Understanding the molecular basis of interactions, knowledge of specific receptors and co-receptors for all serotypes of rAAV vectors are necessary to evaluate rAAV vector biodistribution. An additional possible solution is a detailed explanation of the distribution of receptors and coreceptors among different species. Striving to understand the precise mechanisms of interaction between rAAV vectors and target cells receptors may lead to the development of more precise and effective therapeutic strategies. Additionally, considering the genetic diversity and expression of receptors and co-receptors may help tailor gene therapy to individual patient needs.

Another future direction is the development of minimally invasive methods for delivering AAV vectors to the brain bypassing the BBB, such as intranasal administration. Research on the mentioned ROA is privileged by the direct connection of the site of administration to the CNS. At the same time, local application can reduce the potential risk of adverse events, such as the transduction of unintended tissues, and perhaps reduce the dose of vectors used.

Finally, further research on rAAV vector gene therapy products should include analyses of factors influencing PK/PD, such as serotypic variations, formulation differences, administration routes, and dosages. This will be crucial for developing even more effective and safer gene therapies. Among the formulation factors, we want to draw particular attention to the optimization of production processes. Variations in efficient and homogeneous therapeutic preparations may arise from differences in the quality and purity of rAAV products, which are dependent on the chosen method of vector production. Optimization of production processes may improve pharmacokinetics and, consequently, therapy effectiveness. It is worth noting here the necessity for standardization of qualitative–quantitative properties, including the purity of medicinal products for gene therapy, both in preclinical and clinical studies. The consistent use of products with identical quality and purity across preclinical and clinical studies will generate reliable data on the biodistribution and efficacy of therapy using rAAV vectors. Further research on standardizing methodology and improving the quality of data from preclinical studies may contribute to better predicting the behavior of rAAV vectors in humans. Another factor also considered in traditional pharmacokinetic analysis is the drug-binding coefficient with blood components, mainly proteins. Pharmacokinetics of intravenously administered gene drugs depends on the interaction of therapeutic particles with blood components. Hence, studies on the potential impact of blood components, such as serum albumin or acute-phase proteins, can provide new insights into biodistribution mechanisms and potential factors modifying the effectiveness of gene therapy. In summary, further research on the pharmacokinetics and pharmacodynamics of rAAV vectors is crucial for the continued development of effective and safe gene therapy strategies.

## Figures and Tables

**Table 2:** Clinical trials for CNS disorders involving AAV viral vectors.

Administration Route	Disease	Serotype	Transgene	Dose	NCT Number
IP	AADC	AAV2	*AADC*	1.3 × 10^11^–1.6 × 10^12^ gc	NCT02852213
	AADC	AAV2	*AADC*	1.81 × 10^11^ gc	NCT01395641
	AADC	AAV2	*AADC*	1.81 × 10^11^–2.37 × 10^11^ gc	NCT02926066
	AADC	AAV9	*AADC*	N/A	NCT05765981
	AD	AAV2	*NGF*	2.0 × 10^11^ gc	NCT00876863
	AD	AAV2	*NGF*	1.2 × 10^10^–1.2 × 10^11^ gc	NCT00087789
	AD	AAV2	*BDNF*	N/A	NCT05040217
	CD	AAVOlig001	*ASPA*	3.7 × 10^13^ gc	NCT04833907
	FTD	AAV9	*GRN*	N/A	NCT06064890
	GD2	AAV9	*GBA1*	N/A	NCT06272149
	HD	AAV5	*miHTT*	6.0 × 10^12^–6.0 × 10^13^ gc	NCT04120493
	HD	AAV5	*miHTT*	6.0 × 10^12^–6.0 × 10^13^ gc	NCT05243017
	HD	AAVrh10	*CYP46A1*	4.0 × 10^8^–1.1 × 10^9^ gc/µL	NCT05541627
	LINCL	AAV2	*CLN2*	3.0 × 10^12^ gc	NCT00151216
	LINCL	AAVrh10	*CLN2*	2.85 × 10^11^–9.0 × 10^11^ gc	NCT01414985
	LINCL	AAVrh10	*CLN2*	2.85 × 10^11^–9.0 × 10^11^ gc	NCT01161576
	MLD	AAVrh10	*ARSA*	1.0 × 10^12^–4.0 × 10^12^ gc	NCT01801709
	MPS IIIA	AAVrh10	*SGSH*	N/A	NCT03612869
	MPS IIIA	AAVrH10	*SGSH, SUMF1*	N/A	NCT01474343
	MPS IIIB	AAV5	*NAGLU*	4.0 × 10^12^ gc	NCT03300453
	MSA	AAV2	*GDNF*	N/A	NCT04680065
	NGLY1	AAV9	*NGLY1*	N/A	NCT06199531
	PD	AAV2	*GDNF*	9.0 × 10^10^–3.0 × 10^12^ gc	NCT01621581
	PD	N/A	*N/A*	N/A	NCT05822739
	PD	AAV2	*GAD*	1.0 × 10^12^ gc	NCT00643890
	PD	AAV2	*AADC*	9.0 × 10^10^–3.0 × 10^11^ gc	NCT00229736
	PD	AAV2	*AADC*	3.0 × 10^11^–9.0 × 10^11^ gc	NCT02418598
	PD	AAV2	*GDNF*	N/A	NCT04167540
	PD	AAV2	*NTN*	5.4 × 10^11^ gc	NCT00400634
	PD	AAV2	*NTN*	9.4 × 10^11^–2.4 × 10^12^ gc	NCT00985517
	PD	AAV2	*AADC*	7.5 × 10^11^–4.7 × 10^12^ gc	NCT01973543
	PD	AAV2	*AADC*	9.4 × 10^12^ gc	NCT03065192
	PD	AAV2	*GAD*	1.0 × 10^11^–1.0 × 10^12^ gc	NCT00195143
	PD	AAV2	*AADC*	3.6 × 10^12^ gc	NCT03562494
	PD	AAV2	*GAD*	N/A	NCT05603312
	PD	AAV2	*GDNF*	N/A	NCT06285643
	PD	AAV2	*NTN*	1.3 × 10^11^–5.4 × 10^11^ gc	NCT00252850
	RS	AAV9	*MECP2*	1.0 × 10^15^ gc	NCT05898620
	TLE	AAV9	*miGRIK2*	N/A	NCT06063850
IT	AD	AAVrh10	*APOE2*	1.4 × 10^10^ gc/mL CSF–1.4 × 10^14^ gc	NCT03634007
	ALS	AAVrh10	*miSOD1*	N/A	NCT06100276
	AMN	AAV9	*ABCD1*	N/A	NCT05394064
	CLN3 BD	AAV9	*CLN3*	6.0 × 10^13^–1.2 × 10^14^ gc	NCT03770572
	CLN6 BD	AAV9	*CLN6*	1.5 × 10^13^ gc	NCT02725580
	CLN7 BD	AAV9	*CLN7*	5.0 × 10^14^–1.0 × 10^15^ gc	NCT04737460
	GAN	AAV9	*GAN*	3.5 × 10^13^–3.5 × 10^14^ gc	NCT02362438
	IGHMBP2	AAV9	*IGHMBP2*	N/A	NCT05152823
	GM2	AAV9	*HEXA, HEXB*	N/A	NCT04798235
	RS	AAV9	*miniMECP2*	N/A	NCT06152237
	RS	AAV9	*miniMECP2*	N/A	NCT05606614
	SMA	AAV9	*SMN*	6.0 × 10^13^–2.4 × 10^14^ gc	NCT03381729
	SMA	AAV9	*SMN1*	2.4 × 10^14^–4.8 × 10^14^ gc	NCT05824169
	SMA	AAV9	*SMN1*	1.2 × 10^14^–4.8 × 10^14^ gc	NCT05901987
	SP50	AAV9	*AP4M1*	N/A	NCT05518188
ICM	FTD	AAV9	*GRN*	N/A	NCT04408625
	FTD	AAV1	*GRN*	3.3 × 10^10^–2.2 × 10^11^ gc/g *	NCT04747431
	GD2	AAV9	*GBA1*	N/A	NCT04411654
	GM1	AAVhu68	*GLB1*	3.3 × 10^10^–2.2 × 10^11^ gc/g *	NCT04713475
	GM1	AAVrh10	*GLB1*	8.0 × 10^12^ gc/kg	NCT04273269
	MPS I	AAV9	*IDUA*	1.0 × 10^10^–5.0 × 10^10^ gc/g *	NCT03580083
	PD	AAV9	*GBA1*	N/A	NCT04127578
IV	CD	AAV9	*ASPA*	N/A	NCT04998396
	GM1	AAV9	*GLB1*	1.5 × 10^13^–4.5 × 10^13^ gc/kg	NCT03952637
	KD	AAVrh10	*GALC*	3.0 × 10^13^ gc/kg—N/A	NCT04693598
	KD	AAVrh10	*GALC*	1.6 × 10^13^ gc/kg—N/A	NCT05739643
	MPS IIIA	AAV9	*SGSH*	5.0 × 10^12^–3.0 × 10^13^ gc/kg	NCT02716246
	MPS IIIB	AAV9	*NAGLU*	2.0 × 10^13^–1.0 × 10^14^ gc/kg	NCT03315182
	OTC	AAV8	*OTC*	2.0 × 10^12^–1.0 × 10^13^ gc/kg	NCT02991144
	PKA	AAV5	*PAH*	N/A	NCT04480567
	PKA	AAVSNY001	*PAH*	N/A	NCT05972629
	PKA	AAVHSC15	*PAH*	N/A	NCT03952156
	SMA	AAV9	*SMN1*	1.1 × 10^14^ gc/kg	NCT03461289
	SMA	AAV9	*SMN1*	1.1 × 10^14^ gc/kg	NCT03505099
	SMA	AAV9	*SMN1*	1.1 × 10^14^ gc/kg	NCT03306277
	SMA	AAV9	*SMN1*	1.1 × 10^14^ gc/kg	NCT03837184
	SMA	AAV9	*SMN1*	N/A	NCT05614531
	SMA	AAV9	*SMN1*	N/A	NCT06191354
	SMA	AAV9	*SMN1*	N/A	NCT05747261
	SMA	N/A	*SMN1*	N/A	NCT06288230
	SMA	AAV9	*SMN1*	1.1 × 10^14^ gc/kg	NCT03955679
	SMA	AAV9	*SMN1*	6.7 × 10^13^–3.3 × 10^14^ gc/kg	NCT02122952
ICM/ICV	MPS II	AAV9	*IDS*	1.3 × 10^10^–2.9 × 10^11^ gc/g *	NCT03566043
	MPS II	AAV9	*IDS*	6.5 × 10^10^ gc/g *	NCT04571970
ICV/IVT	CLN5 BD	AAV9	*CLN5*	N/A	NCT05228145
IP/ICM/IT	GM2	AAVrh8	*HEXA/HEXB*	N/A	NCT04669535
IV/IT	AD	N/A	*TERT*	N/A	NCT04133454
N/A	MS	AAV9	*ATP7A*	1.0 × 10^12^–1.0 × 10^14^ gc/kg	NCT05507996

Abbreviation: *, gc/g of estimated brain mass; AADC, Aromatic L–amino Acid Decarboxylase Deficiency; AD, Alzheimer’s Disease; ALS, Amyotrophic Lateral Sclerosis; AMN, Adrenomyeloneuropathy; BD, Batten Disease; CD, Canavan Disease; CSF, Cerebrospinal Fluid; FTD, Frontotemporal Dementia; GAN, Giant Axonal Neuropathy; gc, Genome Copies; GD2, Gaucher disease Type 2; GM1, GM1 Gangliosidosis; GM2, GM2 Gangliosidosis; HD, Huntington disease; ICM, Intracisternal; ICV, Intracerebroventricular; IGHMBP2, IGHMBP2 related diseases; IP, Intraparenchymal; IT, Intrathecal; IV, Intravenous; IVT, Intravitreal; KD, Krabbe disease; LINCL, Late Infantile Neuronal Ceroid Lipofuscinosis; mi, miRNA targeting the gene; MLD, Metachromatic Leukodystrophy; MPS, Mucopolysaccharidosis; MS, Menkes Syndrome; MSA, Multiple System Atrophy; N/A, the data are not available; NGLY1, N–glycanase 1 deficiency; OTC, Ornithine Transcarbamylase Deficiency; PD, Parkinson’s disease; PKA, Phenylketonuria; RS, Rett syndrome; SMA, Spinal Muscular Atrophy; SP50, Spastic Paraplegia type 50; TLE, Temporal Lobe Epilepsy.

**Table 4:** Current testing and specifications for AAV-based products’ purity and safety depend on the type of study being conducted (clinical vs. preclinical).

Specifications Tests		Research Grade rAAV	Clinical Grade rAAV
Identity and integrity	Detection of therapeutic and regulator genes	YES	YES
	Analysis of VP proteins		YES
Content	Vector genome titer (VG)	YES	YES
	Infectious genome titer (IG)		YES
	Total vector particles	YES	YES
	Ratio full/empty particles		YES
Potency Assay	Activity (expression assay)	YES	YES
	Potency (functional activity)	YES	YES
Product-Related Impurities	Empty particles number		YES
	Replication competent AAV		YES
	Vector aggregates		YES
Process-Related Impurities	Host cell DNA		YES
	Helper plasmids or helper viruses DNA		YES
	Host cell protein		YES
	Residual production reagents and raw materials		YES
Extraneous agents	Sterility (Bacteria and fungi)		YES
	Bacterial endotoxins		YES
	Mycoplasma		YES
	Adventitious viruses		YES
	SV40 large T antigen		YES

## References

[1] Kimura S., Harashima H. (2020). Current Status and Challenges Associated with CNS-Targeted Gene Delivery across the BBB. Pharmaceutics.

[2] Udaka Y.T., Packer R.J. (2018). Pediatric Brain Tumors. Neurol. Clin..

[3] Ostrom Q.T., Patil N., Cioffi G., Waite K., Kruchko C., Barnholtz-Sloan J.S. (2020). CBTRUS Statistical Report: Primary Brain and Other Central Nervous System Tumors Diagnosed in the United States in 2013–2017. Neuro-oncology.

[4] Wimo A., Guerchet M., Ali G.C., Wu Y.T., Prina A.M., Winblad B., Jönsson L., Liu Z., Prince M. (2017). The worldwide costs of dementia 2015 and comparisons with 2010. Alzheimer’s Dement. J. Alzheimer’s Assoc..

[5] Alexander J.J. (2018). Blood-brain barrier (BBB) and the complement landscape. Mol Immunol..

[6] Piguet F., Alves S., Cartier N. (2017). Clinical Gene Therapy for Neurodegenerative Diseases: Past, Present, and Future. Hum. Gene Ther..

[7] Choudhury S.R., Hudry E., Maguire C.A., Sena-Esteves M., Breakefield X.O., Grandi P. (2017). Viral vectors for therapy of neurologic diseases. Neuropharmacology.

[8] Erel-Akbaba G., Carvalho L.A., Tian T., Zinter M., Akbaba H., Obeid P.J., Chiocca E.A., Weissleder R., Kantarci A.G., Tannous B.A. (2019). Radiation-Induced Targeted Nanoparticle-Based Gene Delivery for Brain Tumor Therapy. ACS Nano.

[9] Alnasser S.M. (2021). Review on mechanistic strategy of gene therapy in the treatment of disease. Gene.

[10] Liu D., Zhu M., Zhang Y., Diao Y. (2021). Crossing the blood-brain barrier with AAV vectors. Metab. Brain Dis..

[11] Foust K.D., Nurre E., Montgomery C.L., Hernandez A., Chan C.M., Kaspar B.K. (2009). Intravascular AAV9 preferentially targets neonatal neurons and adult astrocytes. Nat. Biotechnol..

[12] Stevens D., Claborn M.K., Gildon B.L., Kessler T.L., Walker C. (2020). Onasemnogene Abeparvovec-xioi: Gene Therapy for Spinal Muscular Atrophy. Ann. Pharmacother..

[13] Sun L., Li J., Xiao X. (2000). Overcoming adeno-associated virus vector size limitation through viral DNA heterodimerization. Nat. Med..

[14] Uren A.G., Kool J., Berns A., van Lohuizen M. (2005). Retroviral insertional mutagenesis: Past, present and future. Oncogene.

[15] Ellis B.L., Hirsch M.L., Barker J.C., Connelly J.P., Steininger R.J., Porteus M.H. (2013). A survey of ex vivo/in vitro transduction efficiency of mammalian primary cells and cell lines with Nine natural adeno-associated virus (AAV1-9) and one engineered adeno-associated virus serotype. Virol. J..

[16] Kotterman M.A., Schaffer D.V. (2014). Engineering adeno-associated viruses for clinical gene therapy. Nat. Rev. Genet..

[17] Mathiesen S.N., Lock J.L., Schoderboeck L., Abraham W.C., Hughes S.M. (2020). CNS Transduction Benefits of AAV-PHP.eB over AAV9 Are Dependent on Administration Route and Mouse Strain. Mol. Ther. Methods Clin. Dev..

[18] McCarty D.M. (2008). Self-complementary AAV Vectors; Advances and Applications. Mol. Ther..

[19] Calcedo R., Wilson J. (2013). Humoral Immune Response to AAV. Front. Immunol..

[20] Atchison R.W., Casto B.C., Hammon W.M. (1965). Adenovirus-Associated Defective Virus Particles. Science.

[21] Georg-Fries B., Biederlack S., Wolf J., zur Hausen H. (1984). Analysis of proteins, helper dependence, and seroepidemiology of a new human parvovirus. Virology.

[22] McPherson R.A., Rosenthal L.J., Rose J.A. (1985). Human cytomegalovirus completely helps adeno-associated virus replication. Virology.

[23] Bartlett J.S., Wilcher R., Samulski R.J. (2000). Infectious Entry Pathway of Adeno-Associated Virus and Adeno-Associated Virus Vectors. J. Virol..

[24] Boutin S., Monteilhet V., Veron P., Leborgne C., Benveniste O., Montus M.F., Masurier C. (2010). Prevalence of serum IgG and neutralizing factors against adeno-associated virus (AAV) types 1, 2, 5, 6, 8, and 9 in the healthy population: Implications for gene therapy using AAV vectors. Hum. Gene Ther..

[25] Podsakoff G., Wong K.K., Chatterjee S. (1994). Efficient gene transfer into nondividing cells by adeno-associated virus-based vectors. J. Virol..

[26] Srivastava A., Lusby E.W., Berns K.I. (1983). Nucleotide sequence and organization of the adeno-associated virus 2 genome. J. Virol..

[27] Rose J.A., Berns K.I., Hoggan M.D., Koczot F.J. (1969). Evidence for a single-stranded adenovirus-associated virus genome: Formation of a DNA density hybrid on release of viral DNA. Proc. Natl. Acad. Sci. USA.

[28] Kronenberg S., Kleinschmidt J.A., Bottcher B. (2001). Electron cryo-microscopy and image reconstruction of adeno-associated virus type 2 empty capsids. EMBO Rep..

[29] Sonntag F., Schmidt K., Kleinschmidt J.A. (2010). A viral assembly factor promotes AAV2 capsid formation in the nucleolus. Proc. Natl. Acad. Sci. USA.

[30] Pereira D.J., McCarty D.M., Muzyczka N. (1997). The adeno-associated virus (AAV) Rep protein acts as both a repressor and an activator to regulate AAV transcription during a productive infection. J. Virol..

[31] Mendelson E., Trempe J.P., Carter B.J. (1986). Identification of the trans-acting Rep proteins of adeno-associated virus by antibodies to a synthetic oligopeptide. J. Virol..

[32] Lusby E., Fife K.H., Berns K.I. (1980). Nucleotide sequence of the inverted terminal repetition in adeno-associated virus DNA. J. Virol..

[33] Ogden P.J., Kelsic E.D., Sinai S., Church G.M. (2019). Comprehensive AAV capsid fitness landscape reveals a viral gene and enables machine-guided design. Science.

[34] Elmore Z.C., Patrick Havlik L., Oh D.K., Anderson L., Daaboul G., Devlin G.W., Vincent H.A., Asokan A. (2021). The membrane associated accessory protein is an adeno-associated viral egress factor. Nat. Commun..

[35] Im D.S., Muzyczka N. (1990). The AAV origin binding protein Rep68 is an ATP-dependent site-specific endonuclease with DNA helicase activity. Cell.

[36] King J.A., Dubielzig R., Grimm D., Kleinschmidt J.A. (2001). DNA helicase-mediated packaging of adeno-associated virus type 2 genomes into preformed capsids. EMBO J..

[37] Snijder J., van de Waterbeemd M., Damoc E., Denisov E., Grinfeld D., Bennett A., Agbandje-McKenna M., Makarov A., Heck A.J. (2014). Defining the stoichiometry and cargo load of viral and bacterial nanoparticles by Orbitrap mass spectrometry. J. Am. Chem. Soc..

[38] Buller R.M., Rose J.A. (1978). Characterization of adenovirus-associated virus-induced polypeptides in KB cells. J. Virol..

[39] Johnson F.B., Ozer H.L., Hoggan M.D. (1971). Structural proteins of adenovirus-associated virus type 3. J. Virol..

[40] Wörner T.P., Bennett A., Habka S., Snijder J., Friese O., Powers T., Agbandje-McKenna M., Heck A.J.R. (2021). Adeno-associated virus capsid assembly is divergent and stochastic. Nat. Commun..

[41] Sonntag F., Bleker S., Leuchs B., Fischer R., Kleinschmidt J.A. (2006). Adeno-associated virus type 2 capsids with externalized VP1/VP2 trafficking domains are generated prior to passage through the cytoplasm and are maintained until uncoating occurs in the nucleus. J. Virol..

[42] Girod A., Wobus C.E., Zadori Z., Ried M., Leike K., Tijssen P., Kleinschmidt J.A., Hallek M. (2002). The VP1 capsid protein of adeno-associated virus type 2 is carrying a phospholipase A2 domain required for virus infectivity. J. Gen. Virol..

[43] Bosma B., du Plessis F., Ehlert E., Nijmeijer B., de Haan M., Petry H., Lubelski J. (2018). Optimization of viral protein ratios for production of rAAV serotype 5 in the baculovirus system. Gene Ther..

[44] Han J., Zhu L., Zhang J., Guo L., Sun X., Huang C., Xu K., Zhang Y., Li W., Zhou Q. (2022). Rational engineering of adeno-associated virus capsid enhances human hepatocyte tropism and reduces immunogenicity. Cell Prolif..

[45] Wu P., Xiao W., Conlon T., Hughes J., Agbandje-McKenna M., Ferkol T., Flotte T., Muzyczka N. (2000). Mutational analysis of the adeno-associated virus type 2 (AAV2) capsid gene and construction of AAV2 vectors with altered tropism. J. Virol..

[46] Münch R.C., Muth A., Muik A., Friedel T., Schmatz J., Dreier B., Trkola A., Plückthun A., Büning H., Buchholz C.J. (2015). Off-target-free gene delivery by affinity-purified receptor-targeted viral vectors. Nat. Commun..

[47] Zhong L., Li B., Jayandharan G., Mah C.S., Govindasamy L., Agbandje-McKenna M., Herzog R.W., Weigel-Van Aken K.A., Hobbs J.A., Zolotukhin S. (2008). Tyrosine-phosphorylation of AAV2 vectors and its consequences on viral intracellular trafficking and transgene expression. Virology.

[48] Aslanidi G.V., Rivers A.E., Ortiz L., Song L., Ling C., Govindasamy L., Van Vliet K., Tan M., Agbandje-McKenna M., Srivastava A. (2013). Optimization of the capsid of recombinant adeno-associated virus 2 (AAV2) vectors: The final threshold?. PLoS ONE.

[49] Kanaan N.M., Sellnow R.C., Boye S.L., Coberly B., Bennett A., Agbandje-McKenna M., Sortwell C.E., Hauswirth W.W., Boye S.E., Manfredsson F.P. (2017). Rationally Engineered AAV Capsids Improve Transduction and Volumetric Spread in the CNS. Molecular therapy. Nucleic Acids.

[50] Surdyka M.M., Figiel M. (2021). Retrograde capabilities of adeno-associated virus vectors in the central nervous system. Biotechnologia.

[51] Tervo D.G., Hwang B.Y., Viswanathan S., Gaj T., Lavzin M., Ritola K.D., Lindo S., Michael S., Kuleshova E., Ojala D. (2016). A Designer AAV Variant Permits Efficient Retrograde Access to Projection Neurons. Neuron.

[52] Weiss A.R., Liguore W.A., Domire J.S., Button D., McBride J.L. (2020). Intra-striatal AAV2.retro administration leads to extensive retrograde transport in the rhesus macaque brain: Implications for disease modeling and therapeutic development. Sci. Rep..

[53] Lin K., Zhong X., Li L., Ying M., Yang T., Zhang Z., He X., Xu F. (2020). AAV9-Retro mediates efficient transduction with axon terminal absorption and blood–brain barrier transportation. Mol. Brain.

[54] Padron E., Bowman V., Kaludov N., Govindasamy L., Levy H., Nick P., McKenna R., Muzyczka N., Chiorini J.A., Baker T.S. (2005). Structure of adeno-associated virus type 4. J. Virol..

[55] George L.A., Sullivan S.K., Giermasz A., Rasko J.E.J., Samelson-Jones B.J., Ducore J., Cuker A., Sullivan L.M., Majumdar S., Teitel J. (2017). Hemophilia B Gene Therapy with a High-Specific-Activity Factor IX Variant. N. Engl. J. Med..

[56] Schmidt M., Govindasamy L., Afione S., Kaludov N., Agbandje-McKenna M., Chiorini J.A. (2008). Molecular characterization of the heparin-dependent transduction domain on the capsid of a novel adeno-associated virus isolate, AAV(VR-942). J. Virol..

[57] Chen S., Kapturczak M., Loiler S.A., Zolotukhin S., Glushakova O.Y., Madsen K.M., Samulski R.J., Hauswirth W.W., Campbell-Thompson M., Berns K.I. (2005). Efficient transduction of vascular endothelial cells with recombinant adeno-associated virus serotype 1 and 5 vectors. Hum. Gene Ther..

[58] Kaludov N., Brown K.E., Walters R.W., Zabner J., Chiorini J.A. (2001). Adeno-associated virus serotype 4 (AAV4) and AAV5 both require sialic acid binding for hemagglutination and efficient transduction but differ in sialic acid linkage specificity. J. Virol..

[59] Seiler M.P., Miller A.D., Zabner J., Halbert C.L. (2006). Adeno-associated virus types 5 and 6 use distinct receptors for cell entry. Hum. Gene Ther..

[60] Halbert C.L., Allen J.M., Miller A.D. (2001). Adeno-associated virus type 6 (AAV6) vectors mediate efficient transduction of airway epithelial cells in mouse lungs compared to that of AAV2 vectors. J. Virol..

[61] Lerch T.F., Xie Q., Chapman M.S. (2010). The structure of adeno-associated virus serotype 3B (AAV-3B): Insights into receptor binding and immune evasion. Virology.

[62] Summerford C., Samulski R.J. (1998). Membrane-associated heparan sulfate proteoglycan is a receptor for adeno-associated virus type 2 virions. J. Virol..

[63] Bell C.L., Vandenberghe L.H., Bell P., Limberis M.P., Gao G.P., Van Vliet K., Agbandje-McKenna M., Wilson J.M. (2011). The AAV9 receptor and its modification to improve in vivo lung gene transfer in mice. J. Clin. Investig..

[64] Pillay S., Zou W., Cheng F., Puschnik A.S., Meyer N.L., Ganaie S.S., Deng X., Wosen J.E., Davulcu O., Yan Z. (2017). Adeno-associated Virus (AAV) Serotypes Have Distinctive Interactions with Domains of the Cellular AAV Receptor. J. Virol..

[65] Meisen W.H., Nejad Z.B., Hardy M., Zhao H., Oliverio O., Wang S., Hale C., Ollmann M.M., Collins P.J. (2020). Pooled Screens Identify GPR108 and TM9SF2 as Host Cell Factors Critical for AAV Transduction. Molecular therapy. Methods Clin. Dev..

[66] Akache B., Grimm D., Pandey K., Yant S.R., Xu H., Kay M.A. (2006). The 37/67-kilodalton laminin receptor is a receptor for adeno-associated virus serotypes 8, 2, 3, and 9. J. Virol..

[67] Summerford C., Bartlett J.S., Samulski R.J. (1999). AlphaVbeta5 integrin: A co-receptor for adeno-associated virus type 2 infection. Nat. Med..

[68] Asokan A., Hamra J.B., Govindasamy L., Agbandje-McKenna M., Samulski R.J. (2006). Adeno-associated virus type 2 contains an integrin alpha5beta1 binding domain essential for viral cell entry. J. Virol..

[69] Blackburn S.D., Steadman R.A., Johnson F.B. (2006). Attachment of adeno-associated virus type 3H to fibroblast growth factor receptor 1. Arch. Virol..

[70] Qing K., Mah C., Hansen J., Zhou S., Dwarki V., Srivastava A. (1999). Human fibroblast growth factor receptor 1 is a co-receptor for infection by adeno-associated virus 2. Nat. Med..

[71] Kurzeder C., Koppold B., Sauer G., Pabst S., Kreienberg R., Deissler H. (2007). CD9 promotes adeno-associated virus type 2 infection of mammary carcinoma cells with low cell surface expression of heparan sulphate proteoglycans. Int. J. Mol. Med..

[72] Ling C., Lu Y., Kalsi J.K., Jayandharan G.R., Li B., Ma W., Cheng B., Gee S.W., McGoogan K.E., Govindasamy L. (2010). Human hepatocyte growth factor receptor is a cellular coreceptor for adeno-associated virus serotype 3. Hum. Gene Ther..

[73] Di Pasquale G., Davidson B.L., Stein C.S., Martins I., Scudiero D., Monks A., Chiorini J.A. (2003). Identification of PDGFR as a receptor for AAV-5 transduction. Nat. Med..

[74] Weller M.L., Amornphimoltham P., Schmidt M., Wilson P.A., Gutkind J.S., Chiorini J.A. (2010). Epidermal growth factor receptor is a co-receptor for adeno-associated virus serotype 6. Nat. Med..

[75] Pillay S., Meyer N.L., Puschnik A.S., Davulcu O., Diep J., Ishikawa Y., Jae L.T., Wosen J.E., Nagamine C.M., Chapman M.S. (2016). An essential receptor for adeno-associated virus infection. Nature.

[76] Dudek A.M., Zabaleta N., Zinn E., Pillay S., Zengel J., Porter C., Franceschini J.S., Estelien R., Carette J.E., Zhou G.L. (2020). GPR108 Is a Highly Conserved AAV Entry Factor. Mol. Ther. J. Am. Soc. Gene Ther..

[77] Uhrig S., Coutelle O., Wiehe T., Perabo L., Hallek M., Buning H. (2012). Successful target cell transduction of capsid-engineered rAAV vectors requires clathrin-dependent endocytosis. Gene Ther..

[78] Nonnenmacher M., Weber T. (2011). Adeno-associated virus 2 infection requires endocytosis through the CLIC/GEEC pathway. Cell Host Microbe.

[79] Sanlioglu S., Benson P.K., Yang J., Atkinson E.M., Reynolds T., Engelhardt J.F. (2000). Endocytosis and nuclear trafficking of adeno-associated virus type 2 are controlled by rac1 and phosphatidylinositol-3 kinase activation. J. Virol..

[80] Liu Y., Joo K.I., Wang P. (2013). Endocytic processing of adeno-associated virus type 8 vectors for transduction of target cells. Gene Ther..

[81] Ding W., Zhang L.N., Yeaman C., Engelhardt J.F. (2006). rAAV2 traffics through both the late and the recycling endosomes in a dose-dependent fashion. Mol. Ther. J. Am. Soc. Gene Ther..

[82] Nonnenmacher M.E., Cintrat J.C., Gillet D., Weber T. (2015). Syntaxin 5-dependent retrograde transport to the trans-Golgi network is required for adeno-associated virus transduction. J. Virol..

[83] Madigan V.J., Berry G.E., Tyson T.O., Nardone-White D., Ark J., Elmore Z.C., Murlidharan G., Vincent H.A., Asokan A. (2020). The Golgi Calcium ATPase Pump Plays an Essential Role in Adeno-associated Virus Trafficking and Transduction. J. Virol..

[84] Stahnke S., Lux K., Uhrig S., Kreppel F., Hösel M., Coutelle O., Ogris M., Hallek M., Büning H. (2011). Intrinsic phospholipase A2 activity of adeno-associated virus is involved in endosomal escape of incoming particles. Virology.

[85] Nicolson S.C., Samulski R.J. (2014). Recombinant adeno-associated virus utilizes host cell nuclear import machinery to enter the nucleus. J. Virol..

[86] Johnson J.S., Samulski R.J. (2009). Enhancement of adeno-associated virus infection by mobilizing capsids into and out of the nucleolus. J. Virol..

[87] Hoad M., Roby J.A., Forwood J.K. (2021). Structural characterization of the porcine adeno-associated virus Po1 capsid protein binding to the nuclear trafficking protein importin alpha. FEBS Lett..

[88] Grieger J.C., Snowdy S., Samulski R.J. (2006). Separate basic region motifs within the adeno-associated virus capsid proteins are essential for infectivity and assembly. J. Virol..

[89] Wang Z., Ma H.I., Li J., Sun L., Zhang J., Xiao X. (2003). Rapid and highly efficient transduction by double-stranded adeno-associated virus vectors in vitro and in vivo. Gene Ther..

[90] Schreiber C.A., Sakuma T., Izumiya Y., Holditch S.J., Hickey R.D., Bressin R.K., Basu U., Koide K., Asokan A., Ikeda Y. (2015). An siRNA Screen Identifies the *U2 snRNP* Spliceosome as a Host Restriction Factor for Recombinant Adeno-associated Viruses. PLoS Pathog..

[91] Kotin R.M., Menninger J.C., Ward D.C., Berns K.I. (1991). Mapping and direct visualization of a region-specific viral DNA integration site on chromosome 19q13-qter. Genomics.

[92] Cataldi M.P., McCarty D.M. (2013). Hairpin-end conformation of adeno-associated virus genome determines interactions with DNA-repair pathways. Gene Ther..

[93] Wistuba A., Weger S., Kern A., Kleinschmidt J.A. (1995). Intermediates of adeno-associated virus type 2 assembly: Identification of soluble complexes containing Rep and Cap proteins. J. Virol..

[94] Gray S.J., Matagne V., Bachaboina L., Yadav S., Ojeda S.R., Samulski R.J. (2011). Preclinical Differences of Intravascular AAV9 Delivery to Neurons and Glia: A Comparative Study of Adult Mice and Nonhuman Primates. Mol. Ther..

[95] Manfredsson F.P., Rising A.C., Mandel R.J. (2009). AAV9: A potential blood-brain barrier buster. Mol. Ther..

[96] Nirwane A., Yao Y. (2019). Laminins and their receptors in the CNS. Biol. Rev. Camb. Philos. Soc..

[97] Bevan A.K., Duque S., Foust K.D., Morales P.R., Braun L., Schmelzer L., Chan C.M., McCrate M., Chicoine L.G., Coley B.D. (2011). Systemic Gene Delivery in Large Species for Targeting Spinal Cord, Brain, and Peripheral Tissues for Pediatric Disorders. Mol. Ther..

[98] FDA News Release—FDA Approves Innovative Gene Therapy to Treat Pediatric Patients with Spinal Muscular Atrophy, a Rare Disease and Leading Genetic Cause of Infant Mortality. https://www.fda.gov/news-events/press-announcements/fda-approves-innovative-gene-therapy-treat-pediatric-patients-spinal-muscular-atrophy-rare-disease.

[99] Kang L., Jin S., Wang J., Lv Z., Xin C., Tan C., Zhao M., Wang L., Liu J. (2023). AAV vectors applied to the treatment of CNS disorders: Clinical status and challenges. J. Control. Release.

[100] Zhang H., Yang B., Mu X., Ahmed S.S., Su Q., He R., Wang H., Mueller C., Sena-Esteves M., Brown R. (2011). Several rAAV vectors efficiently cross the blood-brain barrier and transduce neurons and astrocytes in the neonatal mouse central nervous system. Mol. Ther. J. Am. Soc. Gene Ther..

[101] Yang B., Li S., Wang H., Guo Y., Gessler D.J., Cao C., Su Q., Kramer J., Zhong L., Ahmed S.S. (2014). Global CNS Transduction of Adult Mice by Intravenously Delivered rAAVrh.8 and rAAVrh.10 and Nonhuman Primates by rAAVrh.10. Mol. Ther..

[102] Tanguy Y., Biferi M.G., Besse A., Astord S., Cohen-Tannoudji M., Marais T., Barkats M. (2015). Systemic AAVrh10 provides higher transgene expression than AAV9 in the brain and the spinal cord of neonatal mice. Front. Mol. Neurosci..

[103] Keeler A.M., Flotte T.R. (2019). Recombinant Adeno-Associated Virus Gene Therapy in Light of Luxturna (and Zolgensma and Glybera): Where Are We, and How Did We Get Here?. Annu. Rev. Virol..

[104] Blair H.A. (2022). Valoctocogene Roxaparvovec: First Approval. Drugs.

[105] Navale M.S., Bhosale M.K., Mohite M.M., Navale M.S. (2022). Hemgenix as First Gene Therapy for Treatment of Haemophilia B. Haemophilia.

[106] Upstaza Summary of Product Characteristics. https://www.ema.europa.eu/en/documents/product-information/upstaza-epar-product-information_en.pdf.

[107] Zolgensma Summary of Product Characteristics. https://www.ema.europa.eu/en/documents/product-information/zolgensma-epar-product-information_en.pdf.

[108] Lee E.J., Guenther C.M., Suh J. (2018). Adeno-associated virus (AAV) vectors: Rational design strategies for capsid engineering. Curr. Opin. Biomed. Eng..

[109] Weinmann J., Grimm D. (2017). Next-generation AAV vectors for clinical use: An ever-accelerating race. Virus Genes.

[110] Choudhury S.R., Harris A.F., Cabral D.J., Keeler A.M., Sapp E., Ferreira J.S., Gray-Edwards H.L., Johnson J.A., Johnson A.K., Su Q. (2016). Widespread Central Nervous System Gene Transfer and Silencing After Systemic Delivery of Novel AAV-AS Vector. Mol. Ther..

[111] Deverman B.E., Pravdo P.L., Simpson B.P., Kumar S.R., Chan K.Y., Banerjee A., Wu W.-L., Yang B., Huber N., Pasca S.P. (2016). Cre-dependent selection yields AAV variants for widespread gene transfer to the adult brain. Nat. Biotechnol..

[112] Matsuzaki Y., Konno A., Mochizuki R., Shinohara Y., Nitta K., Okada Y., Hirai H. (2018). Intravenous administration of the adeno-associated virus-PHP.B capsid fails to upregulate transduction efficiency in the marmoset brain. Neurosci. Lett..

[113] Hordeaux J., Wang Q., Katz N., Buza E.L., Bell P., Wilson J.M. (2018). The Neurotropic Properties of AAV-PHP.B Are Limited to C57BL/6J Mice. Mol. Ther..

[114] Huang Q., Chan K.Y., Tobey I.G., Chan Y.A., Poterba T., Boutros C.L., Balazs A.B., Daneman R., Bloom J.M., Seed C. (2019). Delivering genes across the blood-brain barrier: LY6A, a novel cellular receptor for AAV-PHP. B capsids. PLoS ONE.

[115] Chan K.Y., Jang M.J., Yoo B.B., Greenbaum A., Ravi N., Wu W.-L., Sánchez-Guardado L., Lois C., Mazmanian S.K., Deverman B.E. (2017). Engineered AAVs for efficient noninvasive gene delivery to the central and peripheral nervous systems. Nat. Neurosci..

[116] Wang D., Li S., Gessler D.J., Xie J., Zhong L., Li J., Tran K., Van Vliet K., Ren L., Su Q. (2018). A Rationally Engineered Capsid Variant of AAV9 for Systemic CNS-Directed and Peripheral Tissue-Detargeted Gene Delivery in Neonates. Mol. Ther. Methods Clin. Dev..

[117] Hanlon K.S., Meltzer J.C., Buzhdygan T., Cheng M.J., Sena-Esteves M., Bennett R.E., Sullivan T.P., Razmpour R., Gong Y., Ng C. (2019). Selection of an Efficient AAV Vector for Robust CNS Transgene Expression. Mol. Ther. Methods Clin. Dev..

[118] Yao Y., Wang J., Liu Y., Qu Y., Wang K., Zhang Y., Chang Y., Yang Z., Wan J., Liu J. (2022). Variants of the adeno-associated virus serotype 9 with enhanced penetration of the blood–brain barrier in rodents and primates. Nat. Biomed. Eng..

[119] Albright B.H., Storey C.M., Murlidharan G., Castellanos Rivera R.M., Berry G.E., Madigan V.J., Asokan A. (2018). Mapping the Structural Determinants Required for AAVrh.10 Transport across the Blood-Brain Barrier. Mol. Ther..

[120] Körbelin J., Dogbevia G., Michelfelder S., Ridder D.A., Hunger A., Wenzel J., Seismann H., Lampe M., Bannach J., Pasparakis M. (2016). A brain microvasculature endothelial cell-specific viral vector with the potential to treat neurovascular and neurological diseases. EMBO Mol. Med..

[121] Choudhury S.R., Fitzpatrick Z., Harris A.F., Maitland S.A., Ferreira J.S., Zhang Y., Ma S., Sharma R.B., Gray-Edwards H.L., Johnson J.A. (2016). In Vivo Selection Yields AAV-B1 Capsid for Central Nervous System and Muscle Gene Therapy. Mol. Ther..

[122] Lau C.-H., Ho J.W.-T., Lo P.K., Tin C. (2019). Targeted Transgene Activation in the Brain Tissue by Systemic Delivery of Engineered AAV1 Expressing CRISPRa. Mol. Ther. Nucleic Acids.

[123] Oller-Salvia B., Sánchez-Navarro M., Giralt E., Teixidó M. (2016). Blood–brain barrier shuttle peptides: An emerging paradigm for brain delivery. Chem. Soc. Rev..

[124] Zhang X., He T., Chai Z., Samulski R.J., Li C. (2018). Blood-brain barrier shuttle peptides enhance AAV transduction in the brain after systemic administration. Biomaterials.

[125] Kremer R., Williams A. (2024). AAV-BR1 does not target endothelial cells in Sprague Dawley rats unlike in mice. microPublication Biol..

[126] Flotte T.R., Zeitlin P.L., Reynolds T.C., Heald A.E., Pedersen P., Beck S., Conrad C.K., Brass-Ernst L., Humphries M., Sullivan K. (2003). Phase I Trial of Intranasal and Endobronchial Administration of a Recombinant Adeno-Associated Virus Serotype 2 (rAAV2)-CFTR Vector in Adult Cystic Fibrosis Patients: A Two-Part Clinical Study. Hum. Gene Ther..

[127] Zian W., Guojun X., Wai Chun T., Andreas G.S., Ijeoma F.U. (2019). Nose-to-Brain Delivery. J. Pharmacol. Exp. Ther..

[128] Liu F., Liu Y.-P., Lei G., Liu P., Chu Z., Gao C.-G., Dang Y.-H. (2017). Antidepressant effect of recombinant *NT4-NAP*/AAV on social isolated mice through intranasal route. Oncotarget.

[129] Ma X.-C., Liu P., Zhang X.-L., Jiang W.-H., Jia M., Wang C.-X., Dong Y.-Y., Dang Y.-H., Gao C.-G. (2016). Intranasal Delivery of Recombinant AAV Containing *BDNF* Fused with *HA2TAT*: A Potential Promising Therapy Strategy for Major Depressive Disorder. Sci. Rep..

[130] Chen C., Dong Y., Liu F., Gao C., Ji C., Dang Y., Ma X., Liu Y. (2020). A Study of Antidepressant Effect and Mechanism on Intranasal Delivery of *BDNF*-*HA2TAT*/AAV to Rats with Post-Stroke Depression. Neuropsychiatr. Dis. Treat..

[131] Belur L.R., Temme A., Podetz-Pedersen K.M., Riedl M., Vulchanova L., Robinson N., Hanson L.R., Kozarsky K.F., Orchard P.J., Frey W.H. (2017). Intranasal Adeno-Associated Virus Mediated Gene Delivery and Expression of Human Iduronidase in the Central Nervous System: A Noninvasive and Effective Approach for Prevention of Neurologic Disease in Mucopolysaccharidosis Type I. Hum. Gene Ther..

[132] Belur L.R., Romero M., Lee J., Podetz-Pedersen K.M., Nan Z., Riedl M.S., Vulchanova L., Kitto K.F., Fairbanks C.A., Kozarsky K.F. (2021). Comparative Effectiveness of Intracerebroventricular, Intrathecal, and Intranasal Routes of AAV9 Vector Administration for Genetic Therapy of Neurologic Disease in Murine Mucopolysaccharidosis Type I. Front. Mol. Neurosci..

[133] Qi B., Yang Y., Cheng Y., Sun D., Wang X., Khanna R., Ju W. (2020). Nasal delivery of a CRMP2-derived CBD3 adenovirus improves cognitive function and pathology in APP/PS1 transgenic mice. Mol. Brain.

[134] Ye D., Chen H. (2022). Focused Ultrasound-Mediated Intranasal Brain Drug Delivery Technique (FUSIN). Biomedical Engineering Technologies. Methods in Molecular Biology.

[135] Ye D., Yuan J., Yang Y., Yue Y., Hu Z., Fadera S., Chen H. (2022). Incisionless targeted adeno-associated viral vector delivery to the brain by focused ultrasound-mediated intranasal administration. eBioMedicine.

[136] Boulis N.M., Noordmans A.J., Song D.K., Imperiale M.J., Rubin A., Leone P., During M., Feldman E.L. (2003). Adeno-associated viral vector gene expression in the adult rat spinal cord following remote vector delivery. Neurobiol. Dis..

[137] Benkhelifa-Ziyyat S., Besse A., Roda M., Duque S., Astord S., Carcenac R., Marais T., Barkats M. (2013). Intramuscular scAAV9-*SMN* Injection Mediates Widespread Gene Delivery to the Spinal Cord and Decreases Disease Severity in SMA Mice. Mol. Ther..

[138] Towne C., Schneider B.L., Kieran D., Redmond D.E., Aebischer P. (2010). Efficient transduction of non-human primate motor neurons after intramuscular delivery of recombinant AAV serotype 6. Gene Ther..

[139] Hollis E.R., Kadoya K., Hirsch M., Samulski R.J., Tuszynski M.H. (2008). Efficient Retrograde Neuronal Transduction Utilizing Self-complementary AAV1. Mol. Ther..

[140] Lu Y.-Y., Wang L.-J., Muramatsu S.-i., Ikeguchi K., Fujimoto K.-i., Okada T., Mizukami H., Matsushita T., Hanazono Y., Kume A. (2003). Intramuscular injection of AAV-*GDNF* results in sustained expression of transgenic *GDNF*, and its delivery to spinal motoneurons by retrograde transport. Neurosci. Res..

[141] Wang L.-J., Lu Y.-Y., Muramatsu S.-i., Ikeguchi K., Fujimoto K.-i., Okada T., Mizukami H., Matsushita T., Hanazono Y., Kume A. (2002). Neuroprotective Effects of Glial Cell Line-Derived Neurotrophic Factor Mediated by an Adeno-Associated Virus Vector in a Transgenic Animal Model of Amyotrophic Lateral Sclerosis. J. Neurosci..

[142] Castle M.J., Gershenson Z.T., Giles A.R., Holzbaur E.L., Wolfe J.H. (2014). Adeno-associated virus serotypes 1, 8, and 9 share conserved mechanisms for anterograde and retrograde axonal transport. Hum. Gene Ther..

[143] Kelkar S., De B.P., Gao G., Wilson J.M., Crystal R.G., Leopold P.L. (2006). A Common Mechanism for Cytoplasmic Dynein-Dependent Microtubule Binding Shared among Adeno-Associated Virus and Adenovirus Serotypes. J. Virol..

[144] Boulis N.M., Willmarth N.E., Song D.K., Feldman E.L., Imperiale M.J. (2003). Intraneural colchicine inhibition of adenoviral and adeno-associated viral vector remote spinal cord gene delivery. Neurosurgery.

[145] Zheng H., Qiao C., Wang C.-H., Li J., Li J., Yuan Z., Zhang C., Xiao X. (2009). Efficient Retrograde Transport of Adeno-Associated Virus Type 8 to Spinal Cord and Dorsal Root Ganglion After Vector Delivery in Muscle. Hum. Gene Ther..

[146] Jan A., Richner M., Vægter C.B., Nyengaard J.R., Jensen P.H. (2019). Gene Transfer in Rodent Nervous Tissue Following Hindlimb Intramuscular Delivery of Recombinant Adeno-Associated Virus Serotypes AAV2/6, AAV2/8, and AAV2/9. Neurosci. Insights.

[147] Salegio E.A., Samaranch L., Kells A.P., Mittermeyer G., San Sebastian W., Zhou S., Beyer J., Forsayeth J., Bankiewicz K.S. (2013). Axonal transport of adeno-associated viral vectors is serotype-dependent. Gene Ther..

[148] Chen Z., Fan G., Li A., Yuan J., Xu T. (2020). rAAV2-Retro Enables Extensive and High-Efficient Transduction of Lower Motor Neurons following Intramuscular Injection. Mol. Ther. Methods Clin. Dev..

[149] Lin D., Fantz C.R., Levy B., Rafi M.A., Vogler C., Wenger D.A., Sands M.S. (2005). AAV2/5 vector expressing galactocerebrosidase ameliorates CNS disease in the murine model of globoid-cell leukodystrophy more efficiently than AAV2. Mol. Ther. J. Am. Soc. Gene Ther..

[150] Macauley S.L., Wong A.M., Shyng C., Augner D.P., Dearborn J.T., Pearse Y., Roberts M.S., Fowler S.C., Cooper J.D., Watterson D.M. (2014). An anti-neuroinflammatory that targets dysregulated glia enhances the efficacy of CNS-directed gene therapy in murine infantile neuronal ceroid lipofuscinosis. J. Neurosci. Off. J. Soc. Neurosci..

[151] Kells A.P., Forsayeth J., Bankiewicz K.S. (2012). Glial-derived neurotrophic factor gene transfer for Parkinson’s disease: Anterograde distribution of AAV2 vectors in the primate brain. Neurobiol. Dis..

[152] San Sebastian W., Samaranch L., Heller G., Kells A.P., Bringas J., Pivirotto P., Forsayeth J., Bankiewicz K.S. (2013). Adeno-associated virus type 6 is retrogradely transported in the non-human primate brain. Gene Ther..

[153] Zingg B., Chou X.-L., Zhang Z.-G., Mesik L., Liang F., Tao H.W., Zhang L.I. (2017). AAV-Mediated Anterograde Transsynaptic Tagging: Mapping Corticocollicular Input-Defined Neural Pathways for Defense Behaviors. Neuron.

[154] Castle M.J., Perlson E., Holzbaur E.L., Wolfe J.H. (2014). Long-distance axonal transport of AAV9 is driven by dynein and kinesin-2 and is trafficked in a highly motile Rab7-positive compartment. Mol. Ther. J. Am. Soc. Gene Ther..

[155] Cearley C.N., Wolfe J.H. (2007). A single injection of an adeno-associated virus vector into nuclei with divergent connections results in widespread vector distribution in the brain and global correction of a neurogenetic disease. J. Neurosci. Off. J. Soc. Neurosci..

[156] Broekman M.L.D., Tierney L.A., Benn C., Chawla P., Cha J.H., Sena-Esteves M. (2009). Mechanisms of distribution of mouse β-galactosidase in the adult GM1-gangliosidosis brain. Gene Ther..

[157] Rosenberg J.B., Sondhi D., Rubin D.G., Monette S., Chen A., Cram S., De B.P., Kaminsky S.M., Sevin C., Aubourg P. (2014). Comparative efficacy and safety of multiple routes of direct CNS administration of adeno-associated virus gene transfer vector serotype rh.10 expressing the human arylsulfatase A cDNA to nonhuman primates. Hum. Gene Ther. Clin. Dev..

[158] Treleaven C.M., Tamsett T.J., Bu J., Fidler J.A., Sardi S.P., Hurlbut G.D., Woodworth L.A., Cheng S.H., Passini M.A., Shihabuddin L.S. (2012). Gene transfer to the CNS is efficacious in immune-primed mice harboring physiologically relevant titers of anti-AAV antibodies. Mol. Ther..

[159] Gray S.J., Nagabhushan Kalburgi S., McCown T.J., Jude Samulski R. (2013). Global CNS gene delivery and evasion of anti-AAV-neutralizing antibodies by intrathecal AAV administration in non-human primates. Gene Ther.

[160] Recasens A., Ulusoy A., Kahle P.J., Di Monte D.A., Dehay B. (2018). In vivo models of alpha-synuclein transmission and propagation. Cell Tissue Res..

[161] Sehara Y., Inaba T., Urabe T., Kurosaki F., Urabe M., Kaneko N., Shimazaki K., Kawai K., Mizukami H. (2018). Survivin overexpression via adeno-associated virus vector Rh10 ameliorates ischemic damage after middle cerebral artery occlusion in rats. Eur. J. Neurosci..

[162] McCurdy V.J., Johnson A.K., Gray-Edwards H.L., Randle A.N., Brunson B.L., Morrison N.E., Salibi N., Johnson J.A., Hwang M., Beyers R.J. (2014). Sustained normalization of neurological disease after intracranial gene therapy in a feline model. Sci. Transl. Med..

[163] Ellinwood N.M., Ausseil J., Desmaris N., Bigou S., Liu S., Jens J.K., Snella E.M., Mohammed E.E., Thomson C.B., Raoul S. (2011). Safe, efficient, and reproducible gene therapy of the brain in the dog models of Sanfilippo and Hurler syndromes. Mol. Ther. J. Am. Soc. Gene Ther..

[164] Cressant A., Desmaris N., Verot L., Bréjot T., Froissart R., Vanier M.-T., Maire I., Heard J.M. (2004). Improved behavior and neuropathology in the mouse model of Sanfilippo type IIIB disease after adeno-associated virus-mediated gene transfer in the striatum. J. Neurosci. Off. J. Soc. Neurosci..

[165] Fu H., Samulski R.J., McCown T.J., Picornell Y.J., Fletcher D., Muenzer J. (2002). Neurological Correction of Lysosomal Storage in a Mucopolysaccharidosis IIIB Mouse Model by Adeno-associated Virus-Mediated Gene Delivery. Mol. Ther..

[166] Tardieu M., Zérah M., Gougeon M.-L., Ausseil J., de Bournonville S., Husson B., Zafeiriou D., Parenti G., Bourget P., Poirier B. (2017). Intracerebral gene therapy in children with mucopolysaccharidosis type IIIB syndrome: An uncontrolled phase 1/2 clinical trial. Lancet Neurol..

[167] Chen Z.-J., Broaddus W.C., Viswanathan R.R., Raghavan R., Gillies G.T. (2002). Intraparenchymal drug delivery via positive-pressure infusion: Experimental and modeling studies of poroelasticity in brain phantom gels. IEEE Trans. Biomed. Eng..

[168] Linninger A.A., Somayaji M.R., Mekarski M., Zhang L. (2008). Prediction of convection-enhanced drug delivery to the human brain. J. Theor. Biol..

[169] Kim I., Paek S., Nelson B.D., Knight E.J., Marsh M.P., Bieber A.J., Bennet K.E., Lee K.H. (2014). Implementation of a chronic unilateral intraparenchymal drug delivery system in a swine model. J. Neurosci. Methods.

[170] Barua N.U., Gill S.S., Love S. (2014). Convection-enhanced drug delivery to the brain: Therapeutic potential and neuropathological considerations. Brain Pathol..

[171] Zhang L., Yang M., Jiang M. (2012). Mathematical Modeling for Convection-Enhanced Drug Delivery. Procedia Eng..

[172] Bankiewicz K.S., Sudhakar V., Samaranch L., San Sebastian W., Bringas J., Forsayeth J. (2016). AAV viral vector delivery to the brain by shape-conforming MR-guided infusions. J. Control. Release.

[173] Hocquemiller M., Giersch L., Audrain M., Parker S., Cartier N. (2016). Adeno-Associated Virus-Based Gene Therapy for CNS Diseases. Hum. Gene Ther..

[174] Liguore W.A., Domire J.S., Button D., Wang Y., Dufour B.D., Srinivasan S., McBride J.L. (2019). AAV-PHP. B administration results in a differential pattern of CNS biodistribution in non-human primates compared with mice. Mol. Ther..

[175] Samaranch L., Salegio E.A., San Sebastian W., Kells A.P., Bringas J.R., Forsayeth J., Bankiewicz K.S. (2013). Strong cortical and spinal cord transduction after AAV7 and AAV9 delivery into the cerebrospinal fluid of nonhuman primates. Hum. Gene Ther..

[176] Bey K., Ciron C., Dubreil L., Deniaud J., Ledevin M., Cristini J., Blouin V., Aubourg P., Colle M.A. (2017). Efficient CNS targeting in adult mice by intrathecal infusion of single-stranded AAV9-*GFP* for gene therapy of neurological disorders. Gene Ther..

[177] Hinderer C., Bell P., Katz N., Vite C.H., Louboutin J.-P., Bote E., Yu H., Zhu Y., Casal M.L., Bagel J. (2017). Evaluation of Intrathecal Routes of Administration for Adeno-Associated Viral Vectors in Large Animals. Hum. Gene Ther..

[178] Federici T., Taub J.S., Baum G.R., Gray S.J., Grieger J.C., Matthews K.A., Handy C.R., Passini M.A., Samulski R.J., Boulis N.M. (2012). Robust spinal motor neuron transduction following intrathecal delivery of AAV9 in pigs. Gene Ther..

[179] Maguire C.A., Meijer D.H., LeRoy S.G., Tierney L.A., Broekman M.L., Costa F.F., Breakefield X.O., Stemmer-Rachamimov A., Sena-Esteves M. (2008). Preventing growth of brain tumors by creating a zone of resistance. Mol. Ther..

[180] Hinderer C., Katz N., Dyer C., Goode T., Johansson J., Bell P., Richman L., Buza E., Wilson J.M. (2020). Translational Feasibility of Lumbar Puncture for Intrathecal AAV Administration. Mol. Ther. Methods Clin. Dev..

[181] Meijer D.H., Maguire C.A., LeRoy S.G., Sena-Esteves M. (2009). Controlling brain tumor growth by intraventricular administration of an AAV vector encoding *IFN*-*β*. Cancer Gene Ther..

[182] Hinderer C., Bell P., Vite C.H., Louboutin J.P., Grant R., Bote E., Yu H., Pukenas B., Hurst R., Wilson J.M. (2014). Widespread gene transfer in the central nervous system of cynomolgus macaques following delivery of AAV9 into the cisterna magna. Molecular therapy. Methods Clin. Dev..

[183] Hordeaux J., Hinderer C., Buza E.L., Louboutin J.P., Jahan T., Bell P., Chichester J.A., Tarantal A.F., Wilson J.M. (2019). Safe and Sustained Expression of Human Iduronidase After Intrathecal Administration of Adeno-Associated Virus Serotype 9 in Infant Rhesus Monkeys. Hum. Gene Ther..

[184] Samaranch L., Bringas J., Pivirotto P., Sebastian W.S., Forsayeth J., Bankiewicz K. (2016). Cerebellomedullary Cistern Delivery for AAV-Based Gene Therapy: A Technical Note for Nonhuman Primates. Hum. Gene Ther. Methods.

[185] Taghian T., Marosfoi M.G., Puri A.S., Cataltepe O.I., King R.M., Diffie E.B., Maguire A.S., Martin D.R., Fernau D., Batista A.R. (2020). A Safe and Reliable Technique for CNS Delivery of AAV Vectors in the Cisterna Magna. Mol. Ther..

[186] Sorrentino N.C., Maffia V., Strollo S., Cacace V., Romagnoli N., Manfredi A., Ventrella D., Dondi F., Barone F., Giunti M. (2016). A Comprehensive Map of CNS Transduction by Eight Recombinant Adeno-associated Virus Serotypes Upon Cerebrospinal Fluid Administration in Pigs. Mol. Ther..

[187] Bey K., Deniaud J., Dubreil L., Joussemet B., Cristini J., Ciron C., Hordeaux J., Le Boulc’h M., Marche K., Maquigneau M. (2020). Intra-CSF AAV9 and AAVrh10 Administration in Nonhuman Primates: Promising Routes and Vectors for Which Neurological Diseases?. Mol. Ther. Methods Clin. Dev..

[188] Rosenberg J.B., Kaplitt M.G., De B.P., Chen A., Flagiello T., Salami C., Pey E., Zhao L., Ricart Arbona R.J., Monette S. (2018). AAVrh. 10-mediated *APOE2* central nervous system gene therapy for APOE4-associated Alzheimer’s disease. Hum. Gene Ther. Clin. Dev..

[189] Barker R.A., Bjorklund A., Gash D.M., Whone A., Van Laar A., Kordower J.H., Bankiewicz K., Kieburtz K., Saarma M., Booms S. (2020). GDNF and Parkinson’s Disease: Where Next? A Summary from a Recent Workshop. J Park. Dis..

[190] Menozzi E., Toffoli M., Schapira A.H.V. (2023). Targeting the *GBA1* pathway to slow Parkinson disease: Insights into clinical aspects, pathogenic mechanisms and new therapeutic avenues. Pharmacol Ther.

[191] Sehara Y., Fujimoto K.I., Ikeguchi K., Katakai Y., Ono F., Takino N., Ito M., Ozawa K., Muramatsu S.I. (2017). Persistent expression of dopamine-synthesizing enzymes 15 years after gene transfer in a primate model of Parkinson’s disease. Hum. Gene Ther. Clin. Dev..

[192] Naidoo J., Stanek L.M., Ohno K., Trewman S., Samaranch L., Hadaczek P., O’Riordan C., Sullivan J., San Sebastian W., Bringas J.R. (2018). Extensive Transduction and Enhanced Spread of a Modified AAV2 Capsid in the Non-human Primate CNS. Mol. Ther..

[193] Mendell J.R., Al-Zaidy S., Shell R., Arnold W.D., Rodino-Klapac L.R., Prior T.W., Lowes L., Alfano L., Berry K., Church K. (2017). Single-Dose Gene-Replacement Therapy for Spinal Muscular Atrophy. N. Engl. J. Med..

[194] Gonzalez-Latapi P., Bayram E., Litvan I., Marras C. (2021). Cognitive Impairment in Parkinson’s Disease: Epidemiology, Clinical Profile, Protective and Risk Factors. Behav. Sci..

[195] Grochans S., Cybulska A.M., Simińska D., Korbecki J., Kojder K., Chlubek D., Baranowska-Bosiacka I. (2022). Epidemiology of Glioblastoma Multiforme–Literature Review. Cancers.

[196] Dalaker T.O., Zivadinov R., Ramasamy D.P., Beyer M.K., Alves G., Bronnick K.S., Tysnes O.-B., Aarsland D., Larsen J.P. (2011). Ventricular enlargement and mild cognitive impairment in early Parkinson’s disease. Mov. Disord..

[197] Hadaczek P., Mirek H., Bringas J., Cunningham J., Bankiewicz K. (2004). Basic fibroblast growth factor enhances transduction, distribution, and axonal transport of adeno-associated virus type 2 vector in rat brain. Hum. Gene Ther..

[198] Aschauer D.F., Kreuz S., Rumpel S. (2013). Analysis of Transduction Efficiency, Tropism and Axonal Transport of AAV Serotypes 1, 2, 5, 6, 8 and 9 in the Mouse Brain. PLoS ONE.

[199] Hinderer C., Katz N., Buza E.L., Dyer C., Goode T., Bell P., Richman L.K., Wilson J.M. (2018). Severe Toxicity in Nonhuman Primates and Piglets Following High-Dose Intravenous Administration of an Adeno-Associated Virus Vector Expressing Human *SMN*. Hum. Gene Ther..

[200] Hudry E., Aihara F., Meseck E., Mansfield K., McElroy C., Chand D., Tukov F.F., Penraat K. (2023). Liver injury in cynomolgus monkeys following intravenous and intrathecal scAAV9 gene therapy delivery. Mol. Ther..

[201] Duan D. (2023). Lethal immunotoxicity in high-dose systemic AAV therapy. Mol. Ther..

[202] Shen W., Liu S., Ou L. (2022). rAAV immunogenicity, toxicity, and durability in 255 clinical trials: A meta-analysis. Front. Immunol..

[203] Hinderer C., Bell P., Louboutin J.P., Zhu Y., Yu H., Lin G., Choa R., Gurda B.L., Bagel J., O’Donnell P. (2015). Neonatal Systemic AAV Induces Tolerance to CNS Gene Therapy in MPS I Dogs and Nonhuman Primates. Mol. Ther. J. Am. Soc. Gene Ther..

[204] Mueller C., Berry J.D., McKenna-Yasek D.M., Gernoux G., Owegi M.A., Pothier L.M., Douthwright C.L., Gelevski D., Luppino S.D., Blackwood M. (2020). *SOD1* suppression with adeno-associated virus and microRNA in familial ALS. N. Engl. J. Med..

[205] Fader K.A., Pardo I.D., Kovi R.C., Somps C.J., Wang H.H., Vaidya V.S., Ramaiah S.K., Sirivelu M.P. (2022). Circulating neurofilament light chain as a promising biomarker of AAV-induced dorsal root ganglia toxicity in nonclinical toxicology species. Molecular therapy. Methods Clin. Dev..

[206] De Andres J., Hayek S., Perruchoud C., Lawrence M.M., Reina M.A., De Andres-Serrano C., Rubio-Haro R., Hunt M., Yaksh T.L. (2022). Intrathecal Drug Delivery: Advances and Applications in the Management of Chronic Pain Patient. Front. Pain Res..

[207] Shen J., Swift B., Mamelok R., Pine S., Sinclair J., Attar M. (2019). Design and Conduct Considerations for First-in-Human Trials. Clin. Transl. Sci..

[208] Burr A., Erickson P., Bento R., Shama K., Roth C., Parekkadan B. (2022). Allometric-like scaling of AAV gene therapy for systemic protein delivery. Mol. Ther. Methods Clin. Dev..

[209] Thwaite R., Pagès G., Chillón M., Bosch A. (2015). AAVrh.10 immunogenicity in mice and humans. Relevance of antibody cross-reactivity in human gene therapy. Gene Ther..

[210] Battulin N., Korablev A., Ryzhkova A., Smirnov A., Kabirova E., Khabarova A., Lagunov T., Serova I., Serov O. (2022). The human EF1a promoter does not provide expression of the transgene in mice. Transgenic Res..

[211] Williams D.A. (2005). FDA Guidance Document on Monitoring Delayed Adverse Events a Good First Start. Mol. Ther..

[212] Chand D.H., Zaidman C., Arya K., Millner R., Farrar M.A., Mackie F.E., Goedeker N.L., Dharnidharka V.R., Dandamudi R., Reyna S.P. (2021). Thrombotic Microangiopathy Following Onasemnogene Abeparvovec for Spinal Muscular Atrophy: A Case Series. J. Pediatr..

[213] Wijngaarde C.A., Huisman A., Wadman R.I., Cuppen I., Stam M., Heitink-Pollé K.M., Groen E.J., Schutgens R.E., van der Pol W.-L. (2020). Abnormal coagulation parameters are a common non-neuromuscular feature in patients with spinal muscular atrophy. J. Neurol. Neurosurg..

[214] Brocklebank V., Wood K.M., Kavanagh D. (2018). Thrombotic microangiopathy and the kidney. Clin. J. Am. Soc. Nephrol..

[215] Waldrop M.A., Karingada C., Storey M.A., Powers B., Iammarino M.A., Miller N.F., Alfano L.N., Noritz G., Rossman I., Ginsberg M. (2020). Gene therapy for spinal muscular atrophy: Safety and early outcomes. Pediatrics.

[216] Gaudet D., Stroes E.S., Méthot J., Brisson D., Tremblay K., Bernelot Moens S.J., Iotti G., Rastelletti I., Ardigo D., Corzo D. (2016). Long-Term Retrospective Analysis of Gene Therapy with Alipogene Tiparvovec and Its Effect on Lipoprotein Lipase Deficiency-Induced Pancreatitis. Hum. Gene Ther..

[217] Leroy B.P., Fischer M.D., Flannery J.G., MacLaren R.E., Dalkara D., Scholl H.P.N., Chung D.C., Spera C., Viriato D., Banhazi J. (2023). Gene Therapy for Inherited Retinal Disease: Long-Term Durability of Effect. Ophthalmic Res..

[218] Nathwani A.C., Reiss U.M., Tuddenham E.G., Rosales C., Chowdary P., McIntosh J., Della Peruta M., Lheriteau E., Patel N., Raj D. (2014). Long-term safety and efficacy of factor IX gene therapy in hemophilia B. N. Engl. J. Med..

[219] Nathwani A.C., Reiss U., Tuddenham E., Chowdary P., McIntosh J., Riddell A., Pie J., Mahlangu J.N., Recht M., Shen Y.-M. (2018). Adeno-associated mediated gene transfer for hemophilia B: 8 year follow up and impact of removing “empty viral particles” on safety and efficacy of gene transfer. J. Blood.

[220] Hösel M., Broxtermann M., Janicki H., Esser K., Arzberger S., Hartmann P., Gillen S., Kleeff J., Stabenow D., Odenthal M. (2012). Toll-like receptor 2-mediated innate immune response in human nonparenchymal liver cells toward adeno-associated viral vectors. Hepatology.

[221] Mueller C., Gernoux G., Gruntman A.M., Borel F., Reeves E.P., Calcedo R., Rouhani F.N., Yachnis A., Humphries M., Campbell-Thompson M. (2017). 5 Year expression and neutrophil defect repair after gene therapy in alpha-1 antitrypsin deficiency. Mol. Ther..

[222] Novartis Releases Long-Term Zolgensma Data. https://www.biopharma-reporter.com/Article/2023/03/20/Novartis-releases-long-term-Zolgensma-data?utm_source=copyright&utm_medium=OnSite&utm_campaign=copyright.

[223] Greig J.A., Martins K.M., Breton C., Lamontagne R.J., Zhu Y., He Z., White J., Zhu J.-X., Chichester J.A., Zheng Q. (2023). Integrated vector genomes may contribute to long-term expression in primate liver after AAV administration. Nat. Biotechnol..

[224] Kishimoto T.K., Samulski R.J. (2022). Addressing high dose AAV toxicity—‘one and done’ or ‘slower and lower’?. Expert Opin. Biol. Ther..

[225] Russell D.W. (2007). AAV Vectors, Insertional Mutagenesis, and Cancer. Mol. Ther..

[226] Donsante A., Vogler C., Muzyczka N., Crawford J., Barker J., Flotte T., Campbell-Thompson M., Daly T., Sands M. (2001). Observed incidence of tumorigenesis in long-term rodent studies of rAAV vectors. Gene Ther..

[227] Donsante A., Miller D.G., Li Y., Vogler C., Brunt E.M., Russell D.W., Sands M.S. (2007). AAV vector integration sites in mouse hepatocellular carcinoma. Science.

[228] Assaf B.T., Whiteley L.O. (2018). Considerations for preclinical safety assessment of adeno-associated virus gene therapy products. Toxicol. Pathol..

[229] Long Term Follow-up after Administration of Human Gene Therapy Products. https://www.fda.gov/regulatory-information/search-fda-guidance-documents/long-term-follow-after-administration-human-gene-therapy-products.

[230] ICH S12 Guideline on Nonclinical Biodistribution Considerations for Gene Therapy Products. https://www.ema.europa.eu/en/documents/regulatory-procedural-guideline/ich-guideline-s12-nonclinical-biodistribution-considerations-gene-therapy-products-step-5_en.pdf.

[231] Arruda V.R., Fields P.A., Milner R., Wainwright L., De Miguel M.P., Donovan P.J., Herzog R.W., Nichols T.C., Biegel J.A., Razavi M. (2001). Lack of Germline Transmission of Vector Sequences Following Systemic Administration of Recombinant AAV-2 Vector in Males. Mol. Ther..

[232] Pachori A.S., Melo L.G., Zhang L., Loda M., Pratt R.E., Dzau V.J. (2004). Potential for germ line transmission after intramyocardial gene delivery by adeno-associated virus. Biochem. Biophys. Res. Commun..

[233] Kay M.A., Manno C.S., Ragni M.V., Larson P.J., Couto L.B., McClelland A., Glader B., Chew A.J., Tai S.J., Herzog R.W. (2000). Evidence for gene transfer and expression of factor IX in haemophilia B patients treated with an AAV vector. Nat. Genet..

[234] Favaro P., Downey H.D., Zhou J.S., Wright J.F., Hauck B., Mingozzi F., High K.A., Arruda V.R. (2009). Host and Vector-dependent Effects on the Risk of Germline Transmission of AAV Vectors. Mol. Ther..

[235] Brady M.L., Raghavan R., Alexander A., Kubota K., Sillay K., Emborg M.E. (2013). Pathways of infusate loss during convection-enhanced delivery into the putamen nucleus. Stereotact. Funct. Neurosurg..

[236] Vazquez L.C., Hagel E., Willenberg B.J., Dai W., Casanova F., Batich C.D., Sarntinoranont M. (2012). Polymer-coated cannulas for the reduction of backflow during intraparenchymal infusions. J. Mater. Sci. Mater. Med..

[237] Cook A.M., Mieure K.D., Owen R.D., Pesaturo A.B., Hatton J. (2009). Intracerebroventricular Administration of Drugs. Pharmacother. J. Hum. Pharmacol. Drug Ther..

[238] Luxturna Summary of Product Characteristics. https://www.ema.europa.eu/en/documents/product-information/luxturna-epar-product-information_en.pdf.

[239] Morrison C. (2015). $1-million price tag set for Glybera gene therapy. Nat. Biotechnol..

[240] Keam S.J. (2022). Eladocagene Exuparvovec: First Approval. Drugs.

[241] Ruiz-Garcia A., Bermejo M., Moss A., Casabo V.G. (2008). Pharmacokinetics in Drug Discovery. J. Pharm. Sci..

[242] Guideline on the Quality, Non-Clinical and Clinical Aspects of Gene Therapy Medicinal Products. https://www.ema.europa.eu/en/documents/scientific-guideline/guideline-quality-non-clinical-and-clinical-aspects-gene-therapy-medicinal-products_en.pdf.

[243] Issa S.S., Shaimardanova A.A., Solovyeva V.V., Rizvanov A.A.J.C. (2023). Various AAV Serotypes and Their Applications in Gene Therapy: An Overview. Cells.

[244] Levy G. (1994). Pharmacologic target-mediated drug disposition. Clin. Pharmacol. Ther..

[245] Rose R.H., Sepp A., Stader F., Gill K.L., Liu C., Gardner I. (2022). Application of physiologically based pharmacokinetic models for therapeutic proteins and other novel modalities. Xenobiotica.

[246] Słyk Ż., Wrzesień R., Barszcz S., Gawrychowski K., Małecki M. (2024). Adeno-associated virus vector hydrogel formulations for brain cancer gene therapy applications. Biomed. Pharmacother..

[247] Dheekollu J., Wiedmer A., Sentana-Lledo D., Cassel J., Messick T., Lieberman P.M. (2016). *HCF1* and *OCT2* Cooperate with *EBNA1* To Enhance OriP-Dependent Transcription and Episome Maintenance of Latent Epstein-Barr Virus. J. Virol..

[248] Akache B., Grimm D., Shen X., Fuess S., Yant S.R., Glazer D.S., Park J., Kay M.A. (2007). A Two-hybrid Screen Identifies Cathepsins B and L as Uncoating Factors for Adeno-associated Virus 2 and 8. Mol. Ther..

[249] Bentley P., Tan Min Jie A., McBride Alison A., White Elizabeth A., Howley Peter M., Banks L. (2019). The SMC5/6 Complex Interacts with the Papillomavirus E2 Protein and Influences Maintenance of Viral Episomal DNA. J. Virol..

[250] Chen Y.H., Keiser M.S., Davidson B.L. (2018). Adeno-Associated Virus Production, Purification, and Titering. Curr. Protoc. Mouse Biol..

[251] Tang Q., Keeler A.M., Zhang S., Su Q., Lyu Z., Cheng Y., Gao G., Flotte T.R. (2020). Two-Plasmid Packaging System for Recombinant Adeno-Associated Virus. BioResearch Open Access.

[252] Sandro Q., Relizani K., Benchaouir R., Manfredsson F.P., Benskey M.J. (2019). AAV Production Using Baculovirus Expression Vector System. Viral Vectors for Gene Therapy: Methods and Protocols.

[253] Merten O.-W. (2024). Development of Stable Packaging and Producer Cell Lines for the Production of AAV Vectors. Microorganisms.

[254] Chen X., Lim D.A., Lawlor M.W., Dimmock D., Vite C.H., Lester T., Tavakkoli F., Sadhu C., Prasad S., Gray S.J. (2023). Biodistribution of Adeno-Associated Virus Gene Therapy Following Cerebrospinal Fluid-Directed Administration. Hum. Gene Ther..

[255] Tran N.T., Lecomte E., Saleun S., Namkung S., Robin C., Weber K., Devine E., Blouin V., Adjali O., Ayuso E. (2022). Human and Insect Cell-Produced Recombinant Adeno-Associated Viruses Show Differences in Genome Heterogeneity. Hum. Gene Ther..

[256] O’Connor D.M., Lutomski C., Jarrold M.F., Boulis N.M., Donsante A. (2019). Lot-to-Lot Variation in Adeno-Associated Virus Serotype 9 (AAV9) Preparations. Hum. Gene Ther. Methods.

[257] Wright J.F. (2014). Product-Related Impurities in Clinical-Grade Recombinant AAV Vectors: Characterization and Risk Assessment. Biomedicines.

[258] Gao K., Li M., Zhong L., Su Q., Li J., Li S., He R., Zhang Y., Hendricks G., Wang J. (2014). Empty Virions In AAV8 Vector Preparations Reduce Transduction Efficiency And May Cause Total Viral Particle Dose-Limiting Side-Effects. Mol. Ther. Methods Clin. Dev..

[259] Allen J.M., Debelak D.J., Reynolds T.C., Miller A.D. (1997). Identification and elimination of replication-competent adeno-associated virus (AAV) that can arise by nonhomologous recombination during AAV vector production. J. Virol..

[260] Rosenberg A.S. (2006). Effects of protein aggregates: An immunologic perspective. AAPS J..

[261] Hauck B., Murphy S.L., Smith P.H., Qu G., Liu X., Zelenaia O., Mingozzi F., Sommer J.M., High K.A., Wright J.F. (2009). Undetectable transcription of cap in a clinical AAV vector: Implications for preformed capsid in immune responses. Mol. Ther..

[262] Chadeuf G., Ciron C., Moullier P., Salvetti A. (2005). Evidence for encapsidation of prokaryotic sequences during recombinant adeno-associated virus production and their in vivo persistence after vector delivery. Mol. Ther..

[263] Grachev V., Magrath D., Griffiths E. (1998). WHO requirements for the use of animal cells as in vitro substrates for the production of biologicals (Requirements for biological susbstances no. 50). Biol. J. Int. Assoc. Biol. Stand..

[264] Sheng L., Cai F., Zhu Y., Pal A., Athanasiou M., Orrison B., Blair D.G., Hughes S.H., Coffin J.M., Lewis A.M. (2008). Oncogenicity of DNA in vivo: Tumor induction with expression plasmids for activated H-ras and c-myc. Biol. J. Int. Assoc. Biol. Stand..

[265] Bucher K., Rodríguez-Bocanegra E., Wissinger B., Strasser T., Clark S.J., Birkenfeld A.L., Siegel-Axel D., Fischer M.D. (2023). Extra-viral DNA in adeno-associated viral vector preparations induces TLR9-dependent innate immune responses in human plasmacytoid dendritic cells. Sci. Rep..

[266] Jawa V., Joubert M.K., Zhang Q., Deshpande M., Hapuarachchi S., Hall M.P., Flynn G.C. (2016). Evaluating Immunogenicity Risk Due to Host Cell Protein Impurities in Antibody-Based Biotherapeutics. AAPS J..

[267] Greig J.A., Nordin J.M.L., Bote E., Makaron L., Garnett M.E., Kattenhorn L.M., Bell P., Goode T., Wilson J.M. (2016). Impact of intravenous infusion time on AAV8 vector pharmacokinetics, safety, and liver transduction in cynomolgus macaques. Mol. Ther. Methods Clin. Dev..

[268] Compton D.R., DeMarco S.J., Yalamanchili P. (2022). AAV2-hAADC (Eladocagene Exuparvovec) Biodistribution and Expression: Superiority of Intraputaminal versus Intracerebroventricular and Intrathecal (Lumbar) Routes of Administration. Int. J. Toxicol..

[269] Zincarelli C., Soltys S., Rengo G., Rabinowitz J.E. (2008). Analysis of AAV serotypes 1–9 mediated gene expression and tropism in mice after systemic injection. Mol. Ther..

[270] Kotchey N.M., Adachi K., Zahid M., Inagaki K., Charan R., Parker R.S., Nakai H. (2011). A Potential Role of Distinctively Delayed Blood Clearance of Recombinant Adeno-associated Virus Serotype 9 in Robust Cardiac Transduction. Mol. Ther..

[271] van Gestel M.A., Boender A.J., de Vrind V.A.J., Garner K.M., Luijendijk M.C.M., Adan R.A.H. (2014). Recombinant Adeno-Associated Virus: Efficient Transduction of the Rat VMH and Clearance from Blood. PLoS ONE.

[272] García-Olloqui P., Rodriguez-Madoz J.R., Di Scala M., Abizanda G., Vales Á., Olagüe C., Iglesias-García O., Larequi E., Aguado-Alvaro L.P., Ruiz-Villalba A. (2020). Effect of heart ischemia and administration route on biodistribution and transduction efficiency of AAV9 vectors. J. Tissue Eng. Regen. Med..

[273] Chen Y.H., Claflin K., Geoghegan J.C., Davidson B.L. (2012). Sialic Acid Deposition Impairs the Utility of AAV9, but Not Peptide-modified AAVs for Brain Gene Therapy in a Mouse Model of Lysosomal Storage Disease. Mol. Ther..

[274] Piechnik M., Amendum P.C., Sawamoto K., Stapleton M., Khan S., Fnu N., Álvarez V., Pachon A.M., Danos O., Bruder J.T. (2022). Sex Difference Leads to Differential Gene Expression Patterns and Therapeutic Efficacy in Mucopolysaccharidosis IVA Murine Model Receiving AAV8 Gene Therapy. Int. J. Mol. Sci..

[275] Maguire C.A., Crommentuijn M.H., Mu D., Hudry E., Serrano-Pozo A., Hyman B.T., Tannous B.A. (2013). Mouse gender influences brain transduction by intravascularly administered AAV9. Mol. Ther. J. Am. Soc. Gene Ther..

[276] Maguire A.M., High K.A., Auricchio A., Wright J.F., Pierce E.A., Testa F., Mingozzi F., Bennicelli J.L., Ying G.S., Rossi S. (2009). Age-dependent effects of RPE65 gene therapy for Leber’s congenital amaurosis: A phase 1 dose-escalation trial. Lancet.

[277] Wang M., Sun J., Crosby A., Woodard K., Hirsch M.L., Samulski R.J., Li C. (2017). Direct interaction of human serum proteins with AAV virions to enhance AAV transduction: Immediate impact on clinical applications. Gene Ther..

[278] Denard J., Marolleau B., Jenny C., Rao T.N., Fehling H.J., Voit T., Svinartchouk F. (2013). C-reactive protein (CRP) is essential for efficient systemic transduction of recombinant adeno-associated virus vector 1 (rAAV-1) and rAAV-6 in mice. J. Virol..

[279] Pei X., He T., Hall N.E., Gerber D., Samulski R.J., Li C. (2018). AAV8 virions hijack serum proteins to increase hepatocyte binding for transduction enhancement. Virology.

[280] Fanali G., di Masi A., Trezza V., Marino M., Fasano M., Ascenzi P. (2012). Human serum albumin: From bench to bedside. Mol. Asp. Med..

[281] Yacobi A., Udall J.A., Levy G. (1976). Serum protein binding as a determinant of warfarin body clearance and anticoagulant effect. Clin. Pharmacol. Ther..

[282] Jessup M., Greenberg B., Mancini D., Cappola T., Pauly D.F., Jaski B., Yaroshinsky A., Zsebo K.M., Dittrich H., Hajjar R.J. (2011). Calcium Upregulation by Percutaneous Administration of Gene Therapy in Cardiac Disease (CUPID): A phase 2 trial of intracoronary gene therapy of sarcoplasmic reticulum Ca2+-ATPase in patients with advanced heart failure. Circulation.

[283] Sasano T., Kikuchi K., McDonald A.D., Lai S., Donahue J.K. (2007). Targeted high-efficiency, homogeneous myocardial gene transfer. J. Mol. Cell. Cardiol..

[284] Russell D.W., Alexander I.E., Miller A.D. (1995). DNA synthesis and topoisomerase inhibitors increase transduction by adeno-associated virus vectors. Proc. Natl. Acad. Sci. USA.

[285] Prasad K.M., Xu Y., Yang Z., Toufektsian M.C., Berr S.S., French B.A. (2007). Topoisomerase inhibition accelerates gene expression after adeno-associated virus-mediated gene transfer to the mammalian heart. Mol. Ther. J. Am. Soc. Gene Ther..

[286] Duan D., Yue Y., Yan Z., Yang J., Engelhardt J.F. (2000). Endosomal processing limits gene transfer to polarized airway epithelia by adeno-associated virus. J. Clin. Investig..

[287] Douar A.M., Poulard K., Stockholm D., Danos O. (2001). Intracellular trafficking of adeno-associated virus vectors: Routing to the late endosomal compartment and proteasome degradation. J. Virol..

[288] Yan Z., Zak R., Luxton G.W., Ritchie T.C., Bantel-Schaal U., Engelhardt J.F. (2002). Ubiquitination of both adeno-associated virus type 2 and 5 capsid proteins affects the transduction efficiency of recombinant vectors. J. Virol..

[289] Pandha H.S., Heinemann L., Simpson G.R., Melcher A., Prestwich R., Errington F., Coffey M., Harrington K.J., Morgan R. (2009). Synergistic effects of oncolytic reovirus and cisplatin chemotherapy in murine malignant melanoma. Clin. Cancer Res. Off. J. Am. Assoc. Cancer Res..

[290] Mitchell A.M., Li C., Samulski R.J. (2013). Arsenic trioxide stabilizes accumulations of adeno-associated virus virions at the perinuclear region, increasing transduction in vitro and in vivo. J. Virol..

[291] Nicolson S.C., Li C., Hirsch M.L., Setola V., Samulski R.J. (2016). Identification and Validation of Small Molecules That Enhance Recombinant Adeno-associated Virus Transduction following High-Throughput Screens. J. Virol..

[292] Chai Z., Zhang X., Dobbins A.L., Samulski R.J., Merricks E.P., Nichols T.C., Li C. (2022). Dexamethasone Transiently Enhances Transgene Expression in the Liver When Administered at Late-Phase Post Long-Term Adeno-Associated Virus Transduction. Hum. Gene Ther..

[293] Rebuffat A., Bernasconi A., Ceppi M., Wehrli H., Verca S.B., Ibrahim M., Frey B.M., Frey F.J., Rusconi S. (2001). Selective enhancement of gene transfer by steroid-mediated gene delivery. Nat. Biotechnol..

[294] Kelly A.M., Plautz S.A., Zempleni J., Pannier A.K. (2016). Glucocorticoid Cell Priming Enhances Transfection Outcomes in Adult Human Mesenchymal Stem Cells. Mol. Ther. J. Am. Soc. Gene Ther..

[295] Hamann A., Kozisek T., Broad K., Pannier A.K. (2020). Glucocorticoid Priming of Nonviral Gene Delivery to hMSCs Increases Transfection by Reducing Induced Stresses. Molecular therapy. Methods Clin. Dev..

[296] Chen Y.W., Diamante G., Ding J., Nghiem T.X., Yang J., Ha S.M., Cohn P., Arneson D., Blencowe M., Garcia J. (2022). PharmOmics: A species- and tissue-specific drug signature database and gene-network-based drug repositioning tool. iScience.

[297] Wishart D.S., Feunang Y.D., Guo A.C., Lo E.J., Marcu A., Grant J.R., Sajed T., Johnson D., Li C., Sayeeda Z. (2018). DrugBank 5.0: A major update to the DrugBank database for 2018. Nucleic Acids Res..

[298] Yoo M., Shin J., Kim J., Ryall K.A., Lee K., Lee S., Jeon M., Kang J., Tan A.C. (2015). DSigDB: Drug signatures database for gene set analysis. Bioinformatics.

[299] Coroadinha A.S. (2023). Host Cell Restriction Factors Blocking Efficient Vector Transduction: Challenges in Lentiviral and Adeno-Associated Vector Based Gene Therapies. Cells.

[300] Mano M., Ippodrino R., Zentilin L., Zacchigna S., Giacca M. (2015). Genome-wide RNAi screening identifies host restriction factors critical for in vivo AAV transduction. Proc. Natl. Acad. Sci. USA.

[301] Zajkowska A., Czajka M., Gulik K., Gawrychowski K., Małecki M. (2023). Profiling of microRNA as a tool to introduce rAAV vectors in gene therapy of breast cancer: A preliminary report. Adv. Clin. Exp. Med. Off. Organ Wroc. Med. Univ..

[302] Wagner A.E., Huck G., Stiehl D.P., Jelkmann W., Hellwig-Bürgel T. (2008). Dexamethasone impairs hypoxia-inducible factor-1 function. Biochem. Biophys. Res. Commun..

[303] Szklarczyk D., Gable A.L., Lyon D., Junge A., Wyder S., Huerta-Cepas J., Simonovic M., Doncheva N.T., Morris J.H., Bork P. (2019). STRING v11: Protein-protein association networks with increased coverage, supporting functional discovery in genome-wide experimental datasets. Nucleic Acids Res..

[304] STRING DATABASE. https://string-db.org/.

[305] Ripa I., Andreu S., López-Guerrero J.A., Bello-Morales R. (2021). Membrane Rafts: Portals for Viral Entry. Front. Microbiol..

[306] Pike L.J. (2006). Rafts defined: A report on the Keystone Symposium on Lipid Rafts and Cell Function. J. Lipid Res..

[307] Kahle M., Schäfer A., Seelig A., Schultheiß J., Wu M., Aichler M., Leonhardt J., Rathkolb B., Rozman J., Sarioglu H. (2015). High fat diet-induced modifications in membrane lipid and mitochondrial-membrane protein signatures precede the development of hepatic insulin resistance in mice. Mol. Metab..

[308] Calder P.C. (2006). n-3 polyunsaturated fatty acids, inflammation, and inflammatory diseases. Am. J. Clin. Nutr..

[309] Pike L.J., Han X., Chung K.N., Gross R.W. (2002). Lipid rafts are enriched in arachidonic acid and plasmenylethanolamine and their composition is independent of caveolin-1 expression: A quantitative electrospray ionization/mass spectrometric analysis. Biochemistry.

[310] Chan Y.K., Wang S.K., Chu C.J., Copland D.A., Letizia A.J., Costa Verdera H., Chiang J.J., Sethi M., Wang M.K., Neidermyer W.J. (2021). Engineering adeno-associated viral vectors to evade innate immune and inflammatory responses. Sci. Transl. Med..

[311] Rooks M.G., Garrett W.S. (2016). Gut microbiota, metabolites and host immunity. Nat. Rev. Immunol..

[312] Telle-Hansen V.H., Holven K.B., Ulven S.M. (2018). Impact of a healthy dietary pattern on gut microbiota and systemic inflammation in humans. Nutrients.

[313] Tan K., Zhang H., Lim L.-S., Ma H., Li S., Zheng H. (2020). Roles of carotenoids in invertebrate immunology. Front. Immunol..

[314] Baeke F., Takiishi T., Korf H., Gysemans C., Mathieu C. (2010). Vitamin D: Modulator of the immune system. Curr. Opin. Pharmacol..

[315] Zhang Y., Roh Y.J., Han S.-J., Park I., Lee H.M., Ok Y.S., Lee B.C., Lee S.-R. (2020). Role of selenoproteins in redox regulation of signaling and the antioxidant system: A review. Antioxidants.

[316] Heo Y.A. (2023). Etranacogene Dezaparvovec: First Approval. Drugs.

[317] Kardani K., Milani A., Shabani S.H., Bolhassani A. (2019). Cell penetrating peptides: The potent multi-cargo intracellular carriers. Expert Opin. Drug Deliv..

[318] Samaranch L., Salegio E.A., San Sebastian W., Kells A.P., Foust K.D., Bringas J.R., Lamarre C., Forsayeth J., Kaspar B.K., Bankiewicz K.S. (2011). Adeno-Associated Virus Serotype 9 Transduction in the Central Nervous System of Nonhuman Primates. Hum. Gene Ther..

[319] Kawai T., Akira S. (2007). Signaling to NF-κB by Toll-like receptors. Trends Mol. Med..

[320] Ivashkiv L.B., Donlin L.T. (2014). Regulation of type I interferon responses. Nature reviews. Immunology.

[321] Ostrycharz E., Hukowska-Szematowicz B. (2022). New Insights into the Role of the Complement System in Human Viral Diseases. Biomolecules.

[322] Libbey J.E., Fujinami R.S., Tselis A.C., Booss J. (2014). Chapter 10—Adaptive immune response to viral infections in the central nervous system. Handbook of Clinical Neurology.

[323] Nathwani A.C., Tuddenham E.G., Rangarajan S., Rosales C., McIntosh J., Linch D.C., Chowdary P., Riddell A., Pie A.J., Harrington C. (2011). Adenovirus-associated virus vector-mediated gene transfer in hemophilia B. N. Engl. J. Med..

[324] Smith C.J., Ross N., Kamal A., Kim K.Y., Kropf E., Deschatelets P., Francois C., Quinn W.J., Singh I., Majowicz A. (2022). Pre-existing humoral immunity and complement pathway contribute to immunogenicity of adeno-associated virus (AAV) vector in human blood. Front. Immunol..

[325] Apellis Pharmaceuticals Will Commence APL-9 Program to Control the Complement System in Host Responses to AAV Vector Administration for Gene Therapies. https://www.globenewswire.com/news-release/2019/07/18/1884481/0/en/Apellis-Pharmaceuticals-Will-Commence-APL-9-Program-to-Control-the-Complement-System-in-Host-Responses-to-AAV-Vector-Administration-for-Gene-Therapies.html.

[326] Legendre C.M., Licht C., Muus P., Greenbaum L.A., Babu S., Bedrosian C., Bingham C., Cohen D.J., Delmas Y., Douglas K. (2013). Terminal complement inhibitor eculizumab in atypical hemolytic-uremic syndrome. N. Engl. J. Med..

[327] Muhuri M., Zhan W., Maeda Y., Li J., Lotun A., Chen J., Sylvia K., Dasgupta I., Arjomandnejad M., Nixon T. (2021). Novel Combinatorial MicroRNA-Binding Sites in AAV Vectors Synergistically Diminish Antigen Presentation and Transgene Immunity for Efficient and Stable Transduction. Front. Immunol..

[328] Byrne B.J., Fuller D.D., Smith B.K., Clement N., Coleman K., Cleaver B., Vaught L., Falk D.J., McCall A., Corti M. (2019). Pompe disease gene therapy: Neural manifestations require consideration of CNS directed therapy. Ann. Transl. Med..

[329] Mingozzi F., Hasbrouck N.C., Basner-Tschakarjan E., Edmonson S.A., Hui D.J., Sabatino D.E., Zhou S., Wright J.F., Jiang H., Pierce G.F. (2007). Modulation of tolerance to the transgene product in a nonhuman primate model of AAV-mediated gene transfer to liver. Blood.

[330] Peters C.W., Maguire C.A., Hanlon K.S. (2021). Delivering AAV to the central nervous and sensory systems. Trends Pharmacol. Sci..

[331] Ilyinskii P.O., Michaud A.M., Roy C.J., Rizzo G.L., Elkins S.L., Capela T., Chowdhury A.C., Leung S.S., Kishimoto T.K. (2021). Enhancement of liver-directed transgene expression at initial and repeat doses of AAV vectors admixed with ImmTOR nanoparticles. Sci. Adv..

[332] Meliani A., Boisgerault F., Hardet R., Marmier S., Collaud F., Ronzitti G., Leborgne C., Costa Verdera H., Simon Sola M., Charles S. (2018). Antigen-selective modulation of AAV immunogenicity with tolerogenic rapamycin nanoparticles enables successful vector re-administration. Nat. Commun..

[333] Bentler M., Hardet R., Ertelt M., Rudolf D., Kaniowska D., Schneider A., Vondran F.W.R., Schoeder C.T., Delphin M., Lucifora J. (2023). Modifying immune responses to adeno-associated virus vectors by capsid engineering. Molecular therapy. Methods Clin. Dev..

[334] Tseng Y.-S., Van Vliet K., Rao L., McKenna R., Byrne B.J., Asokan A., Agbandje-McKenna M. (2016). Generation and characterization of anti-adeno-associated virus serotype 8 (AAV8) and anti-AAV9 monoclonal antibodies. J. Virol. Methods.

[335] Tse L.V., Klinc K.A., Madigan V.J., Castellanos Rivera R.M., Wells L.F., Havlik L.P., Smith J.K., Agbandje-McKenna M., Asokan A. (2017). Structure-guided evolution of antigenically distinct adeno-associated virus variants for immune evasion. Proc. Natl. Acad. Sci. USA.

[336] Selot R., Arumugam S., Mary B., Cheemadan S., Jayandharan G.R. (2017). Optimized AAV rh. 10 vectors that partially evade neutralizing antibodies during hepatic gene transfer. Front. Pharmacol..

[337] Mével M., Bouzelha M., Leray A., Pacouret S., Guilbaud M., Penaud-Budloo M., Alvarez-Dorta D., Dubreil L., Gouin S.G., Combal J.P. (2019). Chemical modification of the adeno-associated virus capsid to improve gene delivery. Chem. Sci..

[338] Hoang Thi T.T., Pilkington E.H., Nguyen D.H., Lee J.S., Park K.D., Truong N.P. (2020). The Importance of Poly(ethylene glycol) Alternatives for Overcoming PEG Immunogenicity in Drug Delivery and Bioconjugation. Polymers.

[339] Meliani A., Boisgerault F., Fitzpatrick Z., Marmier S., Leborgne C., Collaud F., Simon Sola M., Charles S., Ronzitti G., Vignaud A. (2017). Enhanced liver gene transfer and evasion of preexisting humoral immunity with exosome-enveloped AAV vectors. Blood Adv..

